# γ-Fe_2_O_3_@Zn-LDH-EAE-SO_3_H for multi-component synthesis of chromeno[4,3-*b*]quinoline-6,8-dione derivatives†

**DOI:** 10.1039/d5ra03659c

**Published:** 2025-06-24

**Authors:** Ahad Vatandoust Namanloo, Batool Akhlaghnia

**Affiliations:** a Department of Chemistry, Faculty of Science, Ferdowsi University of Mashhad Mashhad 9177948974 Iran akhlaghinia@um.ac.ir

## Abstract

In this study, 2-aminoethanesulfonic acid immobilized on epichlorohydrin-functionalized γ-Fe_2_O_3_@Zn-LDH (γ-Fe_2_O_3_@Zn-LDH-EAE-SO_3_H) was synthesized as a new and efficient magnetic nanostructured catalyst for the rapid synthesis of chromeno[4,3-*b*]quinoline-6,8-dione derivatives. The nanostructured catalyst was subsequently characterized using various techniques including FT-IR, XRD, TEM, FE-SEM, EDX, EDX-mapping, TGA, CHNS and VSM analyses. The characterization results confirmed the composition of γ-Fe_2_O_3_@Zn-LDH-EAE-SO_3_H and demonstrated that the magnetic nanoparticles were spherical in shape with average particle sizes ranging from 5 to 17 nm. As an excellent alternative to Brønsted acids, γ-Fe_2_O_3_@Zn-LDH-EAE-SO_3_H exhibited high efficiency in the multi-component synthesis of chromeno[4,3-*b*]quinoline-6,8-dione derivatives in green media. In comparison to the conventional methods, this protocol presented several key benefits such as reduced reaction times, mild reaction conditions, simple work-up processes, pure products with high yields, easy catalyst recovery with an external magnetic field and the capability to reuse the catalyst several times without any significant loss in its catalytic activity. In addition, a notable feature of the present protocol was the use of ethanol as an environmentally friendly solvent, eliminating the use of toxic solvents.

## Abstract 2

A novel method for preparing chromeno[4,3-*b*]quinoline-6,8-dione derivatives with high yield of products in the presence of γ-Fe_2_O_3_@Zn-LDH@EAE-SO_3_H as heterogeneous nanostructured catalyst.

## Graphical abstract

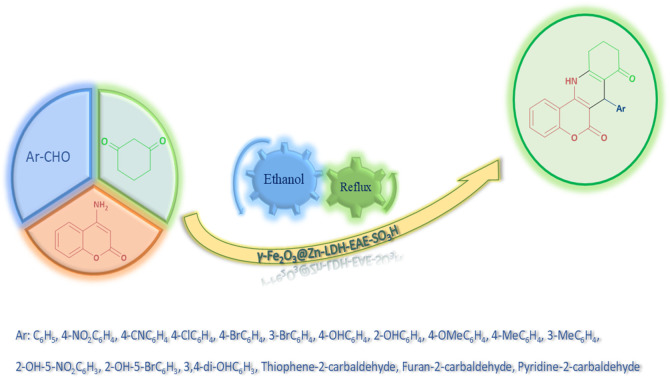

## 1. Introduction

Quinoline and chromene are two fused bicyclic *N*- and *O*-heterocyclic compounds that possess valuable biological and pharmacological^1–7^ properties such as antioxidant,^8^ antimicrobial,^9^ antiproliferative,^10^ antitubercular,^11,22^ antibacterial,^12–14^ antitumor,^15^ anticonvulsant,^16^ antivascular,^17^ anticancer,^18^ anti-inflammatory,^19^ and antifungal^20,21^ activities. Upon the fusion of chromene and quinoline moieties, chromeno[4,3-*b*]quinoline-6,8-dione is constructed with additional unique biological properties such as antiviral,^23^ antitumor,^24^ antimicrobial,^25^ anti-inflammatory,^26^ neuroprotective,^27,28^ anti-proliferative,^29^ anticoccidial^30^ and nonsteroidal human progesterone receptor agonist^31,32^ activities.

Several homogeneous multi-component and cyclization methods have been documented according to various applications of chromeno[4,3-*b*]quinoline derivatives.^33–42^ Based on the prominent biological importance of chromeno[4,3-*b*]quinoline derivatives as useful precursors in the preparation of organic and medicinal compounds, heterogeneous multi-component and cyclization methods using Cu(ii)-Schiff base/SBA-15,^43^ SnO_2_ NPs,^44^ sulfonic acid-functionalized magnetic starch (Starch/SPION@SO_3_H)^45^ and bimetallic organic frameworks (Ni/Co-BTC BMOF)^46^ have also attracted much attention.

Layered double hydroxides (LDHs) or hydrotalcite-like (HT-like) compounds with two-dimensional (2D) brucite-like layer structures have the potential to be used as heterogeneous catalysts.^47,48^ LDHs are represented by the chemical formula [M^2+^_1−*x*_M^3+^_*x*_ (OH)_2_]^*x*+^ A^*n*−^·*z*H_2_O, in which M^2+^ and M^3+^ represent divalent and trivalent layer cations, respectively. The octahedral holes of the brucite structure can be occupied by M^2+^ and M^3+^ cations such as Mg^2+^, Ni^2+^, Cu^2+^, Zn^2+^, Al^3+^, and Fe^3+^, and the interlayer region contains A^n−^·*z*H_2_O, which includes exchangeable anions such as SO_4_^2−^, NO_3_^−^, CO_3_^2−^, and ClO^−^. The amount of *x* equals the molar ratio of M^2+^/M^2+^ + M^3+^, which is around (0.2–0.33).^49–53^ Owing to their fascinating physico-chemical properties (*i.e.* high thermal and chemical stability, biocompatibility, high specific surface area, anion exchangeability, composition flexibility and memory effect), LDHs have been considered in catalysis science in recent years.^54–59^ Today, LDHs have catalytic application in a wide range of organic reactions including Knoevenagel condensations,^60,61^ Claisen–Schmidt condensations,^62^ transesterification,^63,64^*N*-arylation of amines,^65^ Michael additions,^66^ oxidation of alcohols,^67,68^ cyanoethylation of alcohols,^69^ aldol condensations^70,71^ and epoxidation.^72,73^

Considering the ongoing need to separate the catalyst from the reaction medium, magnetic nanomaterials have been developed to simplify reaction workup, facilitate catalyst recovery and decrease the reaction time, energy and additional materials used. Therefore, most reports about magnetic LDH compounds have mentioned their uses as supported magnetic nanoparticles (MNPs).^74–79^ To date, a number of recent scientific research studies have revealed the combination of LDHs and magnetic nanoparticles such as Fe_3_O_4_@MgAl-LDH,^80^ γ-Fe_2_O_3_@Cu_3_Al-LDH-namidinoglycine,^81^ γ-Fe_2_O_3_@Cu_3_Al-LDH/HEPES,^82^ immobilized Zn–Ni–Fe LDHs on silica-coated magnetite,^83^ LDH-based CoO–NiFe_2_O_4_,^84^ Co complexes of PCE immobilized on γ-Fe_2_O_3_@Cu_3_Al-LDH,^85^ γ-Fe_2_O_3_@Cu-LDH intercalated with palladium cysteine,^86^ Zn(ii) doped and immobilized on functionalized magnetic hydrotalcite (Fe_3_O_4_/HT-SMTU-Zn(ii))^87^ and tannic acid immobilized on functionalized magnetic hydrotalcite Fe_3_O_4_/HT-GLYMO-TA.^88^

**Scheme 1:**
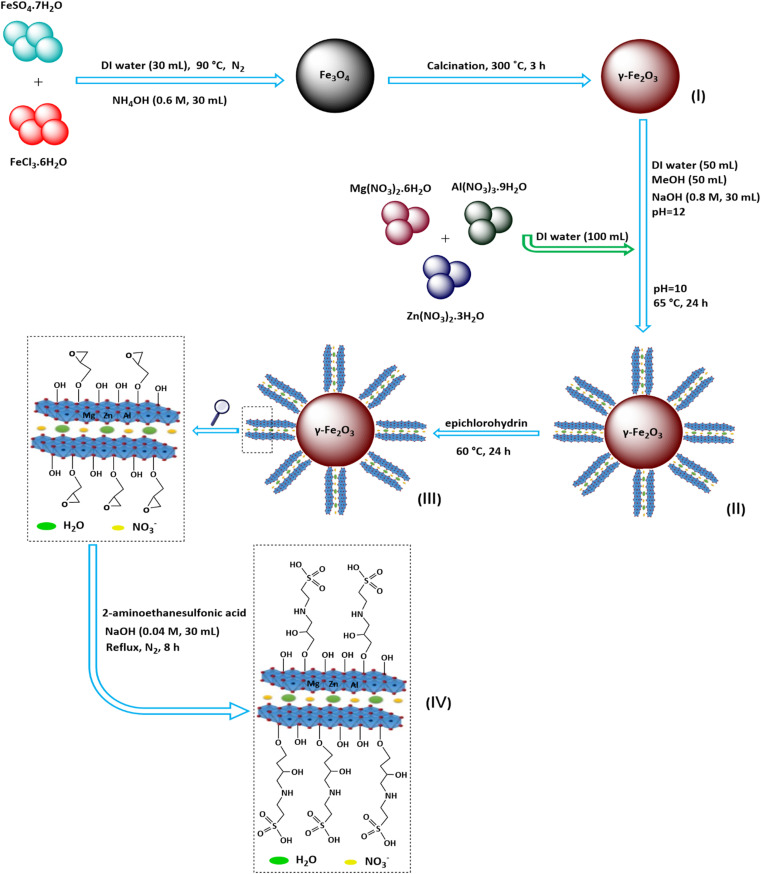
Preparation steps of γ-Fe_2_O_3_@Zn-LDH-EAE-SO_3_H (IV).

In continuation of our previous research,^89–91^ in the present study, 2-aminoethanesulfonic acid immobilized on epichlorohydrin-functionalized γ-Fe_2_O_3_@Zn-LDH (γ-Fe_2_O_3_@Zn-LDH-EAE-SO_3_H (IV)) was prepared according to the steps presented in Scheme 1. First of all, γ-Fe_2_O_3_ (I) nanoparticles were obtained through a facile one-pot coprecipitation method using FeSO_4_·7H_2_O and FeCl_3_·6H_2_O salts in an alkaline solution followed by calcination at 300 °C.^49,92^ Upon reaction with Mg(NO_3_)_2_·6H_2_O, Al(NO_3_)_3_·9H_2_O and Zn(NO_3_)_2_·3H_2_O salt solutions, core–shell structured γ-Fe_2_O_3_@Zn-LDH (II) was obtained.^93^ Suspension of II in epichlorohydrin at 60 °C with vigorous stirring yields epichlorohydrin-functionalized γ-Fe_2_O_3_@Zn-LDH (γ-Fe_2_O_3_@Zn-LDH-E) (III). Afterwards, γ-Fe_2_O_3_@Zn-LDH-EAE-SO_3_H (IV) was synthesized by treating III with 2-aminoethanesulfonic acid (taurine, a bifunctional organic molecule containing both –SO_3_H and –NH_2_ groups).^89^

## 2. Experimental

### 2.1 General

All chemical reagents and solvents were purchased from Merck Chemical Companies and used as received without further purification. The purity determinations of the products and the progress of the reactions were accomplished by TLC on silica gel polygram STL G/UV 254 plates. The melting points of the products were determined using an Electrothermal Type 9100 melting point apparatus. The FT-IR spectra were recorded using an AVATAR 370 FT-IR spectrometer (Therma Nicolet spectrometer, USA) with pressed KBr pellets at room temperature in the range between 4000 and 400 cm^−1^ with a resolution of 4 cm^−1^. The NMR spectra were recorded using a Brucker Avance 300 MHz instrument with DMSO-*d*_6_ as the solvent. Mass spectra were recorded using a CH7A Varianmat Bremem instrument at 70 eV electron impact ionization, in *m*/*z* (rel%). Elemental analyses were performed using a Thermo Finnigan Flash EA 1112 Series instrument. The crystalline structure of the catalyst was analyzed by small-angle XRD using a PANalytical Company X'Pert PRO MPD diffractometer operating at 40 kV and 40 mA, using Cu Ka radiation (*λ* = 0.154 nm) (at a step size of 0.02° and a step time of 2 s). Transmission electron microscopy (TEM) was performed using a Leo 912 AB microscope (Zeiss, Germany) at an accelerating voltage of 120 kV. The FE-SEM images, EDX spectra and EDX mapping were recorded using a TESCAN model: MIRA3 scanning electron microscope (manufactured in the Czech Republic) operating at an accelerating voltage of 30.0 kV at resolutions of about 200 nm, 500 nm and 1 mm. TGA was performed using a Shimadzu Thermogravimetric Analyzer (TG-50) in the temperature range of 25–950 °C at a heating rate of 10 °C min^−1^ under an air atmosphere. The magnetic property of the catalyst was measured using a vibrating sample magnetometer (VSM, Magnetic Danesh pajoh Inst). All yields refer to isolated products after purification by column chromatography.

### 2.2 Preparation of magnetite nanoparticles (γ-Fe_2_O_3_ NPs (I))

FeSO_4_·7H_2_O (0.88 g, 3.2 mmol) and FeCl_3_·6H_2_O (1.5 g, 5.6 mmol) were dissolved in deionized water (30 mL) and heated at 90 °C under a N_2_ atmosphere. A NH_4_OH solution (0.6 M, 30 mL) was added dropwise to the mixture to adjust the reaction pH to 11. The resulting solution was continuously stirred at 90 °C for 1 h and then cooled to room temperature. The magnetic Fe_3_O_4_ nanoparticles were separated by a magnetic bar and washed with deionized water until it was neutralized.^92^ Then, the obtained nanoparticles were calcined under an oxygen atmosphere at 300 °C for 3 h to provide γ-Fe_2_O_3_ nanoparticles (γ-Fe_2_O_3_ NPs) (I).^49^

### 2.3 Preparation of magnetite nanoparticles coated with a layered double hydroxide shell (γ-Fe_2_O_3_@Zn-LDH(ii))

γ-Fe_2_O_3_ (I) (1.0 g) was dispersed in deionized water (50 mL) and methanol (50 mL) for 30 min under ultrasonic irradiation. Then, an aqueous solution of NaOH (0.8 M, 30 mL) was added dropwise to the reaction mixture until the pH reached 12. Next, a solution of Mg(NO_3_)_2_·6H_2_O (1.92 g, 7.5 mmol), Al(NO_3_)_3_·9H_2_O (1.12 g, 3 mmol) and Zn(NO_3_)_2_·3H_2_O (0.44 g, 1.5 mmol) in deionized water (100 mL) was added dropwise into the above suspension to reach a pH of 10. Then, the reaction mixture was stirred at 65 °C for 24 h. Finally, the resulting magnetite nanoparticles coated with a layered double hydroxide shell (γ-Fe_2_O_3_@Zn-LDH (II)) as a dark brown product was separated with an external magnetic field and washed, in turn, with ethanol and deionized water for several times and dried at 65 °C for 24 h.^47^

### 2.4 Preparation of epichlorohydrin-functionalized γ-Fe_2_O_3_@Zn-LDH (γ-Fe_2_O_3_@Zn-LDH-E (III))

γ-Fe_2_O_3_@Zn-LDH (II) (0.7 g) was dispersed in 6 mL epichlorohydrin by sonication for 45 min. Afterwards, the suspension was heated at 60 °C for 24 h to obtain epichlorohydrin-functionalized γ-Fe_2_O_3_@Zn-LDH (γ-Fe_2_O_3_@Zn-LDH-E (III)). The precipitate was separated by an external magnetic field and washed with MeOH (4× 10 mL) till removing the additional amount of epichlorohydrin before drying at 50 °C under vacuum for 10 h.^89^

### 2.5 Preparation of 2-aminoethanesulfonic acid immobilized on epichlorohydrin-functionalized γ-Fe_2_O_3_@Zn-LDH (γ-Fe_2_O_3_@Zn-LDH-EAE-SO_3_H(IV))

γ-Fe_2_O_3_@Zn-LDH-E (III) (0.5 g) was added to a solution of 2-aminoethanesulfonic acid (0.7 g, 5 mmol) in refluxing NaOH (0.04 M, 30 mL) under a N_2_ atmosphere for 8 h. Then, resulting 2-aminoethanesulfonic acid immobilized on epichlorohydrin-functionalized γ-Fe_2_O_3_@Zn-LDH-EAE-SO_3_H (IV) as a dark brown precipitate was separated using a magnetic bar and washed with distilled water before drying at 60 °C overnight under vacuum.^89^

### 2.6 Synthesis of 4-aminocoumarin

A mixture of 4-hydroxycoumarin (1.07 g, 0.066 mol) and ammonium acetate (7.87 g, 0.1 mol) was melted in an oil bath (max. 130°C) and stirred for 5 h. After completion of the reaction (monitored by TLC), the resulting mixture was cooled to ambient temperature. Then, distilled water (100 mL) was added and 4-aminocoumarin was collected by simple filtration and recrystallized from EtOH : H_2_O (2 : 1) (0.96 g, 90%).^33^

### 2.7 Typical procedure for the synthesis of 7-phenyl-9,10,11,12-tetrahydro-6*H*-chromeno[4,3-*b*]quinoline-6,8(7*H*)-dione in the presence of γ-Fe_2_O_3_ @Zn-LDH-EAE-SO_3_H (IV)

To a stirring solution of benzaldehyde (0.106 g, 1 mmol), 1,3-cyclohexanedione (0.112 g, 1 mmol) and 4-aminocoumarin (0.161 g, 1 mmol) in refluxing ethanol, γ-Fe_2_O_3_@Zn-LDH-EAE-SO_3_H(IV) (20 mg) was added. After completion of the reaction, which was monitored by TLC (*n*-hexane : MeOH, 7 : 3), the magnetic catalyst was separated by a magnetic field, washed with hot ethyl acetate and dried at 60 °C for 2 h for the next run use. Afterwards, the reaction mixture was cooled to room temperature and the crude product was filtered. 7-Phenyl-7,10,11,12-tetrahydro-*6H*-chromeno[4,3-*b*]quinoline-6,8(*9H*)-dione as a light-yellow solid was purified by column chromatography using *n*-hexane : MeOH, (4 : 1, v/v) as eluent (0.325 g, 95%).

## 3. Results and discussion

### 3.1 Characterization of γ-Fe_2_O_3_@Zn-LDH-EAE-SO_3_H (IV)

γ-Fe_2_O_3_@Zn-LDH-EAE-SO_3_H (IV) as a new magnetically heterogeneous catalyst was synthesized, according to the pathway shown in Scheme 1. To confirm the structure and composition of the synthesized nanostructured catalyst, some microscopic and spectroscopic analytical techniques (FT-IR spectroscopy, XRD, TEM, SEM, EDX, EDX-mapping, TGA, CHNS, and VSM) were performed. The results obtained from these techniques showed the successful preparation of the nanostructured catalyst.

**Fig. 1:**
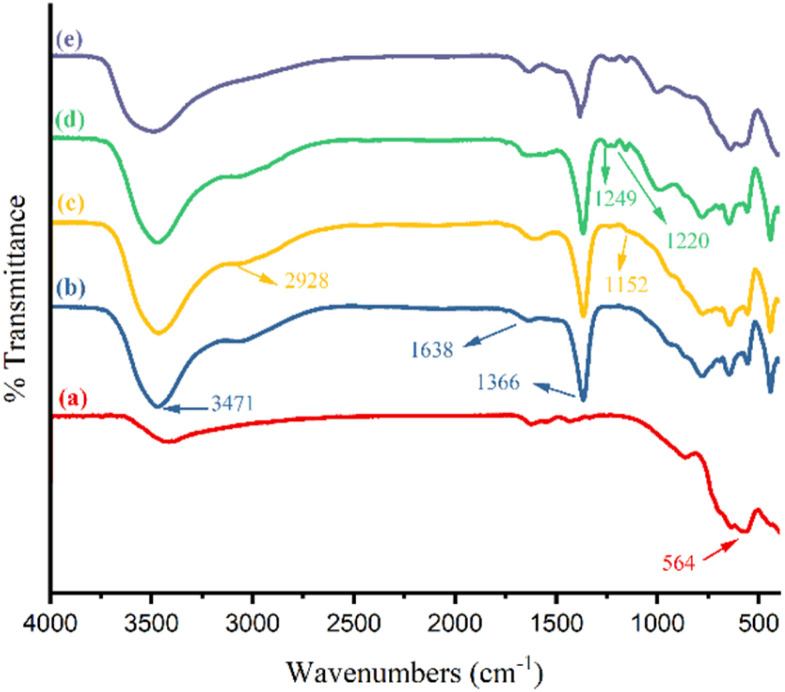
FT-IR spectra of γ-Fe_2_O_3_ nanoparticles (I) (a), γ-Fe_2_O_3_@Zn-LDH (II) (b), γ-Fe_2_O_3_@Zn-LDH-E (III) (c) and γ-Fe_2_O_3_@Zn-LDH-EAE-SO_3_H (IV) (d) and the 4th reused γ-Fe_2_O_3_@Zn-LDH-EAE-SO_3_H (IV) (e).

Fig. 1 illustrates the FT-IR spectra of γ-Fe_2_O_3_ nanoparticles (I) (a), γ-Fe_2_O_3_@Zn-LDH(ii) (b), γ-Fe_2_O_3_@Zn-LDH-E (III) (c), γ-Fe_2_O_3_@Zn-LDH-EAE-SO_3_H(iv) (d) and the 4th reused γ-Fe_2_O_3_@Zn-LDH-EAE-SO_3_H(iv) (e). As can be seen, the FT-IR spectrum of γ-Fe_2_O_3_ (I) (Fig. 1(a)) showed an absorption band at 564 cm^−1^ attributed to the stretching vibration of the Fe–O bond.^47^ The presence of M–OH (M: Zn, Mg, and Al) bonds of γ-Fe_2_O_3_@Zn-LDH (II) was confirmed by the stretching vibration (Fig. 1(b)) at 3471 cm^−1^. The absorption band at 1638 cm^−1^ corresponds to the bending vibration of the hydroxyl groups of interlayer water molecules in the LDHs.^47^ Additionally, the bands at 1366 cm^−1^ and around 500–900 cm^−1^ illustrate the vibrating modes of the existing nitrate anion between LDH layers as well as Zn–O, Mg–O and Al–O groups (Fig. 1(b)).^83^Fig. 1(c) demonstrates the vibrational stretching of aliphatic CH_2_ and C–O–C linkage at 2928 cm^−1^ and 1152 cm^−1^, respectively.^89^ The obtained results proved the surface immobilization of γ-Fe_2_O_3_@Zn-LDH (II) by epichlorohydrin (Fig. 1(c)).^89^ The characteristic bands that appeared at 1249 cm^−1^ and 1220 cm^−1^ exhibited the C–N and S=O bonds, upon the ring opening of epoxy by 2-aminoethanesulfonic acid (Fig. 1(d)).^89^

**Fig. 2:**
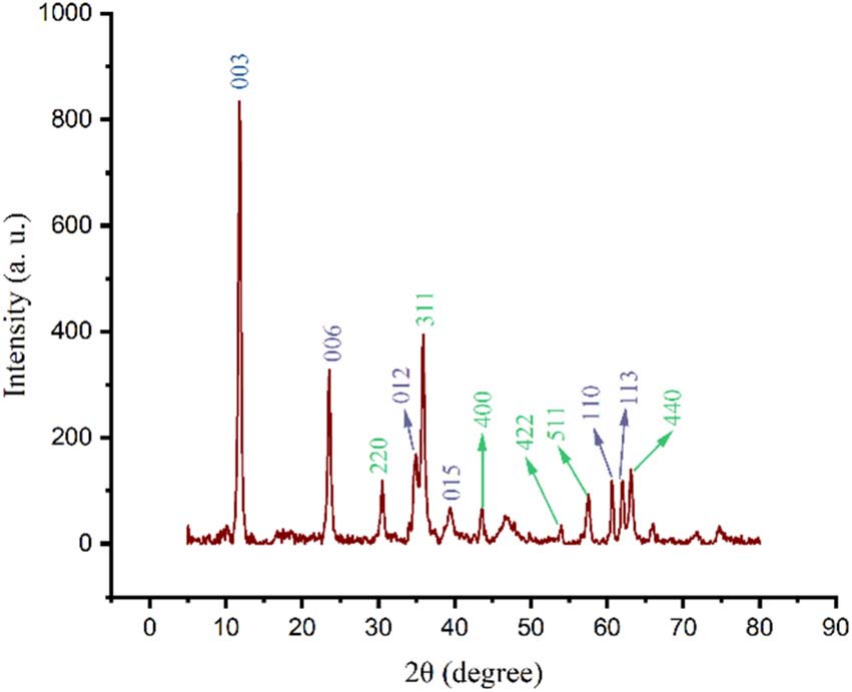
XRD pattern of γ-Fe_2_O_3_@Zn-LDH (II).

The XRD technique was used to identify the crystalline structure of γ-Fe_2_O_3_@Zn-LDH(ii), as shown in Fig. 2. The X-ray diffraction pattern exhibited a series of sharp lines at 2*θ* = 30.49°, 35.85°, 43.52°, 53.92°, 57.52° and 63.03°, which can be indexed to the (220), (311), (400), (422), (511) and (440) pure phases of maghemite nanoparticles (γ-Fe_2_O_3_) (JCPDF NO: 01-075-0449),^82^ respectively. In addition, diffraction peaks appeared at 2*θ* = 11.79°, 23.45°, 34.75°, 39.34°, 60.06° and 61.82° corresponding to the (003), (006), (012), (015), (110) and (113) crystallographic facets of the Zn-LDH phase, which are in agreement with the standard reported patterns (JCPDF NO: 01-080-4199).^94^ The reflection planes of (003), (006), (110) and (113) are the particular characteristics of LDH-like materials.^95^ Furthermore, the Williamson–Hall plot revealed that the average crystallite size of γ-Fe_2_O_3_@Zn-LDH (II) was estimated to be 14.3 nm.

**Fig. 3:**
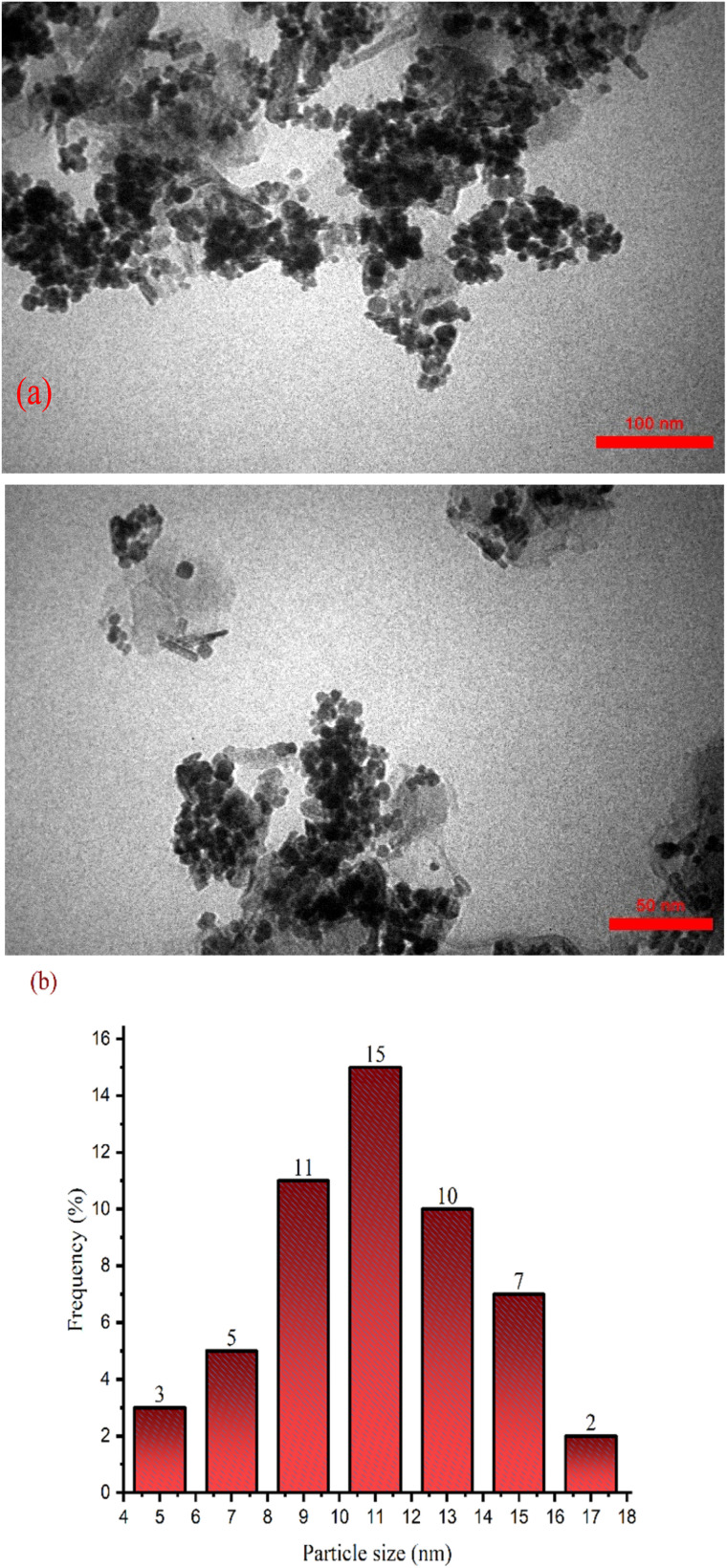
TEM images of γ-Fe_2_O_3_@Zn-LDH-EAE-SO_3_H (IV) (a) and particle size distribution histogram of γ-Fe_2_O_3_@Zn-LDH-EAE-SO_3_H (IV) (b).

The morphology and histogram of the particle size distribution of γ-Fe_2_O_3_@Zn-LDH-EAE-SO_3_H (IV) were investigated by a TEM technique, as shown in Fig. 3(a). As can be recognized from the TEM images, the synthesized catalyst has a spherical morphology with good monodispersity. The layer structure of the LDHs covers the dispersed spherical core of maghemite nanoparticles (γ-Fe_2_O_3_ NPs (I)), and the size of particles is in the range of 5–17 nm. Additionally, a distribution histogram of γ-Fe_2_O_3_@Zn-LDH-EAE-SO_3_H (IV) indicated that the average diameter of the nanoparticles is 15 nm, which is in good agreement with the mean particle size obtained from XRD analysis (Fig. 3(b)).

**Fig. 4:**
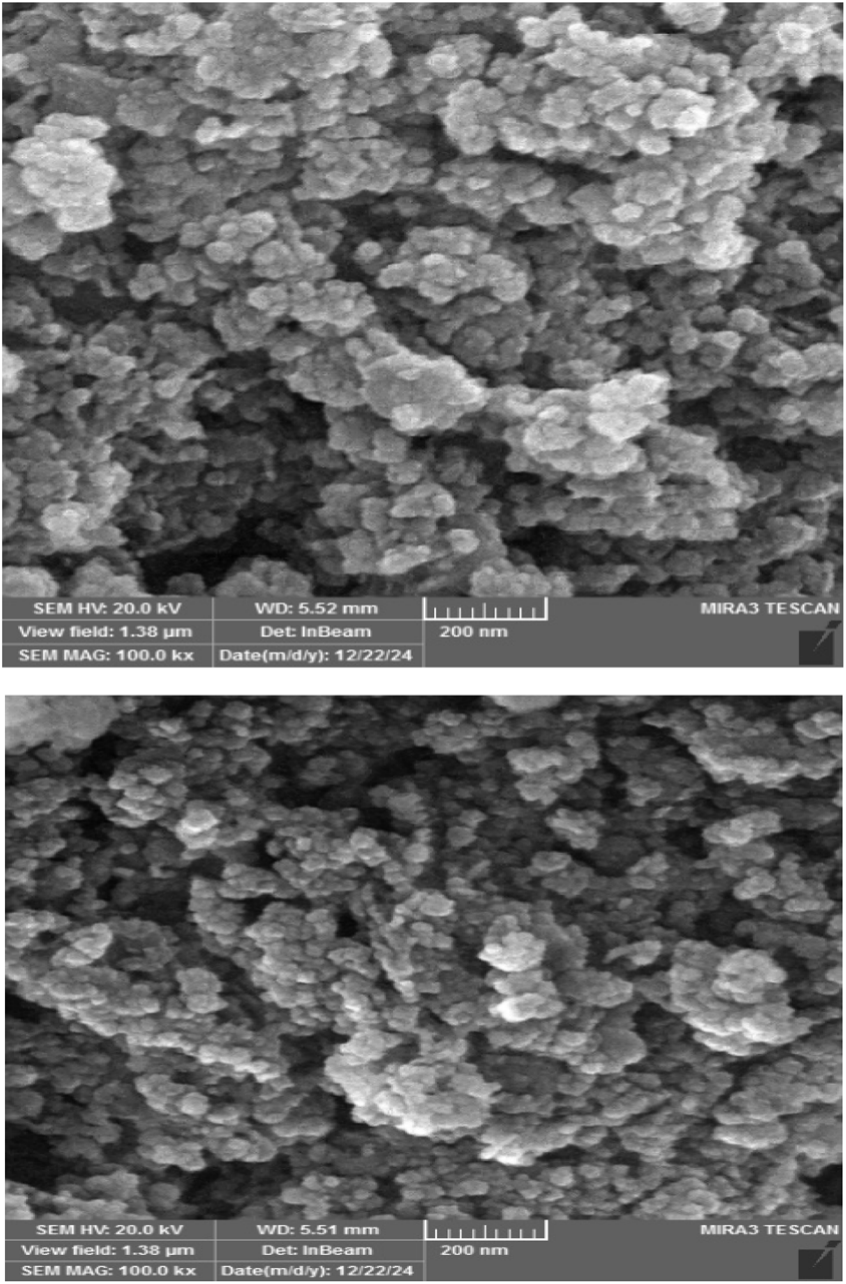
FE-SEM images of γ-Fe_2_O_3_@Zn-LDH-EAE-SO_3_H (IV).

**Fig. 5:**
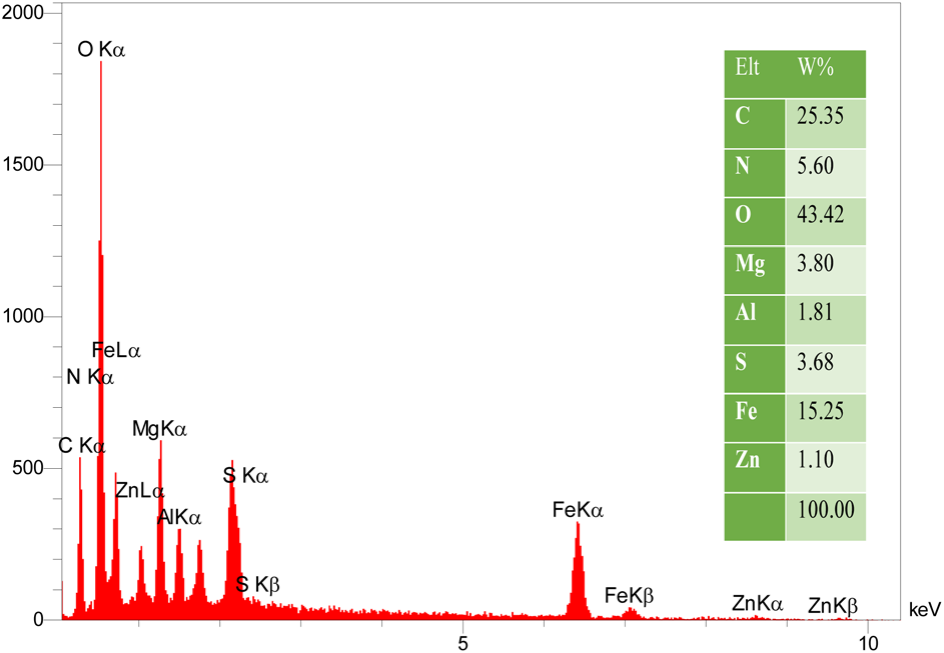
EDX spectrum of γ-Fe_2_O_3_@Zn-LDH-EAE-SO_3_H (IV).

**Fig. 6:**
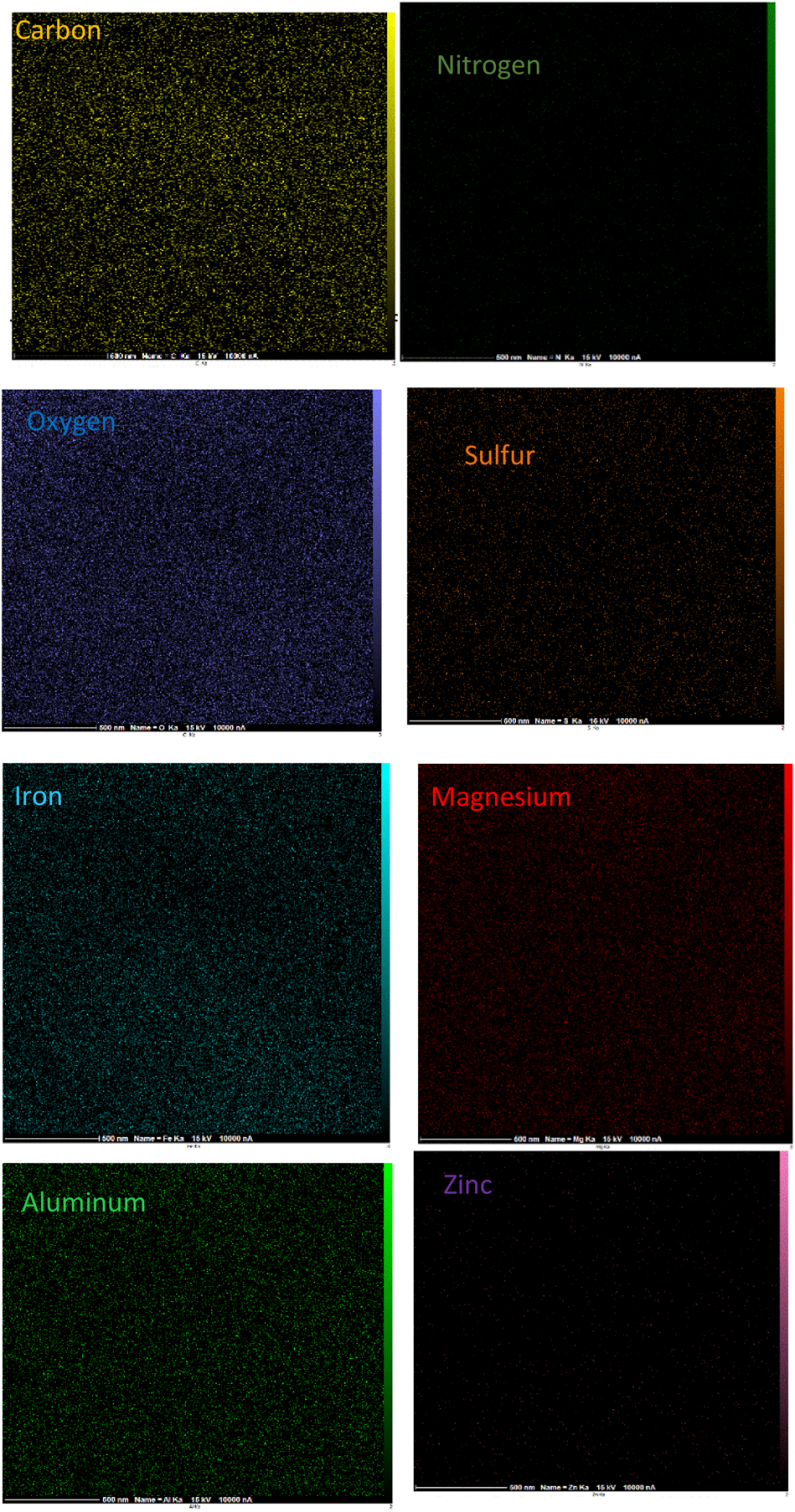
EDX-mapping analysis of γ-Fe_2_O_3_@Zn-LDH-EAE-SO_3_H (IV).

The surface morphology of γ-Fe_2_O_3_@Zn-LDH-EAE-SO_3_H (IV) was investigated by FE-SEM. As illustrated in Fig. 4, the nanoparticles have a spherical shape with uniform dispersion. The EDX analysis confirmed the presence of carbon, nitrogen, oxygen, sulfur, iron, magnesium, aluminum and zinc. Moreover, the EDX analysis verified no impure elements in the nanostructured catalyst. Furthermore, the considerable intensities of S and N clearly indicate that 2-aminoethanesulfonic acid was successfully immobilized on epichlorohydrin-functionalized γ-Fe_2_O_3_@Zn-LDH (γ-Fe_2_O_3_@Zn-LDH-E) (III) (Fig. 5). Moreover, the EDX-mapping images demonstrate the uniform distribution of C, N, O, S, Fe, Mg, Al and Zn elements in the catalyst structure (Fig. 6).

**Fig. 7:**
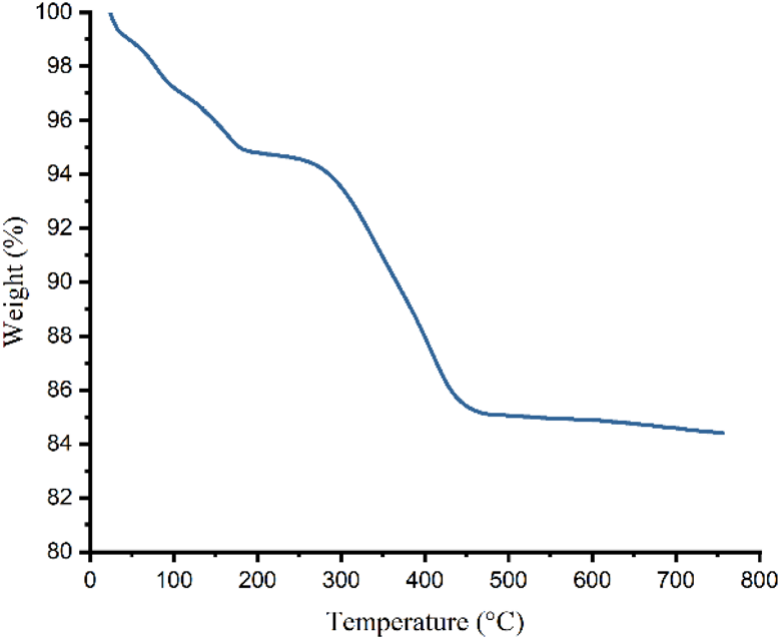
TGA thermogram of γ-Fe_2_O_3_@Zn-LDH-EAE-SO_3_H (IV).

Thermogravimetric analysis of γ-Fe_2_O_3_@Zn-LDH-EAE-SO_3_H (IV) reveals two weight loss steps (Fig. 7). The first one occurring between 55 and 200 °C (5.20%) corresponds to the evaporation of adsorbed water from the surface of the nanostructured catalyst. The second and main weight loss (10.22%) at 200–700 °C is attributed to the complete decomposition of the organic segments that are grafted onto the surface of the nanostructured catalyst. According to the TGA, the amounts of organic segments supported on γ-Fe_2_O_3_@Zn-LDH (II) were estimated to be 0.8 mmol g^−1^. According to the CHNS analysis data, the loading amounts of organic segments attached to γ-Fe_2_O_3_@Zn-LDH (II) was 0.76 mmol g^−1^ based on the carbon, nitrogen and sulfur contents (*C* = 5.23%, *N* = 1.09% and *S* = 2.43%).

The back-titration analysis was performed to determine the loading amounts of acidic sites (sulfonic groups) on the surface of γ-Fe_2_O_3_@Zn-LDH-EAE-SO_3_H (IV). To this purpose, 100 mg of the prepared nanostructured catalyst was suspended in an aqueous solution of NaOH (0.1 M, 15 mL), and then it was stirred at room temperature overnight. Afterwards, the suspension was filtered, and subsequently, the filtrate was neutralized using a 0.1 M standard solution of HCl. The amount of loaded –NHCH_2_CH_2_SO_3_H per 1.000 g of γ-Fe_2_O_3_@Zn-LDH-EAE-SO_3_H (IV) was calculated as 0.8 mmol (by the consumed volume of HCl (14.20 mL)). The outcomes of the back-titration analysis align well with the results obtained from thermogravimetric analysis (TGA) and elemental analysis data (CHNS).

**Fig. 8:**
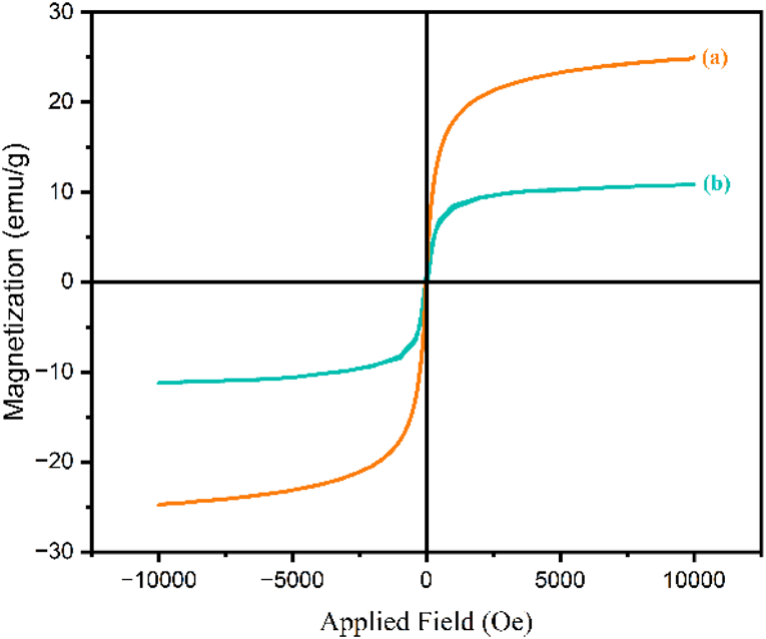
Magnetization curves of γ-Fe_2_O_3_@Zn-LDH (II) (a) and γ-Fe_2_O_3_@Zn-LDH-EAE-SO_3_H (IV) (b).

The magnetization curves of γ-Fe_2_O_3_@Zn-LDH (II) and γ-Fe_2_O_3_@Zn-LDH-EAE-SO_3_H (IV) were studied by VSM measurements (Fig. 8(a and b)). According to the results presented in Fig. 8, the saturation magnetic moment of γ-Fe_2_O_3_@Zn-LDH-EAE-SO_3_H (IV) was measured at *M*_s_ = 24.80 emu g^−1^, which is lower than that of the γ-Fe_2_O_3_@Zn-LDH (II) (*M*_s_ = 10.85 emu g^−1^). The reduction in saturation magnetization of γ-Fe_2_O_3_@Zn-LDH-EAE-SO_3_H (IV) can be ascribed to the influence of non-magnetic materials grafted on the surface of γ-Fe_2_O_3_@Zn-LDH (II).

### 3.2 Catalytic performance of γ-Fe_2_O_3_@Zn-LDH-EAE-SO_3_H (IV) in the synthesis of chromeno[4,3-*b*]quinoline-6,8-dione derivatives

**Scheme 2:**
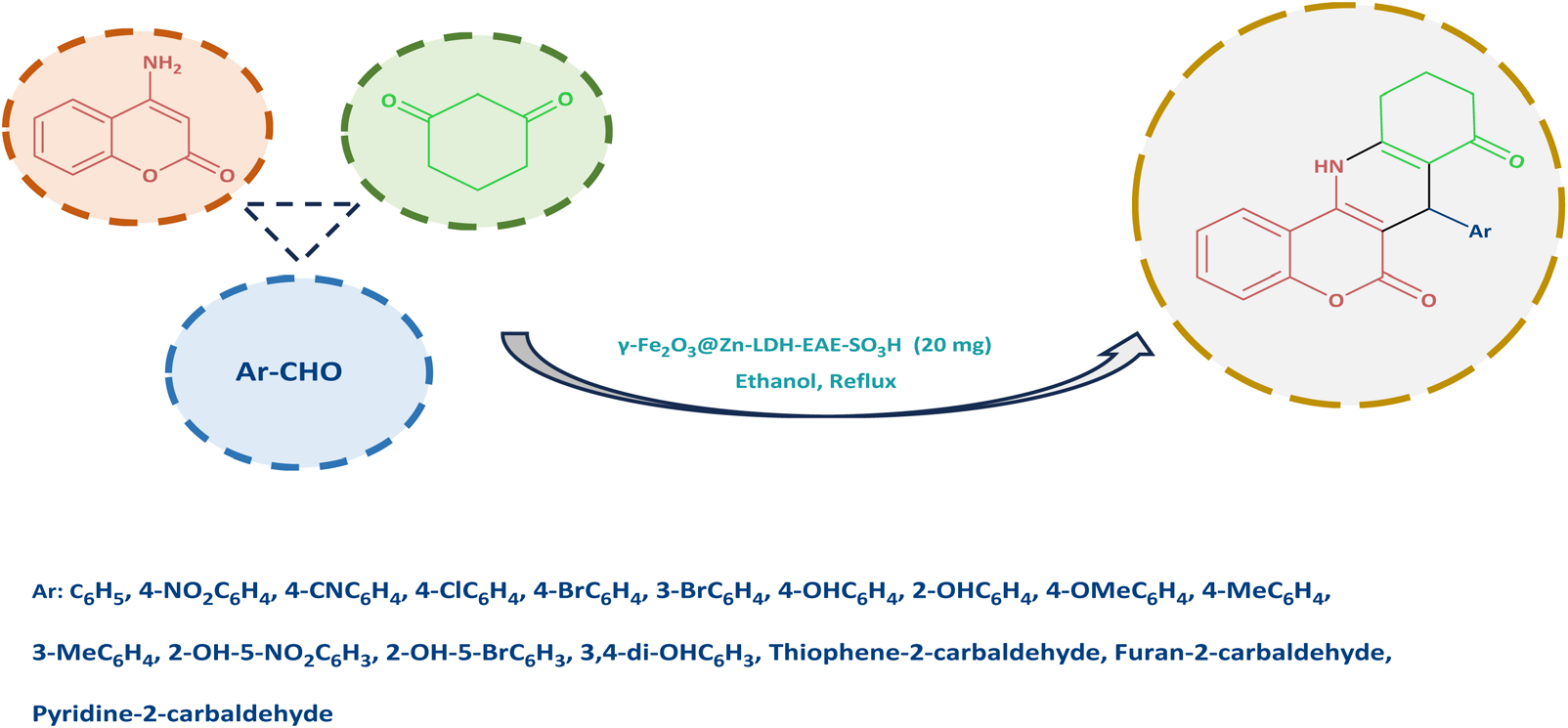
Synthesis of chromeno[4,3-*b*]quinoline derivatives in the presence of γ-Fe_2_O_3_@Zn-LDH-EAE-SO_3_H (IV).

After full characterization of γ-Fe_2_O_3_@Zn-LDH-EAE-SO_3_H (IV), its catalytic activity was investigated towards the synthesis of chromeno[4,3-*b*]quinoline derivatives (Scheme 2). At first, we examined the reaction between 4-aminocoumarin, benzaldehyde, and 1,3-cyclohexanedione in a molar ratio of 1/1/1 as a model reaction. Subsequently, different factors such as catalyst loading, temperature and solvents were evaluated to determine the optimal reaction conditions (Table 1). In the initial attempts of investigation, based on the fundamental principle of green chemistry, EtOH was chosen as the solvent instead of hazardous solvents during the optimization reactions.^96^ No desired product was obtained, when the model reaction was performed in the absence of catalyst even after a long period of time (240 min) (Table 1, entry 1). In a set of experiments, a range of catalyst loadings were assessed to determine their impact on the rate and yield of the model reaction (Table 1, entries 2–5). It was clear from Table 1 that using 20 mg of the catalyst resulted in the highest yield of the desired product in a short reaction time. Increasing the catalyst amount did not result in a significant increase in product yields, while reducing the catalyst amount resulted in a lower yield. It is evident from Table 1, that the reaction showed strong dependence on temperature (Table 1, entries 6–8). Temperature tests revealed that refluxing ethanol was the optimal reaction temperature. In addition, the effect of various solvents such as MeOH, H_2_O, H_2_O : EtOH (1 : 1), PEG, and CH_3_CN and solvent-free conditions was examined on the model reaction (Table 1, entries 9–15). It was found that the reaction proceeded more efficiently in EtOH compared to the other solvents used. To gain a better understanding of the role of the nanostructured catalyst in the synthesis of 7-phenyl-7,10,11,12-tetrahydro-6H-chromeno[4,3-*b*]quinoline-6,8(9*H*)-dione, the model reaction was performed in the presence of γ-Fe_2_O_3_ NPs (I), γ-Fe_2_O_3_@Zn-LDH (II), and γ-Fe_2_O_3_@Zn-LDH-E (III) as well as Mg(NO_3_)_2_·6H_2_O, Al(NO_3_)_3_·9H_2_O, Zn(NO_3_)_2_·6H_2_O and Zn(NO_3_)_2_·6H_2_O : Mg(NO_3_)_2·_6H_2_O : Al(NO_3_)_3_·9H_2_O (Table 1 entries 16–22). Interestingly, in the presence of γ-Fe_2_O_3_@Zn-LDH (II) and γ-Fe_2_O_3_@Zn-LDH-E (III) as catalysts, a lower yield of reaction was obtained. However, in other cases after a prolonged reaction time, the yield of the target compound was not satisfactory. The best result was achieved when the reaction was carried out in the presence of γ-Fe_2_O_3_@Zn-LDH-EAE-SO_3_H (IV) (20 mg) in refluxing ethanol.

**Table 1:** Optimization of various reaction parameters in the synthesis of 7-phenyl-7,10,11,12-tetrahydro-6*H*-chromeno[4,3-*b*]quinoline-6,8(9*H*)-dione

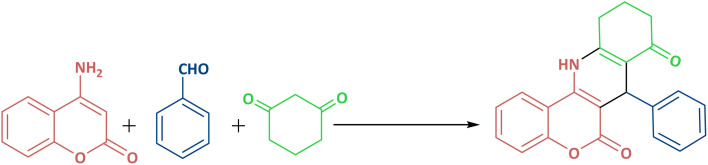					
Entry	Catalyst (mg)	Solvent	Temperature (°C)	Time (min)	Isolated yield (%)
1	—	EtOH	Reflux	240	—
2	10	EtOH	Reflux	30	80
3	15	EtOH	Reflux	20	90
**4**	**20**	**EtOH**	**Reflux**	**15**	**95**
5	25	EtOH	Reflux	15	95
6	20	EtOH	60	30	60
7	20	EtOH	65	25	75
8	20	EtOH	70	20	90
9	20	MeOH	Reflux	35	70
10	20	H_2_O	Reflux	35	50
11	20	H_2_O/EtOH (1 : 1)	Reflux	35	65
12	20	Solvent free	100	40	40
13	20	PEG-400	120	50	40
14	20	CH_3_CN	Reflux	60	35
15	20	EtOAc	Reflux	60	30
16^a^	20	EtOH	Reflux	240	Trace
17^b^	20	EtOH	Reflux	30	20
18^c^	20	EtOH	Reflux	30	25
19^d^	1.92 g	EtOH	Reflux	240	Trace
20^e^	1.12 g	EtOH	Reflux	240	Trace
21^f^	0.44 g	EtOH	Reflux	240	Trace
22^g^	1.92 : 1.12 : 0.44 g	EtOH	Reflux	240	Trace

a The reaction was performed in the presence of γ-Fe_2_O_3_ NPs (I).

b The reaction was performed in the presence of γ-Fe_2_O_3_@Zn-LDH (II) NPs.

c The reaction was performed in the presence of γ-Fe_2_O_3_@Zn-LDH-E (III) NPs.

d The reaction was performed in the presence of Mg(NO_3_)_2_·6H_2_O.

e The reaction was performed in the presence of Al(NO_3_)_3_·9H_2_O.

f The reaction was performed in the presence of Zn(NO_3_)_2_·6H_2_O.

g The reaction was performed in the presence of Zn(NO_3_)_2_·6H_2_O : Mg(NO_3_)_2_·6H_2_O : Al(NO_3_)_3_·9H_2_O.

**Scheme 3:**
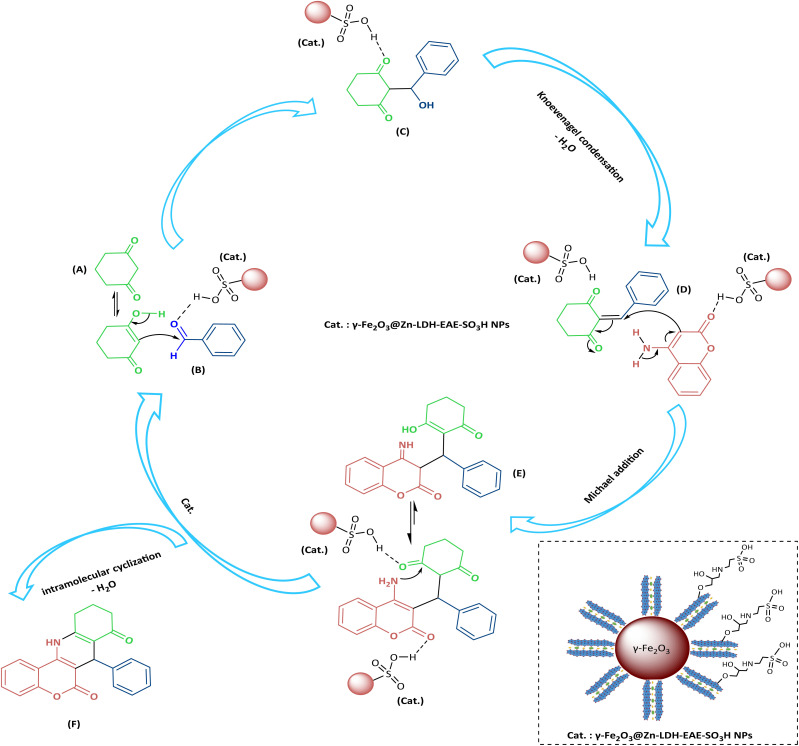
Proposed reaction mechanism for the preparation of chromeno[4,3-*b*]quinoline-6,8-dione derivatives using γ-Fe_2_O_3_@Zn-LDH-EAE-SO_3_H (IV).

To expand the current method for the synthesis of chromeno[4,3-*b*]quinoline-6,8-dione derivatives, a range of aryl aldehydes were reacted with 4-aminocoumarin and 1,3-cyclohexanedione in the presence of γ-Fe_2_O_3_@Zn-LDH-EAE-SO_3_H (IV) under the optimal reaction conditions (Table 2). Aromatic aldehydes containing both electron-donating and electron-withdrawing groups result in high to excellent yields of the desired products. The reaction time was affected by the type of substituents present on the aryl aldehydes. Aldehydes involving electron-withdrawing substituents (4-nitrobenzaldehyde, 4-cyanobenzaldehyde, 4-chlorobenzaldehyde and 4-bromobenzaldehyde) reacted in short reaction times (Table 2, entries 2–6). In contrast, aldehydes bearing electron-donating substituents (4-hydroxybenzaldehyde, 2-hydroxybenzaldehyde, 4-methoxybenzaldehyde, 4-methylbenzaldehyde and 3-methylbenzaldehyde) require longer reaction times (Table 2, entries 7–11). In comparison, the *para*-substituted aryl aldehydes provided better results than the *meta*- and *ortho*-substituted ones (Table 2 entry 5 *vs.* 6, entry 7 *vs.* 8, entry 10 *vs.* 11), due to the electronic effect in *para*-position and the steric hindrance in *ortho-*position. The current catalytic system also showed good performance with heteroaromatic aldehydes such as thiophene-2 carbaldehyde and furan-2-carbaldehyde (Table 2 entries 15, 16). Unfortunately, a trace amount of expected product was formed in the case of pyridine-2-carbaldehyde even after long reaction times due to the electronic effects on 2-, 4- and 6-positions of the pyridine ring. Low electron density on 2-position led to the weak interaction of the carbonyl group with acidic –SO_3_H sites *via* hydrogen bonding as the essential factor, which proceeds the reaction (based on the proposed mechanism in Scheme 3).

**Table 2:** One-pot synthesis of chromeno[4,3-*b*]quinoline-6,8-dione derivatives in the presence of γ-Fe_2_O_3_@Zn-LDH-EAE-SO_3_H(IV)

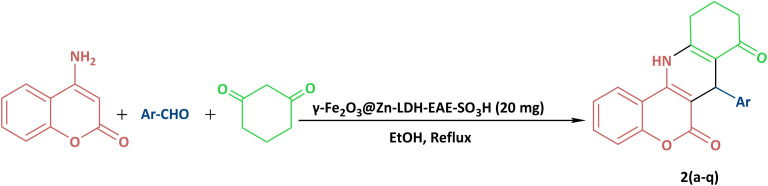				
Entry	Ar	Product	Time (min)	Isolated yield (%)
1	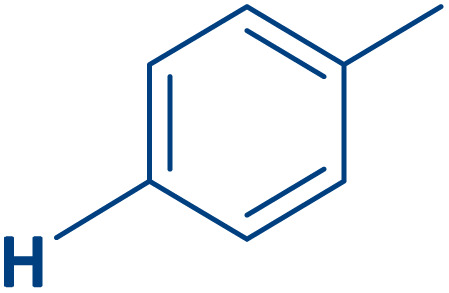	2a	15	95
2	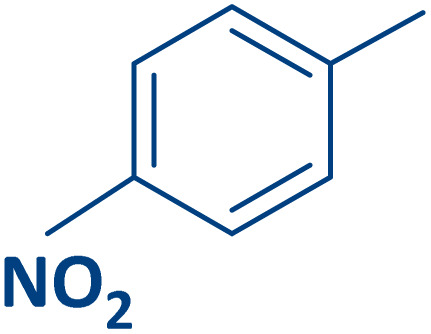	2b	15	95
3	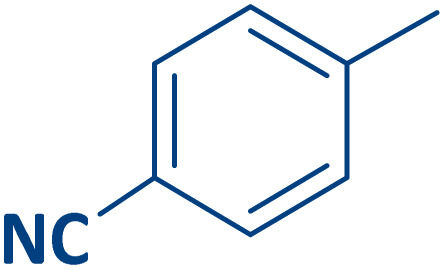	2c	20	95
4	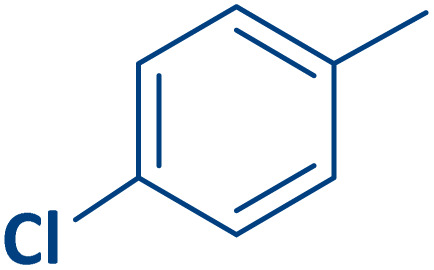	2d	30	85
5	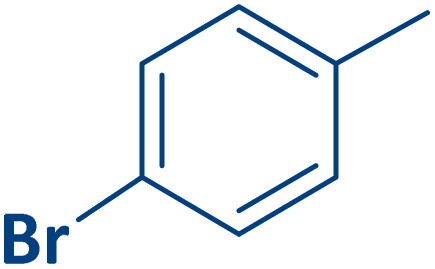	2e	30	80
6	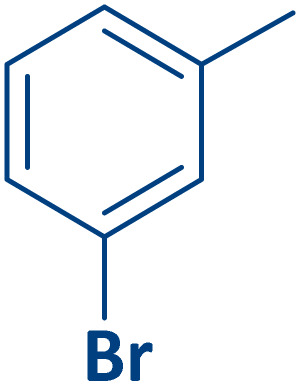	2f	35	80
7	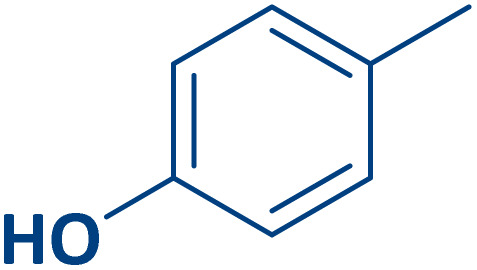	2g	35	80
8	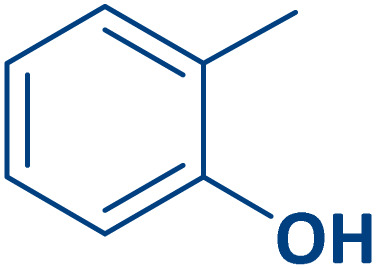	2h	40	80
9	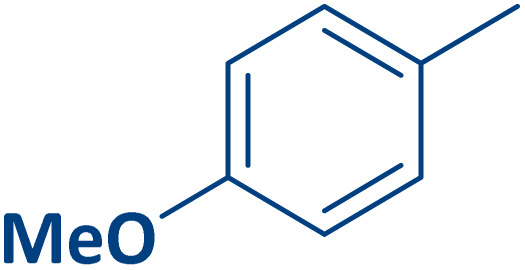	2i	35	80
10	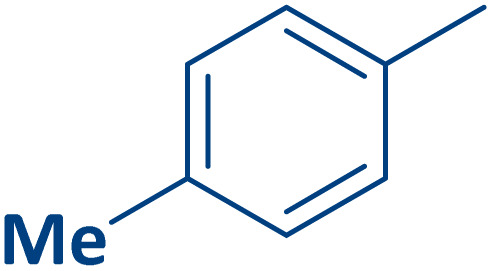	2j	40	80
11	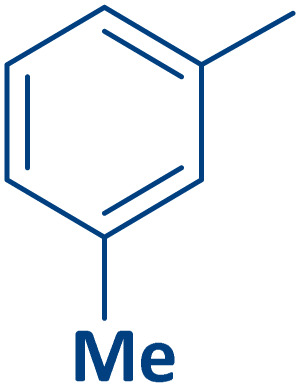	2k	45	75
12	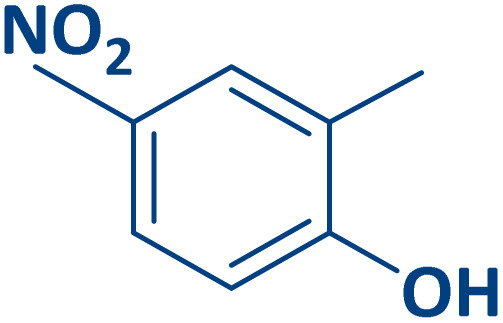	2l	45	85
13	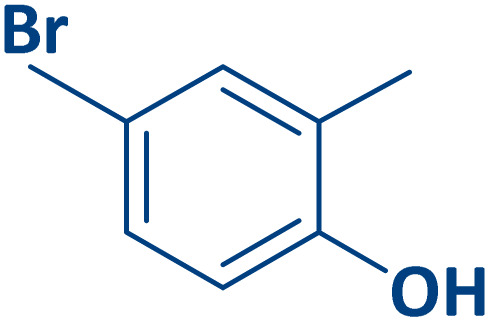	2m	50	80
14	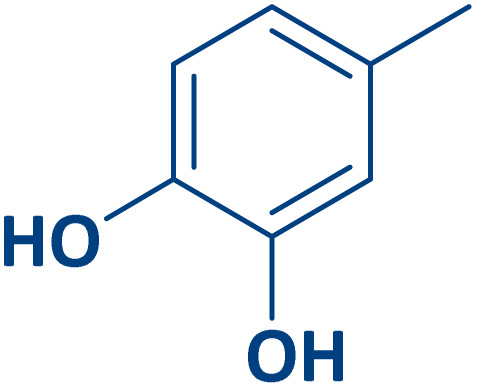	2n	55	70
15	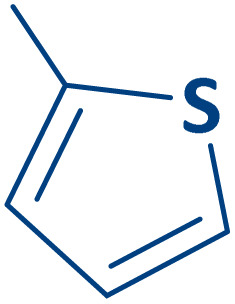	2o	30	80
16	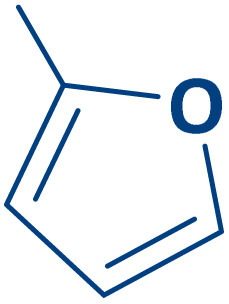	2p	35	80
17	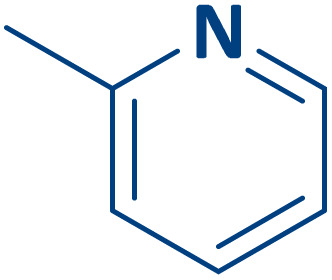	2q	240	Trace

The progress of the reaction was periodically monitored by observing the disappearance of the aldehydes and the subsequent formation of the desired products on TLC. The structures of the products were confirmed by recording melting points, mass spectrometry, FT-IR spectroscopy, ^1^H NMR spectroscopy and ^13^C NMR spectroscopy. Additionally, the novel synthesized compounds (2c, 2l, 2m and 2n) were characterized using elemental analysis data. FT-IR spectra of the synthesized compounds displayed sharp absorption bands around 3350–3308 cm^−1^ related to the NH stretching vibration. The stretching frequencies assigned to = C–H and -C-H appeared around 3096–3035 cm^−1^ and 2966–2872 cm^−1^, respectively. Furthermore, the presence of the key absorption band around 1728–1668 cm^−1^ corresponded to the stretching vibration of the carbonyl group, confirming the formation of the desired products. The ^1^H NMR spectra displayed a resonating singlet signal between 9.86 and 9.68 ppm, assigned to the NH proton of the quinoline ring. Besides, a sharp singlet signal in the range of 5.18–4.91 ppm confirmed the presence of a methine (CH) group at 7-position. However, in the ^13^C NMR spectra of all products, the carbonyl carbon appeared at a lower chemical shift (195.89–195.33 ppm and 160.90–160.70 ppm) compared to the typical range of unconjugated C=O groups (220–200 ppm).^97^ The signal at 37.27–36.98 ppm is associated with the C–C bond formation during the ring-closure reaction (see ESI file†).

#### 3.2.1 Spectral and physical data of 7-phenyl-7,10,11,12-tetrahydro-*6H*-chromeno[4,3-*b*]quinoline-6,8(*9H*)-dione (2a)^33^

Light yellow solid; (0.325 g, 95%); mp = 324–325 °C (Lit. 324–326 °C); IR (KBr) (*υ*_max_/cm^−1^): 3338 (NH), 3092, 3047 (C–H aromatic), 2953, 2876 (C–H aliphatic), 1703 (C=O), 1632, 1605 (C=C); ^1^H NMR (300 MHz, DMSO-*d*_*6*_): *δ* (ppm): 9.78 (s, 1H, NH), 8.34 (d, *J* = 8.1 Hz, 1H, ArH, H_1_), 7.66 (t, *J* = 7.8 Hz, 1H, ArH, H_3_), 7.46 (t, *J* = 7.6 Hz, 1H, ArH, H_2_), 7.40 (d, *J* = 8.3 Hz, 1H, ArH, H_4_), 7.28–7.19 (m, 4H, ArH, H_14_, H_15_, H_17_, H_18_), 7.12 (t, *J* = 5.9 Hz, 1H, ArH, H_16_), 5.02 (s, 1H, CH, H_7_), 2.91–2.83 (m, 1H, CH_2_, H_9_), 2.78–2.66 (m, 1H, CH_2_, H_9_), 2.36–2.25 (m, 2H, CH_2_, H_11_), 2.07–1.97 (m, 1H, CH_2_, H_10_), 1.95–1.85 (m, 1H, CH_2_, H_10_); ^13^C NMR (75 MHz, DMSO-*d*_*6*_): *δ* (ppm): 195.45 (C_8_), 160.84 (C_6_), 152.52 (C_4a_), 152.17 (C_11a_), 146.41 (C_12a_), 142.55 (C_13_), 132.44 (C_3_), 128.53 (C_17_, C_15_), 128.17 (C_18_, C_14_), 126.69 (C_16_), 124.49 (C_1_), 123.45 (C_2_), 117.36 (C_4_), 113.52 (C_12b_), 112.36 (C_7a_), 102.28 (C_6a_), 37.16 (C_9_), 34.63 (C_7_), 26.87 (C_11_), 21.22 (C_10_); MS: (*m*/*z*, %): 343 (M^+^, 10), 341 (M^+^-2, 70), 266 (68), 210 (10), 76 (10), 28 (100); anal. calcd. for C_22_H_17_NO_3_ (343): C: 76.95, H: 4.99, N: 4.08%. Found: C: 76.88, H: 4.91, N: 3.99%.

### 3.3 Proposed catalytic mechanism for the synthesis of chromeno[4,3-*b*]quinoline-6,8-dione derivatives

Based on the studies reported in the literature,^44^ the probable reaction mechanism for the synthesis of chromeno[4,3-*b*]quinoline-6,8-dione derivatives using γ-Fe_2_O_3_@Zn-LDH-EAE-SO_3_H (IV) is illustrated in Scheme 3. Based on the obtained results (Table 1, entries 1 and 16–22), when the model reaction was carried out in the absence of catalysts as well in the presence of γ-Fe_2_O_3_ (I), γ-Fe_2_O_3_@Zn-LDH (II), γ-Fe_2_O_3_@Zn-LDH-E (III), and metal salts, no satisfactory yield of the desired product was obtained. Therefore, it can be concluded that γ-Fe_2_O_3_@Zn-LDH-EAE-SO_3_H (IV) significantly affects each step of chromeno[4,3-*b*]quinoline-6,8-dione synthesis as a magnetic nanostructured catalyst.

Initially, intermediate C was formed upon the nucleophilic attack of the enolic form of 1,3-cyclohexanedione A to activate the carbonyl group of aldehyde B (*via* hydrogen bonding between the oxygen atom of the carbonyl group and acidic –SO_3_H sites on the surface of the nanostructured catalyst). Then, D as a strong Michael acceptor was obtained after dehydration of C (Knoevenagel condensation). In the next step, E was produced through Michael addition reaction of 4-aminocoumarin (activated by acidic –SO_3_H sites on the surface of the nanostructured catalyst) to D. Finally, an intramolecular cyclization reaction (through amino group attacks to the activated carbonyl group by catalyst) and subsequent elimination of the water molecule led to the formation of desired product F alongside the regeneration of the catalyst.

### 3.4 Reusability of γ-Fe_2_O_3_@Zn-LDH-EAE-SO_3_H (IV)

**Fig. 9:**
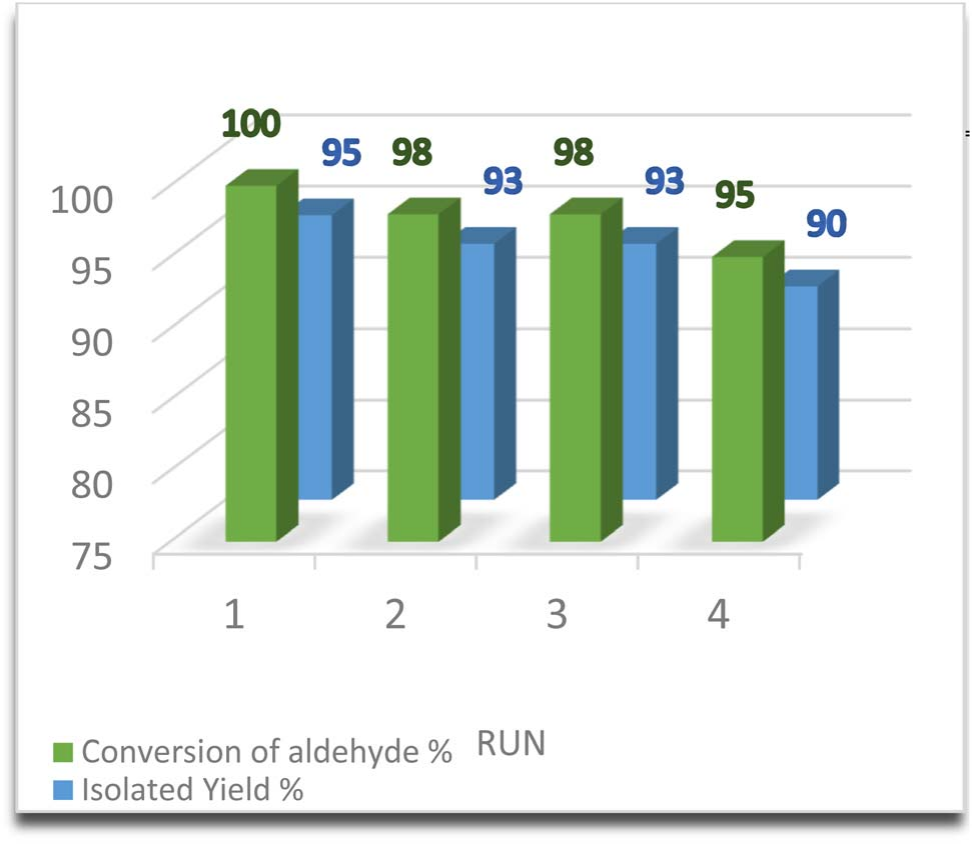
Synthesis of 7-phenyl-7,10,11,12-tetrahydro-6*H*-chromeno[4,3-*b*]quinoline-6,8(9*H*)-dione in the presence of the recovered γ-Fe_2_O_3_@Zn-LDH-EAE-SO_3_H(IV).

The reusability of the catalyst is a crucial factor, particularly for commercial applications, as it helps to reduce process costs. To align with green chemistry principles, the recovery and reusability of γ-Fe_2_O_3_@Zn-LDH-EAE-SO_3_H (IV) as a magnetic nanostructured catalyst were investigated on the model reaction under optimized conditions. Upon completion of the reaction, the catalyst was easily recovered from the reaction mixture using a magnetic field. Subsequently, the recovered catalyst was washed with hot ethyl acetate before drying at 60 °C for 2 h and reused for up to four reaction cycles. The results show that the recycled and reused catalysts have the same activity as the fresh catalyst, with only a slight decrease in product yield. The isolated yields of the products are 95%, 93%, 93%, and 90% after successive cycles (Fig. 9). It is evident from the FT-IR spectrum of the 4th recovered γ-Fe_2_O_3_@Zn-LDH-EAE-SO_3_H(IV) that there were no notable changes in the shape or intensity of the characteristic absorption bands (Fig. 1(d) *vs.* (e)). Additionally, back-titration of the fourth recovered γ-Fe_2_O_3_@Zn-LDH-EAE-SO_3_H (IV) showed a slight decrease in the number of acidic sites (sulfonic groups) to 0.7 mmol per 1.000 g of the nanostructured catalyst. The results indicate minimal leaching (12.5%) of the active acidic sites during four catalytic reaction cycles (the fresh catalyst contains 0.8 mmol of acidic groups per 1.000 g). Therefore, the catalytic performance of the fresh nanostructured catalyst remains nearly the same as the recovered one even after four reaction cycles.

To demonstrate the significance of the current method for the synthesis of chromeno[4,3-*b*]quinoline-6,8-dione, we compared the results of the model reaction in the presence of γ-Fe_2_O_3_@Zn-LDH-EAE-SO_3_H (IV) with those obtained using other catalysts published recently, as shown in Table 3. Using high reaction temperatures, large amounts of catalyst, use of microwave irradiation (Table 3, entries 1), harmful solvents and long reaction times (Table 3, entries 2 and 3) to achieve the desired product are the drawbacks of some of these methods. Thus, this comparison obviously indicates that γ-Fe_2_O_3_@Zn-LDH-EAE-SO_3_H (IV) as a green and heterogeneous nanostructured catalyst is more efficient than the other methods regarding the product yield and reaction time.

**Table 3:** Comparison of the efficiency of γ-Fe_2_O_3_@Zn-LDH EAE-SO_3_H (IV) with those of other catalysts for the synthesis of 7-phenyl-7,10,11,12-tetrahydro-6*H*-chromeno[4,3-*b*]quinoline-6,8(9*H*)-dione

Entry	Catalyst	Solvent	Temperature (°C)	Time (min)	Yield (%)	Ref.
1	H_3_PW_12_O_40_^a^ (28.8 mg)	—	100	5	85	98
3	—	Acetic acid	110	2–3 (h)	70	97
4	—	Acetic acid	110	2–3 (h)	92	33
5	γ-Fe_2_O_3_@Zn-LDH@EAE-SO_3_H (20 mg)	EtOH	Reflux	15	95	Present study

a Microwave irradiation.

## 4. Conclusion

In summary, a convenient and eco-friendly method for the synthesis of chromeno[4,3-*b*]quinoline-6,8-dione derivatives using γ-Fe_2_O_3_@Zn-LDH@EAE-SO_3_H (IV) as the catalyst was reported. γ-Fe_2_O_3_@Zn-LDH@EAE-SO_3_H (IV) as a novel heterogeneous nanostructured catalyst was prepared and characterized by FT-IR spectroscopy, XRD, TEM, FE-SEM, EDS, TGA, CHNS and VSM techniques. The characterization results confirmed the spherical shape of γ-Fe_2_O_3_@Zn-LDH@EAE-SO_3_H (IV) with an average diameter ranging from 5 to 17 nm. There are a myriad of advantages in this method including mild reaction conditions, enhanced reaction rate, excellent yield of the products and elimination of harmful solvents, which make this method an excellent alternative to other previous approaches for synthesizing this category of compounds in organic chemistry. Moreover, functionalization of the nanostructured catalyst by taurine as a bifunctional organic molecule that has both sulfonic acid (–SO_3_H) and amino (–NH_2_) groups alongside four times recycling without any significant loss of activity is beneficial for biocompatibility and non-toxicity. 2-Aminoethanesulfonic acid (taurine) as a ''conditionally essential'' amino acid was found throughout the body, particularly in the brain, eyes, heart and muscles.^99^ Body can produce some amount of taurine, and it is also found in some foods such as meat, fish and dairy^100^ Furthermore, using ethanol as a renewable and environmentally benign solvent makes this method an excellent alternative to other previous approaches for synthesizing this category of compounds in organic chemistry.

## Author contributions

Ahad Vatandoust Namanloo: experimental investigation, visualization and writing. Batool Akhlaghinia: supervision, writing, review, editing and funding acquisition.

## Conflicts of interest

The authors declare no conflict of interest.

## Supplementary Material

RA-015-D5RA03659C-s001

RA-015-D5RA03659C-s002

## Data Availability

All the data generated or analyzed during this study, such as FT-IR spectroscopy, XRD, TEM, FE-SEM, EDX, EDX-mapping, TGA, VSM, NMR and CHNS results, are included in the manuscript.
