# Correction: γ-Fe_2_O_3_@Zn-LDH-EAE-SO_3_H for multi-component synthesis of chromeno[4,3-*b*]quinoline-6,8-dione derivatives

**DOI:** 10.1039/d5ra90085a

**Published:** 2025-07-11

**Authors:** Ahad Vatandoust Namanloo, Batool Akhlaghinia

**Affiliations:** a Department of Chemistry, Faculty of Science, Ferdowsi University of Mashhad Mashhad 9177948974 Iran akhlaghinia@um.ac.ir

## Abstract

Correction for ‘γ-Fe_2_O_3_@Zn-LDH-EAE-SO_3_H for multi-component synthesis of chromeno[4,3-*b*]quinoline-6,8-dione derivatives’ by Ahad Vatandoust Namanloo *et al.*, *RSC Adv.*, 2025, **15**, 21465–21478, https://doi.org/10.1039/D5RA03659C.

The authors regret an error in the spelling of Dr Batool Akhlaghinia's name in the original article. The correct spelling is as shown in this Correction article.

The Royal Society of Chemistry apologises for these errors and any consequent inconvenience to authors and readers.

