# Supplementary material for: γ-Fe2O3@Zn-LDH-EAE-SO3H for multi-component synthesis of chromeno[4,3-b]quinoline-6,8-dione derivatives
Source: RSC Adv. 2025 Jun 24;15(27):21465–78. doi: 10.1039/d5ra03659c (PMC12186498; doi:10.1039/d5ra03659c)
Supplement: RA-015-D5RA03659C-s002 [file RA-015-D5RA03659C-s002.pdf]

Supplementary Information (SI) for RSC Advances.
This journal is © The Royal Society of Chemistry 2025

Supplementary Information

γ-Fe_2O_3@Zn-LDH-EAE-SO_3H for multi-component synthesis of chromeno[4,3-*b*]quinoline-6,8-dione derivatives

Ahad Vatandoust Namanloo and Batool Akhlaghnia*

Department of Chemistry, Faculty of Science, Ferdowsi University of Mashhad, Mashhad 9177948974, Iran

*E-mail: akhlaghinia@um.ac.ir

E-mail: vatandoust.ahad@gmail.com

Table of Contents

General Information……………………………………………………………….……….. **2**

Spectra and physical data of 2(a-p) ……………………………………….……............ **3-85**

References………………………………………………………..……...……...……....... **86**

General information

The purity determinations of the products and the progress of the reactions were accomplished by TLC on silica gel polygram STL G/UV 254 plates. The melting points of the products were determined with an Electrothermal Type 9100 melting point apparatusThe FT-IR spectra were recorded on pressed KBr pellets using an AVATAR 370 FT-IR spectrometer (Therma Nicolet spectrometer, USA) at room temperature in the range between 4000 and 400 cm^{-1} with a resolution of 4 cm^{-1}. The NMR spectra were obtained in Brucker Avance 300 MHz instruments in DMSO-d_6 as solvent. Mass spectra were determined at 70 eV on a CH7A Varianmat Bremem instrument elemental analyses were performed using a Thermo Finnigan Flash EA 1112 Series instrument. All yields refer to isolated products after purification by column chromatography.

Spectral and physical data of 2(a-p)

7-phenyl-7,10,11,12-tetrahydro-*6H*-chromeno[4,3-*b*]quinoline-6,8(*9H*)-dione (2a)[1]

Light yellow solid; (0.325g, 95%); Mp=324-325 °C (Lit. 324-326 °C); IR (KBr) (υ_{max}/cm^{-1}): 3338 (NH), 3092, 3047 (C-H aromatic), 2953, 2876 (C-H aliphatic), 1703(C=O), 1632, 1605 (C=C); ^{1}H NMR (300 MHz, DMSO-d_6): δ (ppm): 9.78 (s, 1H, NH), 8.34 (d, J = 8.1 Hz, 1H, ArH, H_1), 7.66 (t, J = 7.8 Hz, 1H, ArH, H_3), 7.46 (t, J = 7.6 Hz, 1H, ArH, H_2), 7.40 (d, J = 8.3 Hz, 1H, ArH, H_4), 7.28-7.19 (m, 4H, ArH, H_{14}, H_{15}, H_{17}, H_{18}), 7.12 (t, J = 5.9 Hz, 1H, ArH, H_{16}), 5.02 (s, 1H, CH, H_7), 2.91-2.83 (m, 1H, CH_2, H_9), 2.78-2.66 (m, 1H, CH_2, H_9), 2.36-2.25 (m, 2H, CH_2, H_{11}), 2.07-1.97 (m, 1H, CH_2, H_{10}), 1.95-1.85 (m, 1H, CH_2, H_{10}); ^{13}C NMR (75 MHz, DMSO-d_6): δ (ppm): 195.45 (C_8), 160.84 (C_6), 152.52 (C_{4a}), 152.17 (C_{11a}), 146.41 (C_{12a}), 142.55 (C_{13}), 132.44 (C_3), 128.53 (C_{17},C_{15}), 128.17 (C_{18},C_{14}), 126.69 (C_{16}), 124.49 (C_1), 123.45 (C_2), 117.36 (C_4), 113.52 (C_{12b}), 112.36 (C_{7a}), 102.28 (C_{6a}), 37.16 (C_9), 34.63 (C_7), 26.87 (C_{11}), 21.22 (C_{10}); MS: (m/z, %): 343 (M^+, 10), 341 (M^+-2, 70), 266 (68), 210 (10), 76 (10), 28 (100); Anal. Calcd. for $C_{22}H_{17}NO_3$ (343): C: 76.95, H: 4.99, N: 4.08%. Found: C: 76.88, H: 4.91, N: 3.99%.

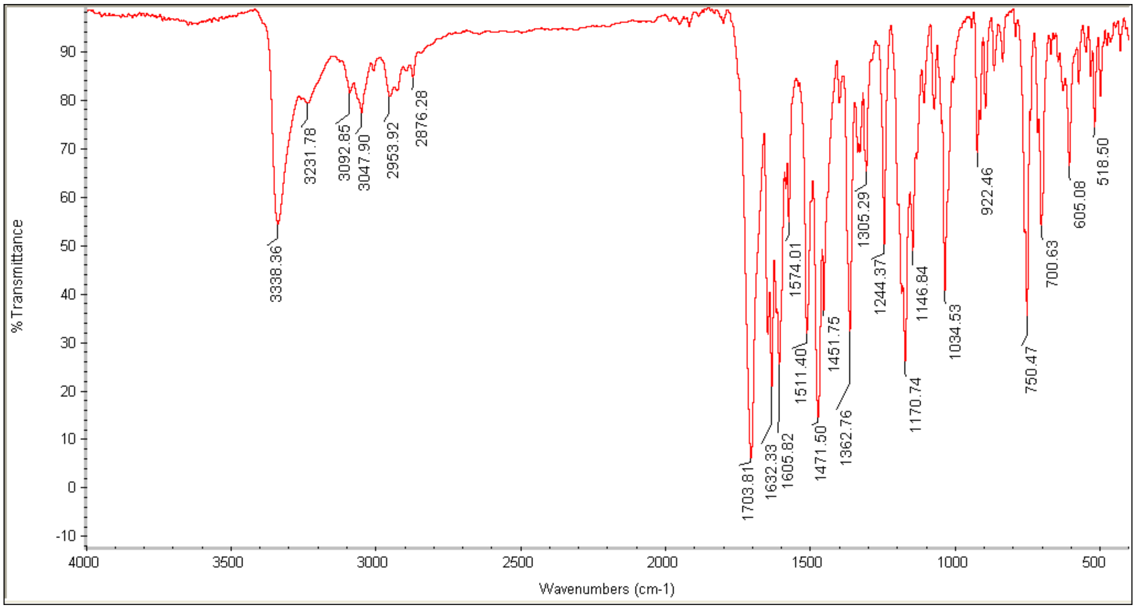

Figure 1. IR spectrum of compound **2a**

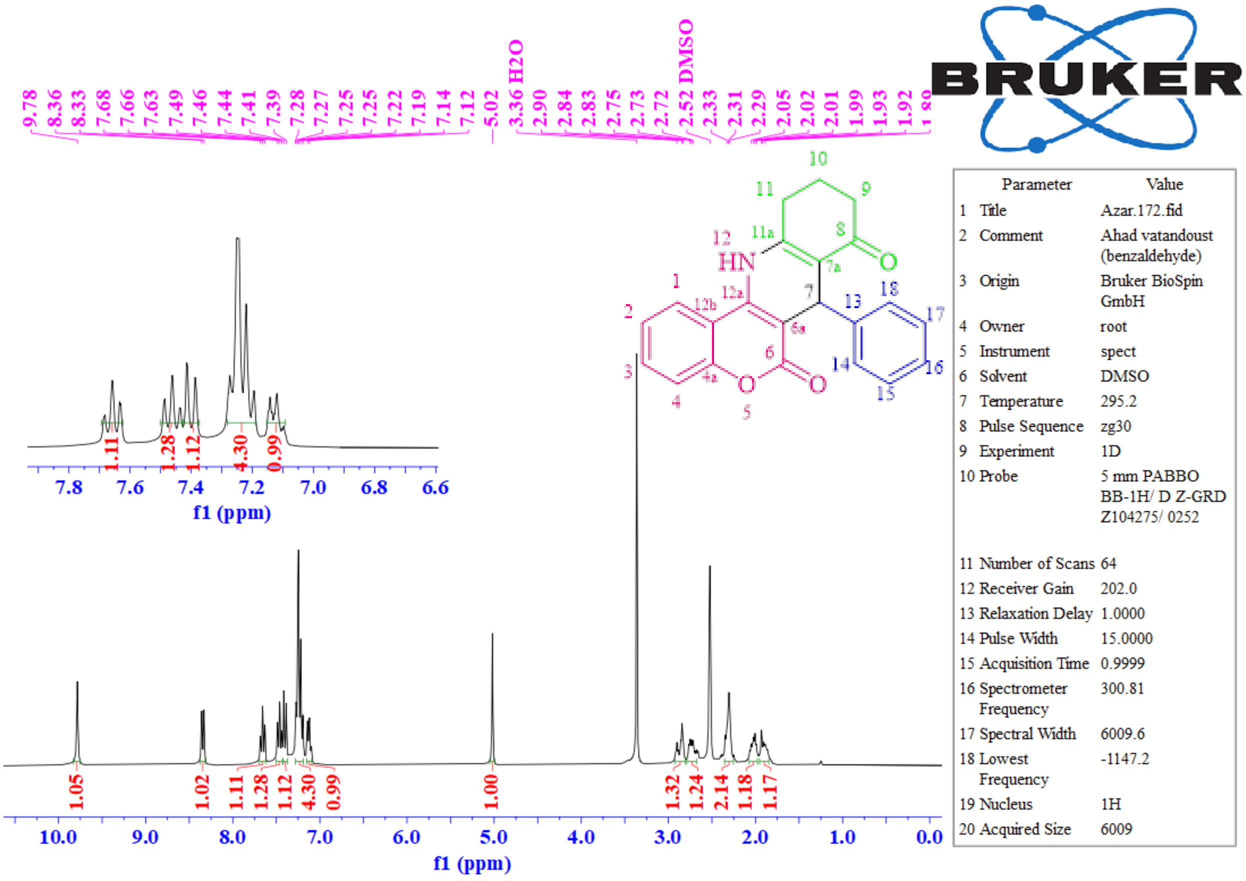

Figure 2. ^{1}H NMR (300 MHz, DMSO-d_6) spectrum of compound **2a**

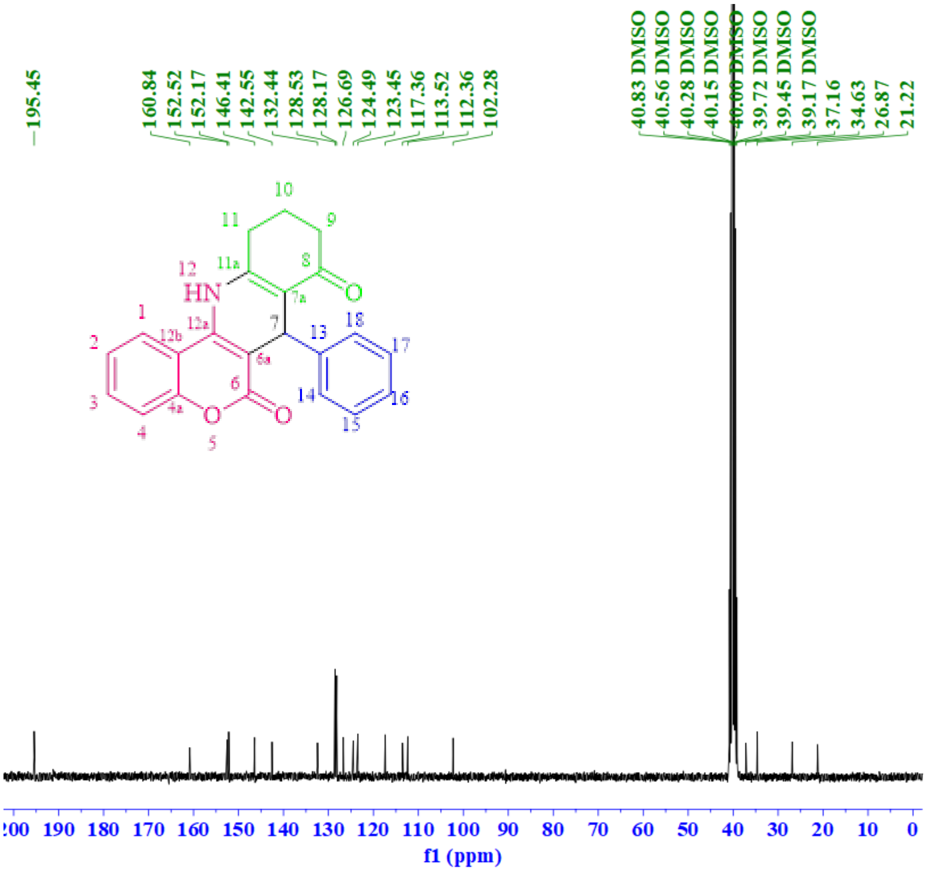

Parameter		Value
1	Title	Azar.155.fid
2	Comment	Ahad vatandoust (benzaldehyde)
3	Origin	Bruker BioSpin GmbH
4	Owner	root
5	Instrument	spect
6	Solvent	DMSO
7	Temperature	296.7
8	Pulse Sequence	zgpg30
9	Experiment	1D
10	Probe	5 mm PABBO BB-1H/ D Z-GRD Z104275/ 0252
11	Number of Scans	1594
12	Receiver Gain	202.0
13	Relaxation Delay	2.0000
14	Pulse Width	10.0000
15	Acquisition Time	1.8088
16	Spectrometer Frequency	75.65
17	Spectral Width	18115.9
18	Lowest Frequency	-1494.8
19	Nucleus	13C
20	Acquired Size	32768

Figure 3. ^{13}C NMR (75 MHz, DMSO-d_6) spectrum of compound **2a**

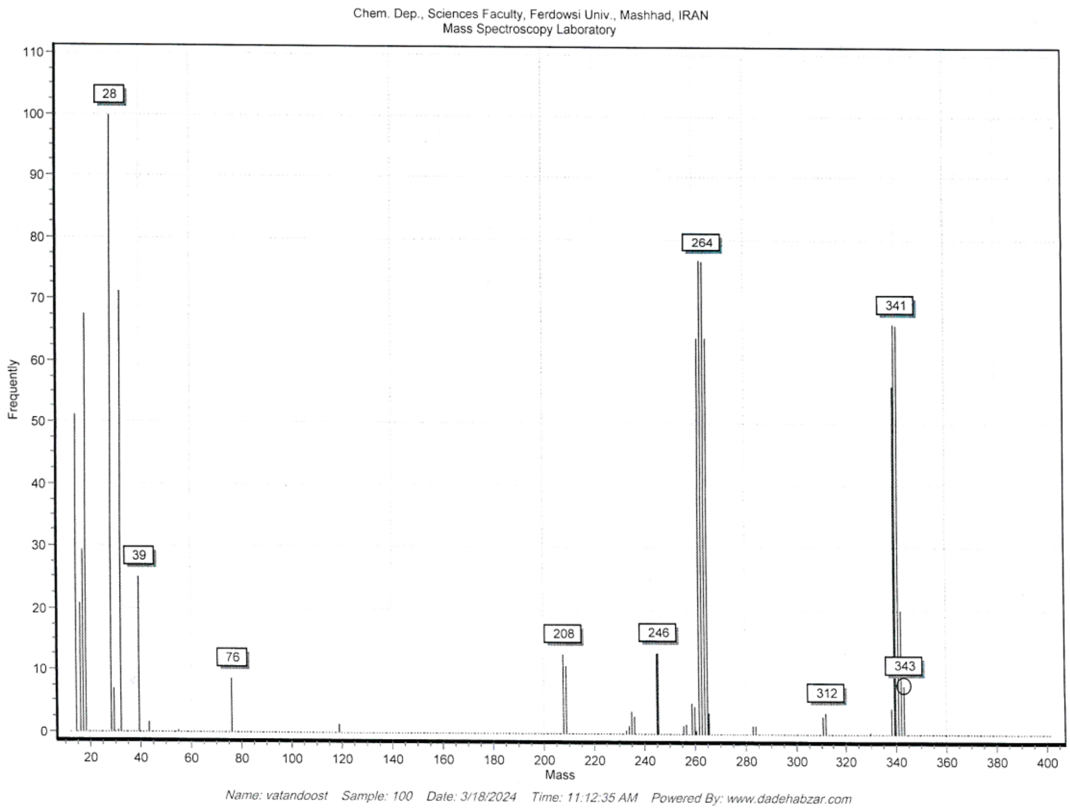

Figure 4. Mass spectrum of compound **2a**

Eager 300 Summarize Results

Date: 24/04/2024 at 13:07:30

Method Name: NCHS

Method Filename: Copy of N C H S-bkp.mth

Filename		As Method				Vial
Vatandoust-187						
# Group Sample Name		Tayp Weig. Prof. F			---	-----
187-1	100	UNK	0.622	6.25	---	-----

Component Name	Element%
Nitrogen%	3.995759434
Carbon%	76.88702856
Hydrogen%	4.917127304
Sulphur%	0

1 Sample (s) in Group No:1

Component Name	Average
Nitrogen%	3.995759434
Carbon%	76.88702856
Hydrogen%	4.917127304
Sulphur%	0

Figure 5. CHNS spectrum of compound **2a**

Anal. Calcd. for $C_{22}H_{17}NO_3$ (343)		
C: 76.95 %	H: 4.99 %	N: 4.08 %

7-(4-nitrophenyl)-7,10,11,12-tetrahydro-6H-chromeno[4,3-b]quinoline-6,8(9H)-dione (2b)[2]

Light yellow solid; (0.368g, 95%); Mp=259-261 °C (Lit. 260-262 °C) ; IR (KBr) (υ_{max}/cm^{-1}): 3321 (NH), 3092 (C-H aromatic), 2942, 2884 (C-H aliphatic), 1671 (C=O), 1645, 1609 (C=C); ^{1}H NMR (300 MHz, DMSO-d_6): δ (ppm): 9.88 (s, 1H, NH), 8.35 (d, J = 8.1 Hz, 1H, ArH, H_1), 8.10 (d, J = 8.6 Hz, 2H, ArH, H_{15}, H_{17}), 7.66 (t, J = 7.8 Hz, 1H, ArH, H_3), 7.54 (d, J = 8.5 Hz, 2H, ArH, H_{14}, H_{18}), 7.46 (t, J = 7.7 Hz, 1H, ArH, H_2), 7.38 (d, J = 8.3 Hz, 1H, ArH, H_4), 5.11 (s, 1H, CH, H_7), 2.89-2.85 (m, 1H, CH_2, H_9), 2.78-2.67 (m, 1H, CH_2, H_9), 2.34-2.26 (m, 2H, CH_2, H_{11}), 2.07-1.99 (m, 1H, CH_2, H_{10}), 1.93-1.85 (m, 1H, CH_2, H_{10}); ^{13}C NMR (75 MHz, DMSO-d_6): δ (ppm): 195.41 (C_8), 160.71 (C_6), 153.66 (C_{4a}), 152.80 (C_{11a}), 152.63 (C_{13}), 146.44 (C_{12a}), 143.07 (C_{16}), 132.71 (C_3), 129.64 (C_{14},C_{18}), 124.56 (C_1), 123.78 (C_{15},C_{17}), 123.62 (C_2), 117.41 (C_4), 113.32 (C_{12b}), 111.48 (C_{7a}), 101.15 (C_{6a}), 37.02 (C_9), 35.50 (C_7), 26.89 (C_{11}), 21.13 (C_{10}); MS: (m/z, %): 388 (M^+,10), 386 (M^+-2, 28), 266 (35), 28 (100); Anal. Calcd. for $C_{22}H_{17}NO_3$(388): C: 68.04, H: 4.15, N: 7.21%. Found: C: 67.91, H: 4.08, N: 7.12%.

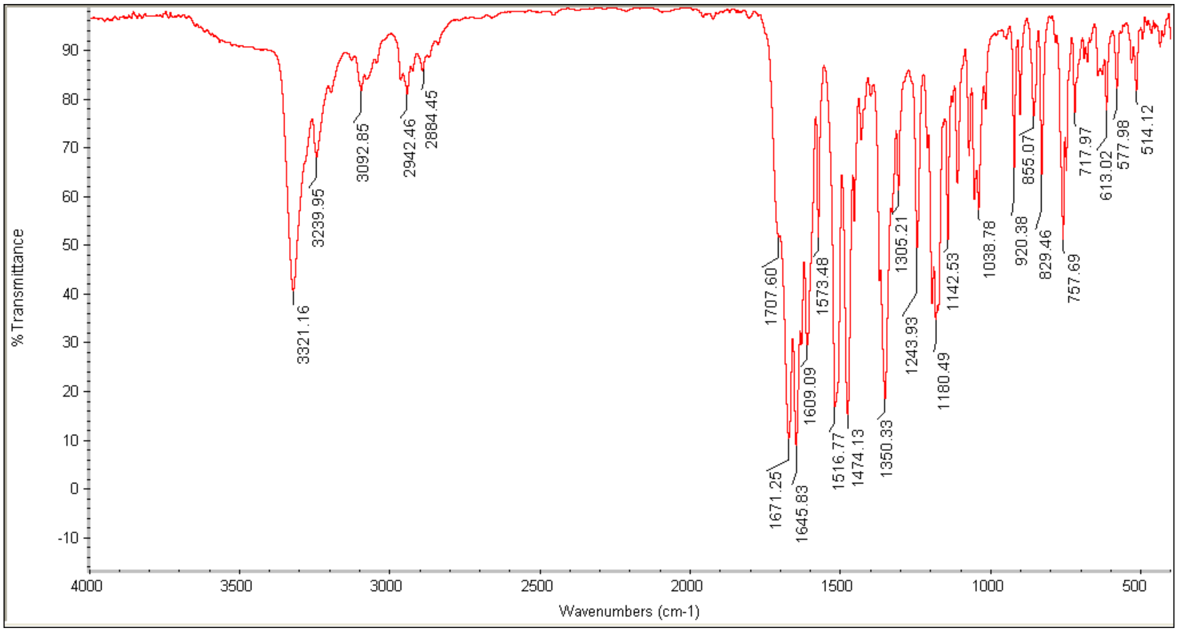

Figure 6. IR spectrum of compound **2b**

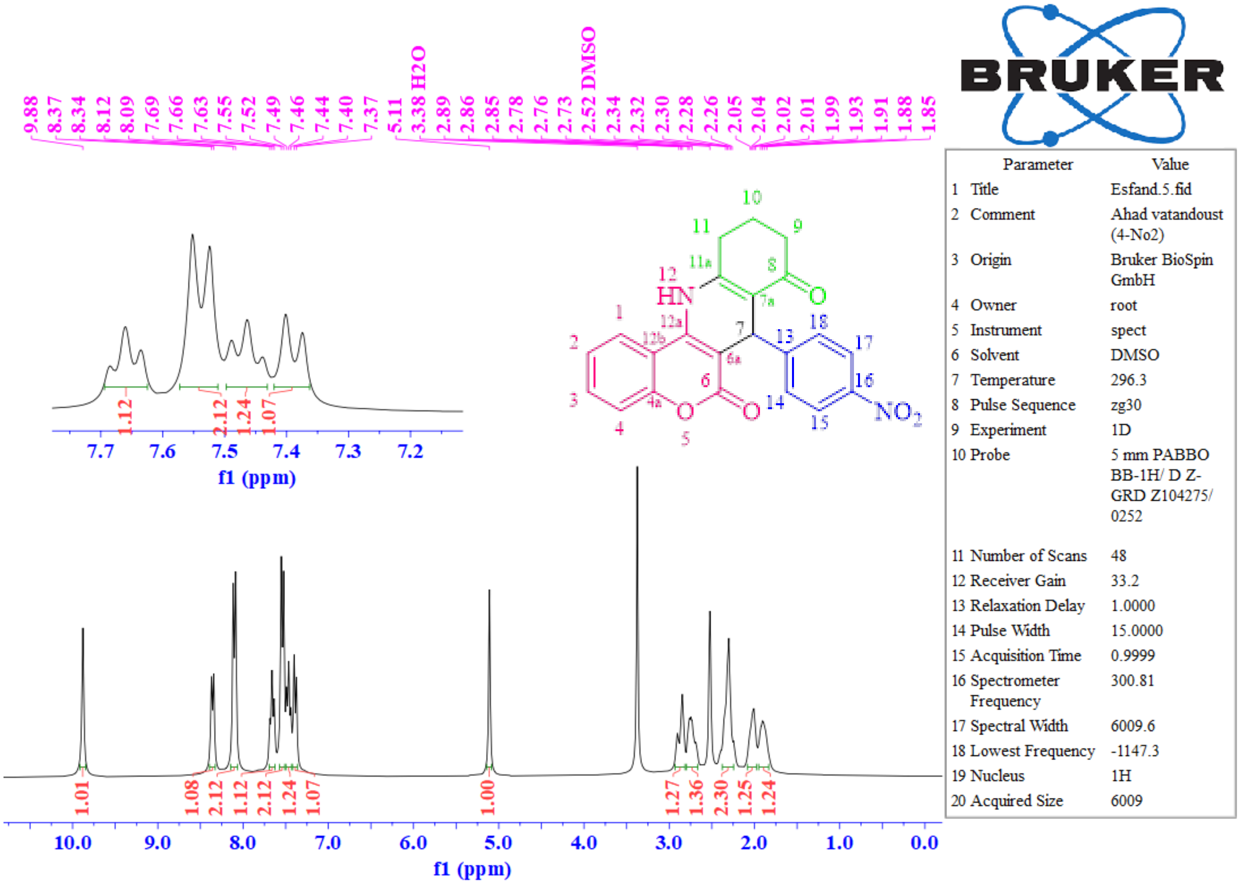

Figure 7. ^{1}H NMR (300 MHz, DMSO-d_6) spectrum of compound **2b**

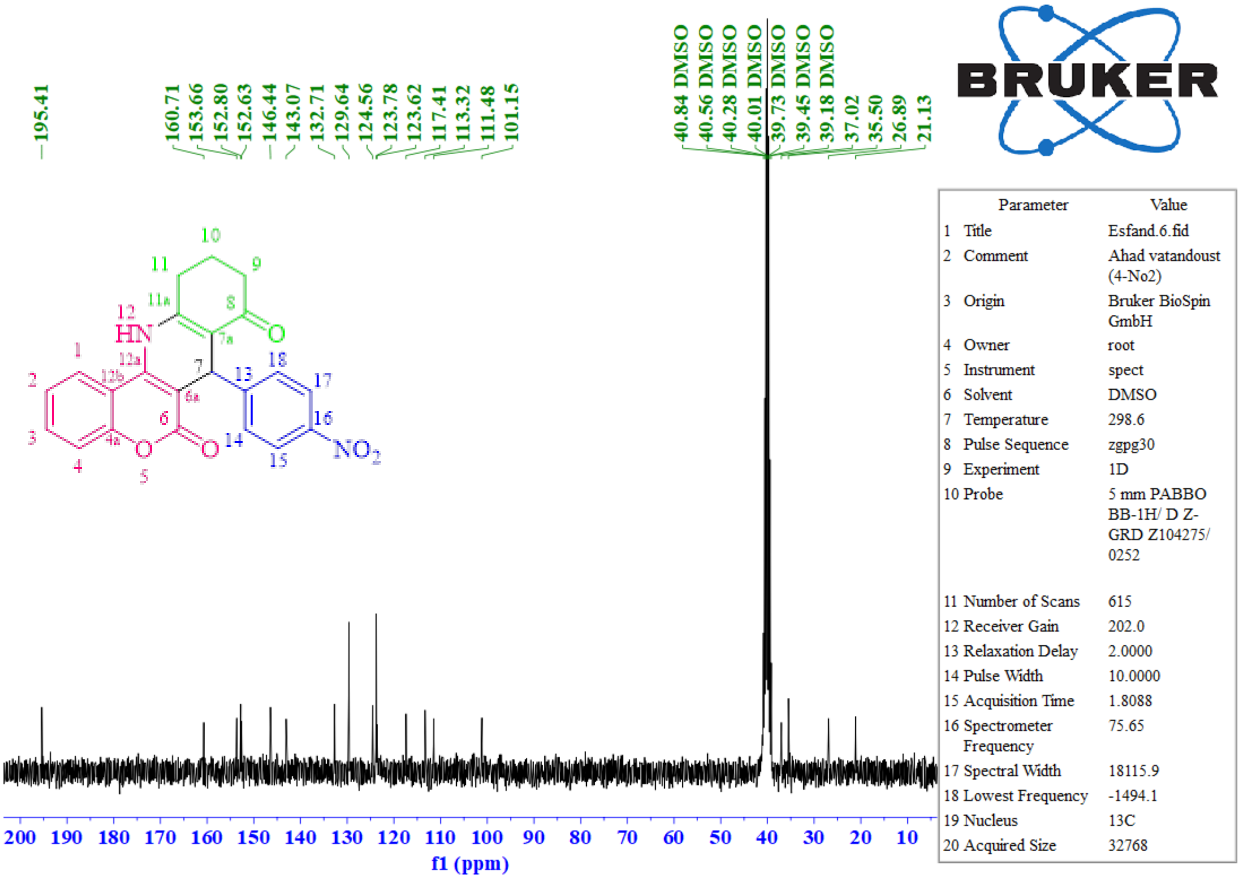

Figure 8. ^{13}C NMR (75 MHz, DMSO-d_6) spectrum of compound **2b**

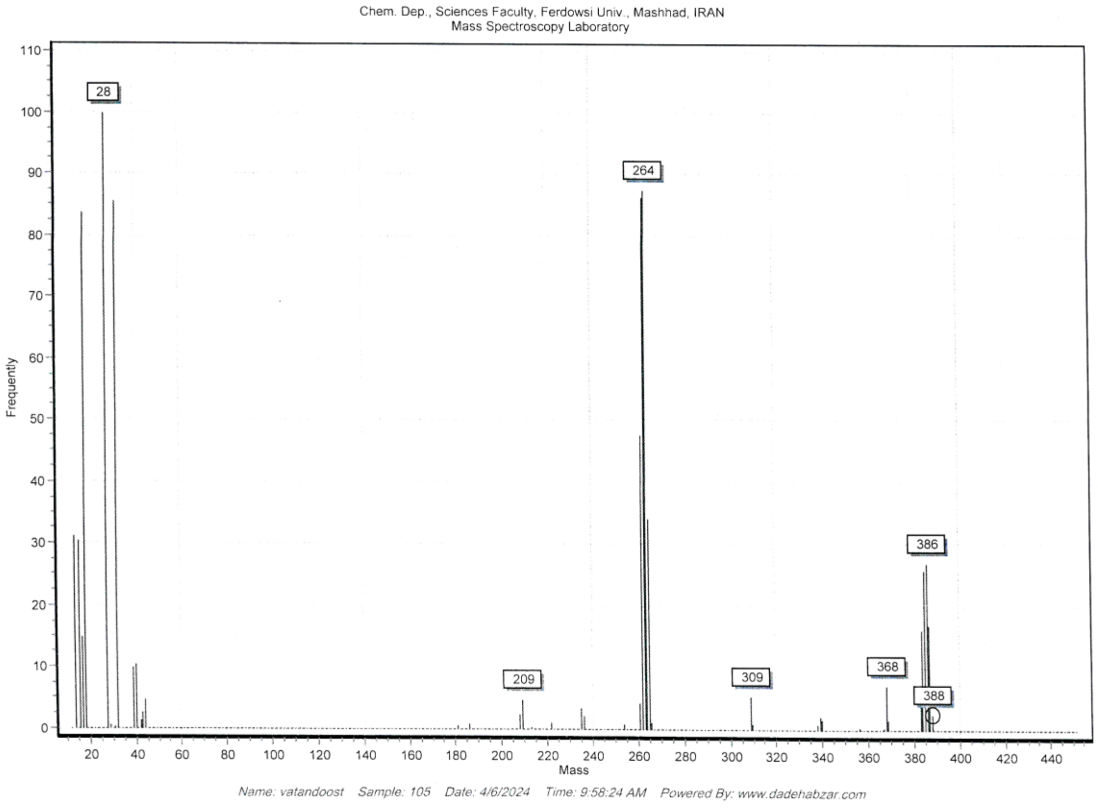

Figure 9. Mass spectrum of compound **2b**

Eager 300 Summarize Results

Date: 24/04/2024 at 13:05:03

Method Name: NCHS

Method Filename: Copy of N C H S-bkp.mth

Filename		As Method				Vial
Vatandoust-174						
# Group Sample Name		Tayp	Weig.	Prof.	F ---	-----
174-1	105	UNK	0.643	6.25	---	-----
Component Name	Element%					
Nitrogen%	7.121024971					
Carbon%	67.91162109					
Hydrogen%	4.087371998					
Sulphur%	0					

1 Sample (s) in Group No:1

Component Name	Average
Nitrogen%	7.121024971
Carbon%	67.91162109
Hydrogen%	4.087371998
Sulphur%	0

Figure 10. CHNS spectrum of compound **2b**

Anal. Calcd. for $C_{22}H_{17}NO_3$(388)		
C: 68.04 %	H: 4.15 %	N: 7.21 %

7-(4-cyanophenyl)-7,10,11,12-tetrahydro-6H-chromeno[4,3-b]quinoline-6,8(9H)-dione (2c)

Light yellow solid; (0.349g, 95%); Mp=336-338 °C; IR (KBr) (υ_{max}/cm^{-1}): 3341 (NH), 3096, 3051 (C-H aromatic), 2937, 2880 (C-H aliphatic), 2224 (CN), 1684 (C=O), 1640,1605 (C=C); ^{1}H NMR (300 MHz, DMSO-d_6): δ (ppm): 9.85 (s, 1H, NH), 8.35 (d, J = 8.0 Hz, 1H, ArH, H_1), 7.71-7.63 (m, 3H, ArH, H_2, H_3, H_4), 7.48-7.37 (m, 4H, ArH, H_{14}, H_{15}, H_{17}, H_{18}), 5.06 (s, 1H, CH, H_7), 2.90-2.84 (m, 1H, CH_2, H_9), 2.75-2.67 (m, 1H, CH_2, H_9), 2.33-2.28 (m, 2H, CH_2, H_{11}), 2.02-1.98(m, 1H, CH_2, H_{10}), 1.93-1.85 (m, 1H, CH_2, H_{10}); ^{13}C NMR (75 MHz, DMSO-d_6): δ (ppm): 195.42 (C_8), 160.74 (C_6), 152.74 (C_{4a}), 152.60 (C_{11a}), 151.64 (C_{12a}), 143.01 (C_{13}), 132.66 (C_3), 132.54 (C_{15},C_{17}), 129.40 (C_{14},C_{18}), 124.54 (C_1), 123.59 (C_2), 119.37 (CN), 117.39 (C_4), 113.33 (C_{12b}), 111.51 (C_{7a}), 109.52 (C_{16}), 101.23 (C_{6a}), 37.02 (C_9), 35.54 (C_7), 26.86 (C_{11}), 21.14 (C_{10}); MS: (m/z, %): 368 (M^+, 10), 366 (M^+-2, 70), 266 (80), 28 (75); Anal. Calcd. for $C_{23}H_{16}N_2O_3$(368): C: 74.99, H: 4.38, N: 7.60%. Found: C: 74.82, H: 4.30, N: 7.55%.

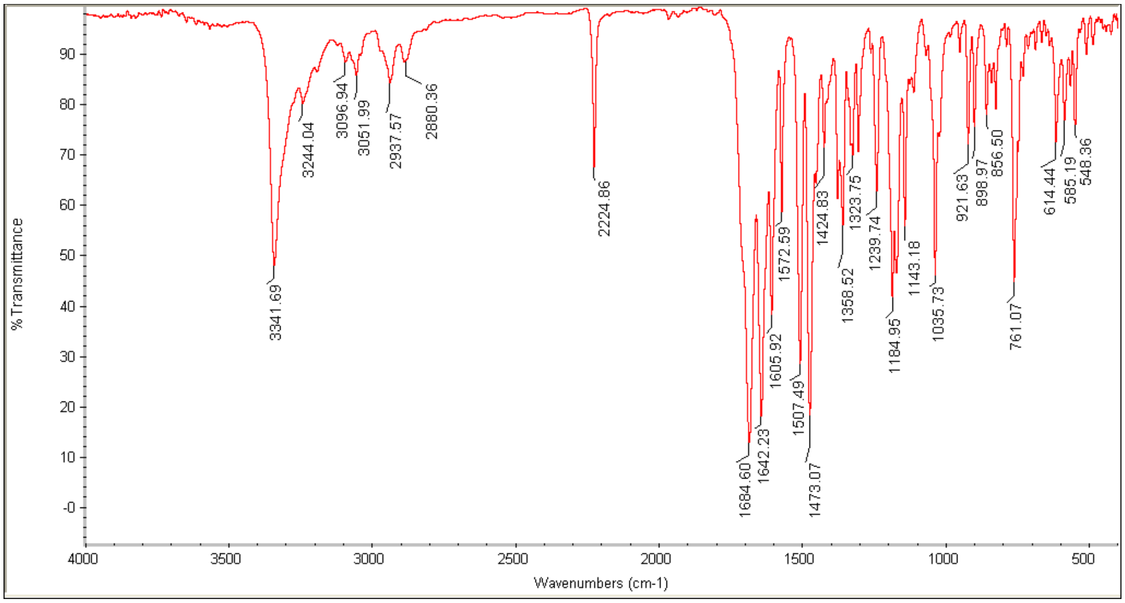

Figure 11. IR spectrum of compound **2c**

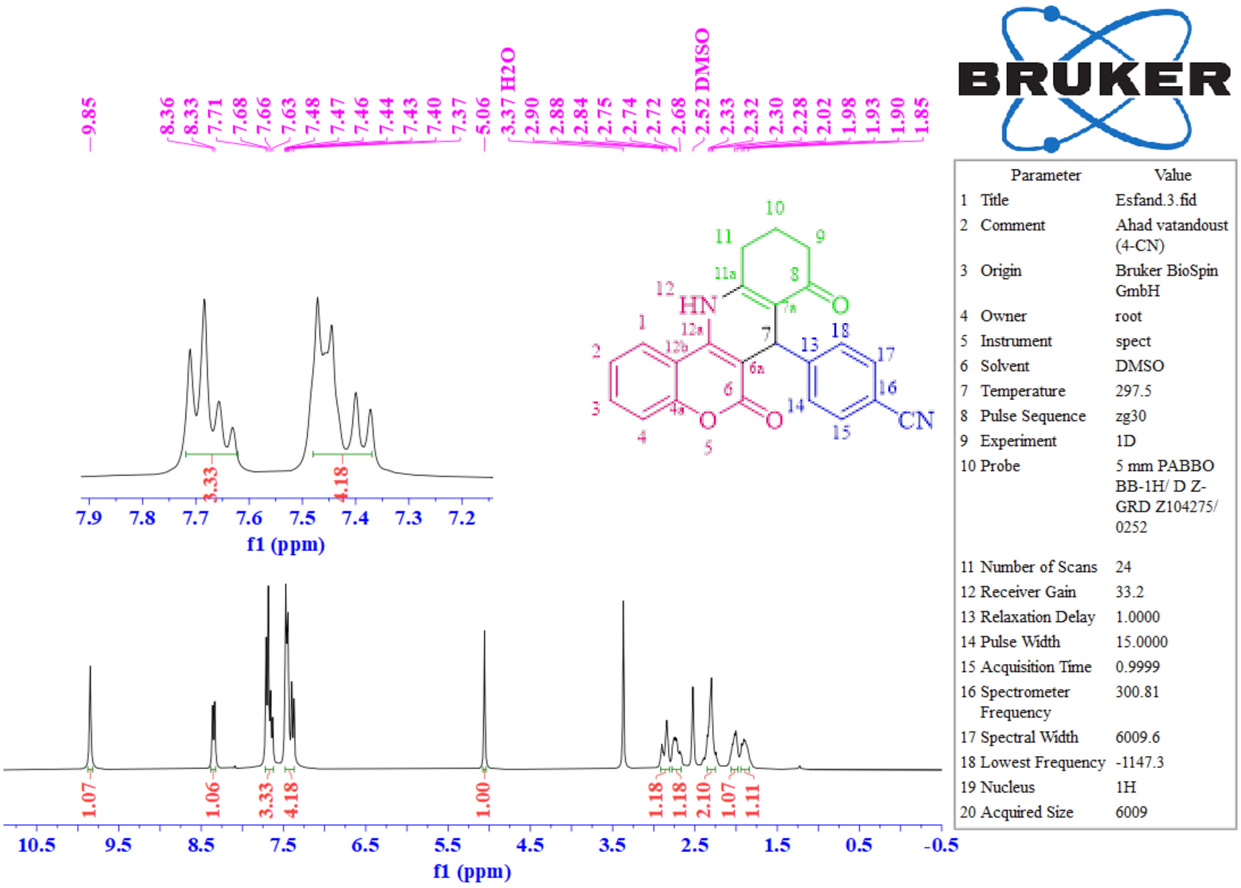

Figure 12. ^{1}H NMR (300 MHz, DMSO-d_6) spectrum of compound **2c**

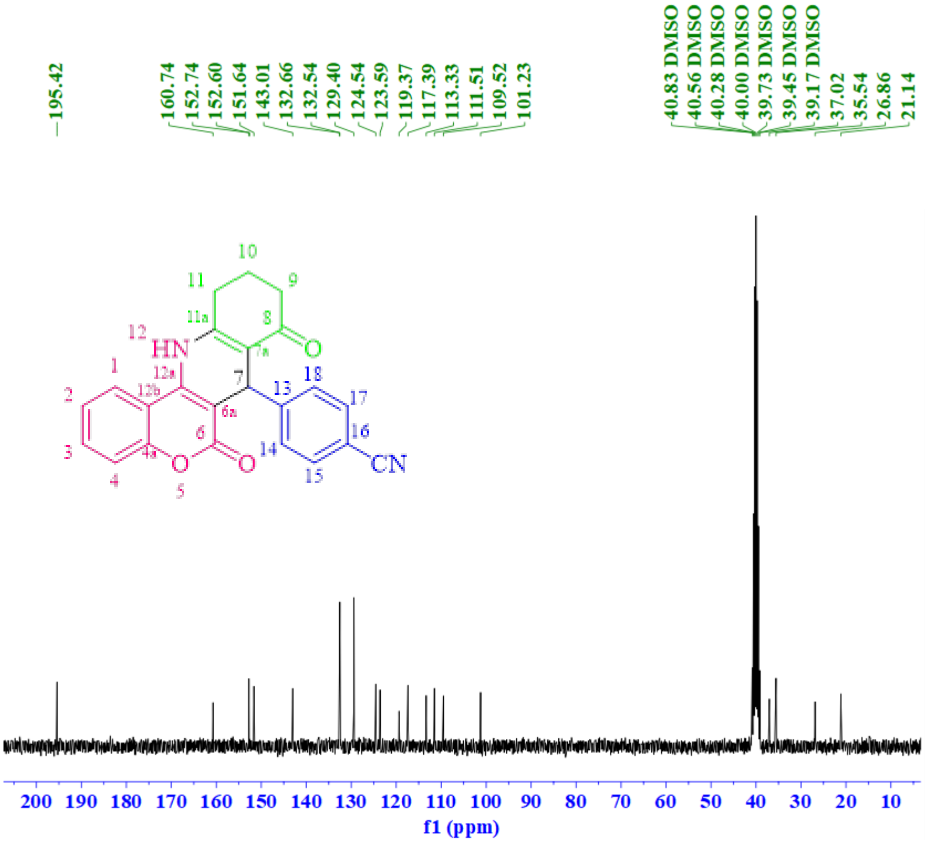

Parameter		Value
1	Title	Esfand.4.fid
2		Ahad vatandoust (4-CN)
3	Origin	Bruker BioSpin GmbH
4	Owner	root
5	Instrument	spect
6	Solvent	DMSO
7	Temperature	297.0
8	Pulse Sequence	zgpg30
9	Experiment	1D
10	Probe	5 mm PABBO BB-1H/ D Z-GRD Z104275/ 0252
11	Number of Scans	302
12	Receiver Gain	202.0
13	Relaxation Delay	2.0000
14	Pulse Width	10.0000
15	Acquisition Time	1.8088
16	Spectrometer Frequency	75.65
17	Spectral Width	18115.9
18	Lowest Frequency	-1494.1
19	Nucleus	13C
20	Acquired Size	32768

Figure 13. ^{13}C NMR (75 MHz, DMSO-d_6) spectrum of compound **2c**

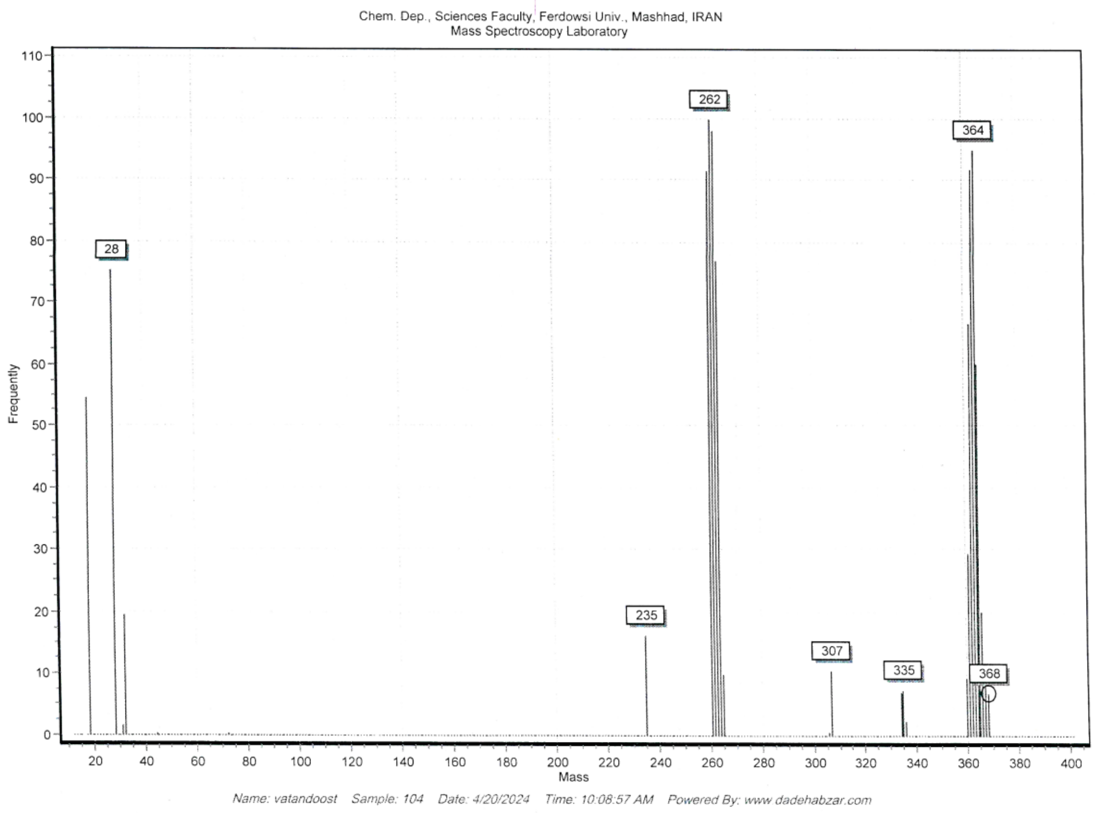

Figure 14. Mass spectrum of compound **2c**

Eager 300 Summarize Results

Date: 24/04/2024 at 13:05:18

Method Name: NCHS

Method Filename: Copy of N C H S-bkp.mth

Filename		As Method				Vial
Vatandoust-175						
# Group Sample Name		Tayp Weig. Prof. F			---	-----
175-1	104	UNK	0.645	6.25	---	-----

Component Name	Element%
Nitrogen%	7.558488731
Carbon%	74.82396332
Hydrogen%	4.304181957
Sulphur%	0

1 Sample (s) in Group No:1

Component Name	Average
Nitrogen%	7.558488731
Carbon%	74.82396332
Hydrogen%	4.304181957
Sulphur%	0

Figure 15. CHNS spectrum of compound **2c**

Anal. Calcd. for $C_{23}H_{16}N_2O_3$(368)		
C: 74.99 %	H: 4.38 %	N: 7.60 %

7-(4-chlorophenyl)-7,10,11,12-tetrahydro-6H-chromeno[4,3-b]quinoline-6,8(9H)-dione (2d)[3]

Light yellow solid; (0.32g, 85%) Mp=215-216 °C (Lit. 214-216 °C); IR (KBr) (υ_{max}/cm^{-1}): 3334 (NH), 3051 (C-H aromatic), 2941, 2884 (C-H aliphatic), 1673 (C=O), 1643, 1607 (C=C); ^1H NMR (300 MHz, DMSO-d_6): δ (ppm): 9.80 (s, 1H, NH), 8.35 (d, J = 8.0 Hz, 1H, ArH, H_1), 7.65 (t, J = 7.7 Hz, 1H, ArH, H_3), 7.45 (t, J = 7.6 Hz, 1H, ArH, H_2), 7.40 (d, J = 8.3 Hz, 1H, ArH, H_4), 7.27 (s, 4H, ArH, H_{14}, H_{15}, H_{17}, H_{18}), 4.99 (s, 1H, CH, H_7), 2.89-2.83 (m, 1H, CH_2, H_9), 2.76-2.71 (m, 1H, CH_2, H_9), 2.33-2.28 (m, 2H, CH_2, H_{11}), 2.04-2.00 (m, 1H, CH_2, H_{10}), 1.93-1.88 (m, 1H, CH_2, H_{10}); ^{13}C NMR (75 MHz, DMSO-d_6): δ (ppm): 195.44 (C_8), 160.78 (C_6), 152.55 (C_{4a}), 152.32 (C_{11a}), 145.33 (C_{12a}), 142.69 (C_{13}), 132.52 (C_3), 131.26 (C_{16}), 130.10 ($C_{15,}C_{17}$), 128.45 ($C_{14,}C_{18}$), 124.50 (C_1), 123.51 (C_2), 117.37 (C_4), 113.42 (C_{12b}), 112.03 (C_{7a}), 101.82 (C_{6a}), 37.10 (C_9), 34.44 (C_7), 26.85 (C_{11}), 21.18 (C_{10}); MS: (m/z, %): 377 (M^+, 20), 375 (M^+-2, 65), 266 (50), 28(100); Anal. Calcd. for $C_{22}H_{16}ClNO_3$(377): C: 69.94, H: 4.27, N: 3.71%. Found: C: 69.88, H: 4.17, N: 3.64%.

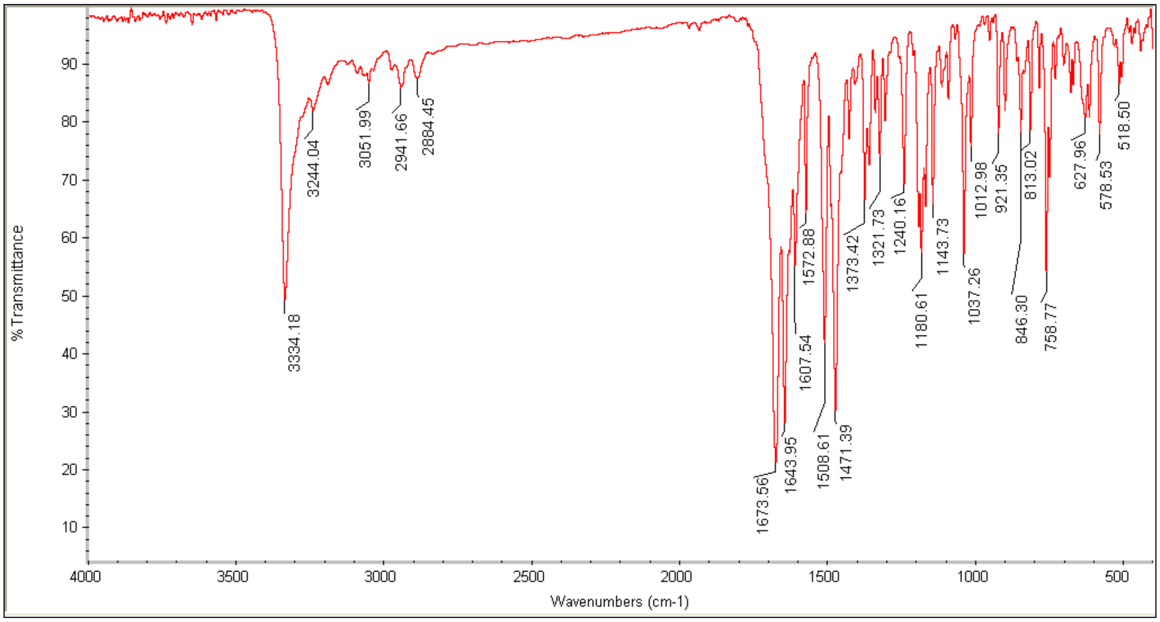

Figure 16. IR spectrum of compound **2d**

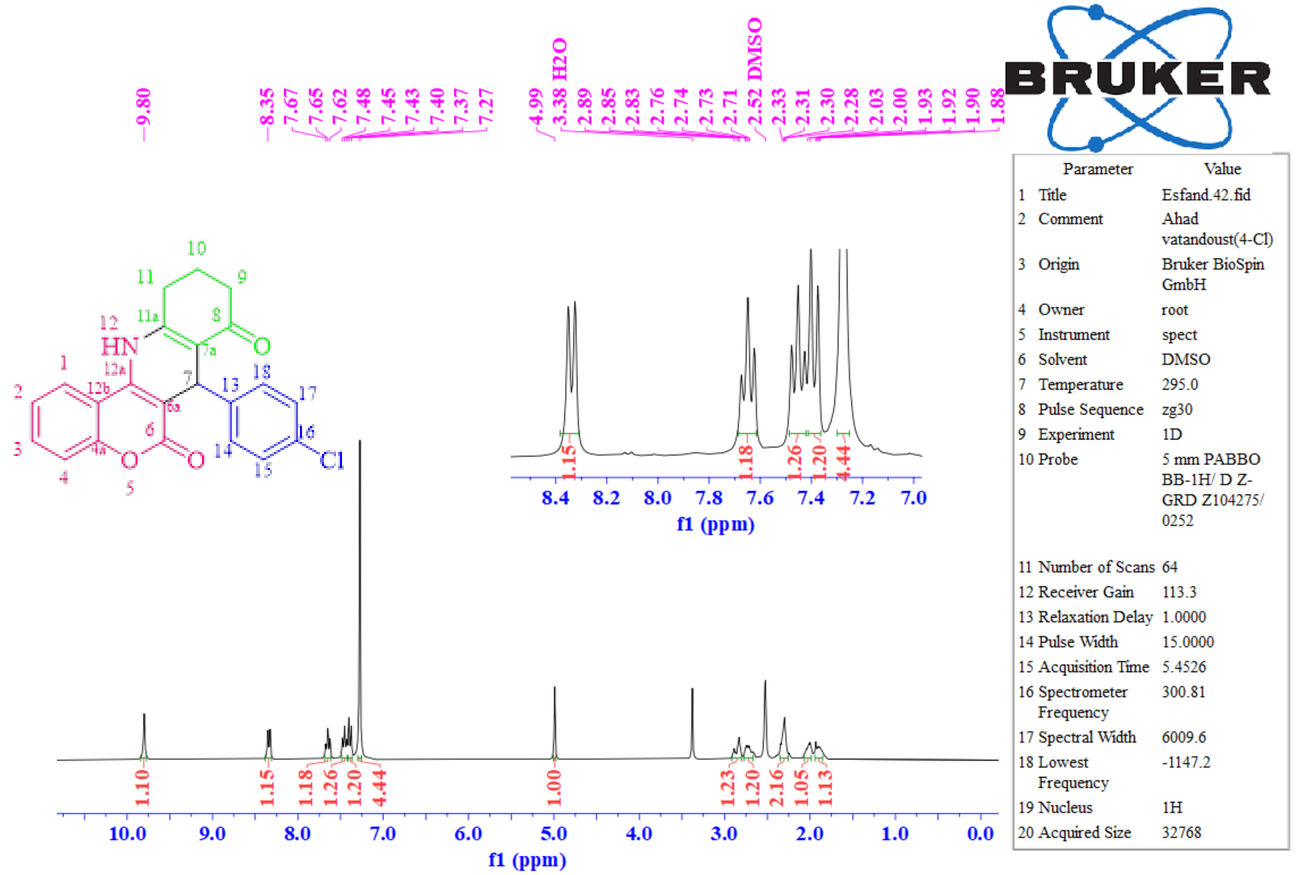

Figure 17. ^{1}H NMR (300 MHz, DMSO-d_6) spectrum of compound **2d**

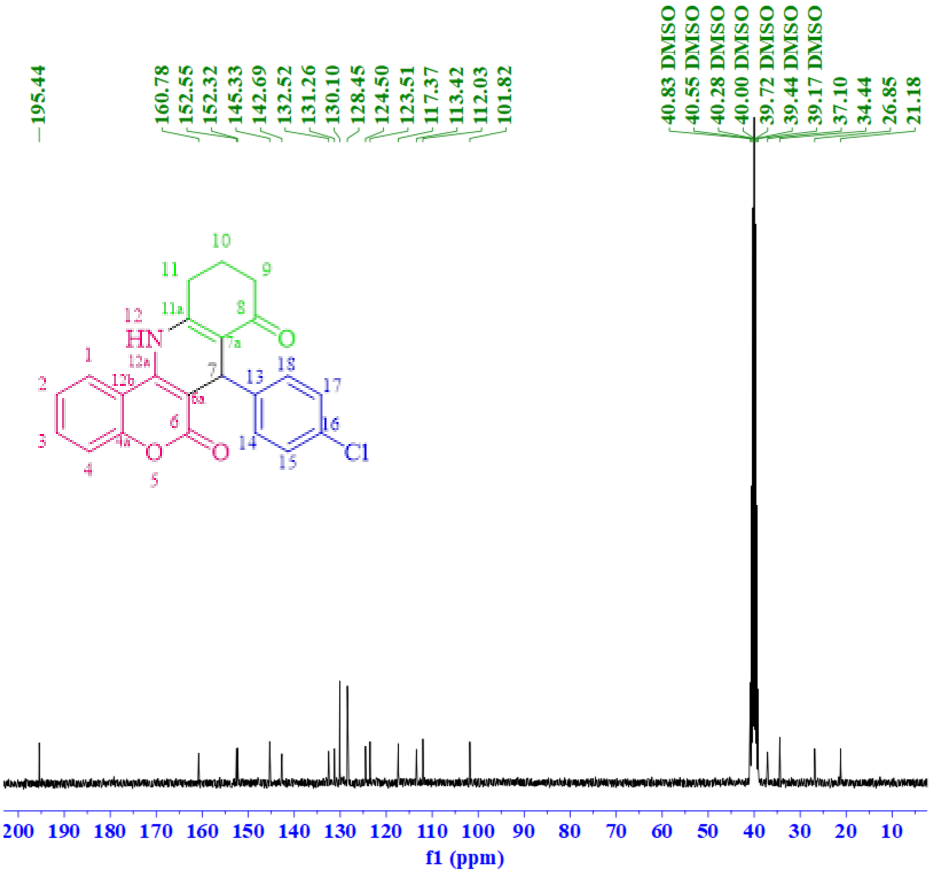

	Parameter	Value
1	Title	Esfand.43.fid
2	Comment	Ahad vatandoust 4-Cl
3	Origin	Bruker BioSpin GmbH
4	Owner	root
5	Instrument	spect
6	Solvent	DMSO
7	Temperature	295.9
8	Pulse Sequence	zgpg30
9	Experiment	1D
10	Probe	5 mm PABBO BB-1H/ D Z-GRD Z104275/ 0252
11	Number of Scans	1006
12	Receiver Gain	202.0
13	Relaxation Delay	2.0000
14	Pulse Width	10.0000
15	Acquisition Time	1.8088
16	Spectrometer Frequency	75.65
17	Spectral Width	18115.9
18	Lowest Frequency	-1494.8
19	Nucleus	13C
20	Acquired Size	32768

Figure 18. ^{13}C NMR (75MHz, DMSO-d_6) spectrum of compound **2d**

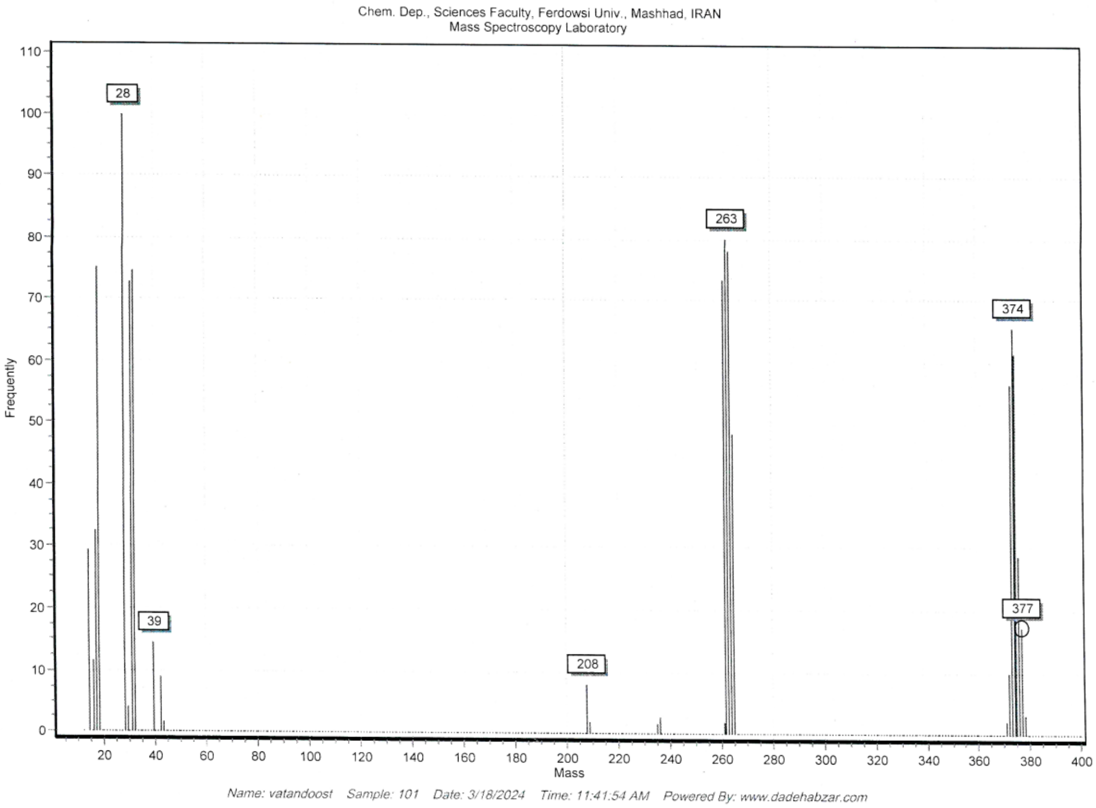

Figure 19. Mass spectrum of compound **2d**

Eager 300 Summarize Results

Date: 24/04/2024 at 13:07:15

Method Name: NCHS

Method Filename: Copy of N C H S-bkp.mth

Filename		As Method				Vial
Vatandoust-186						
# Group Sample Name		Tayp	Weig.	Prof.	F ---	-----
186-1	101	UNK	0.673	6.25	---	-----
Component Name	Element%					
Nitrogen%	3.649792671					
Carbon%	69.88464691					
Hydrogen%	4.171821172					
Sulphur%	0					

1 Sample (s) in Group No:1

Component Name	Average
Nitrogen%	3.649792671
Carbon%	69.88464691
Hydrogen%	4.171821172
Sulphur%	0

Anal. Calcd. for $C_{22}H_{16}ClNO_3$(377)		
C: 69.94 %	H: 4.27 %	N: 3.71 %

Figure 20. CHNS spectrum of compound **2d**

7-(4-bromophenyl)-7,10,11,12-tetrahydro-6H-chromeno[4,3-b]quinoline-6,8(9H)-dione (2e)[3]

Light yellow solid; (0.336g, 80%); Mp=295-296°C (Lit. 294-296 °C); IR (KBr) (υ_{max}/cm^{-1}): 3331(NH), 3043 (C-H aromatic), 2941, 2884 (C-H aliphatic), 1670 (C=O); MS: (m/z, %): 421 (M^+, 48), 419 (M^+-2,60), 266 (28), 28 (100); Anal. Calcd. for $C_{22}H_{16}BrNO_3$(421): C: 62.58, H: 3.82, N: 3.32 %. Found: C: 62.38, H: 3.67, N: 3.18%.

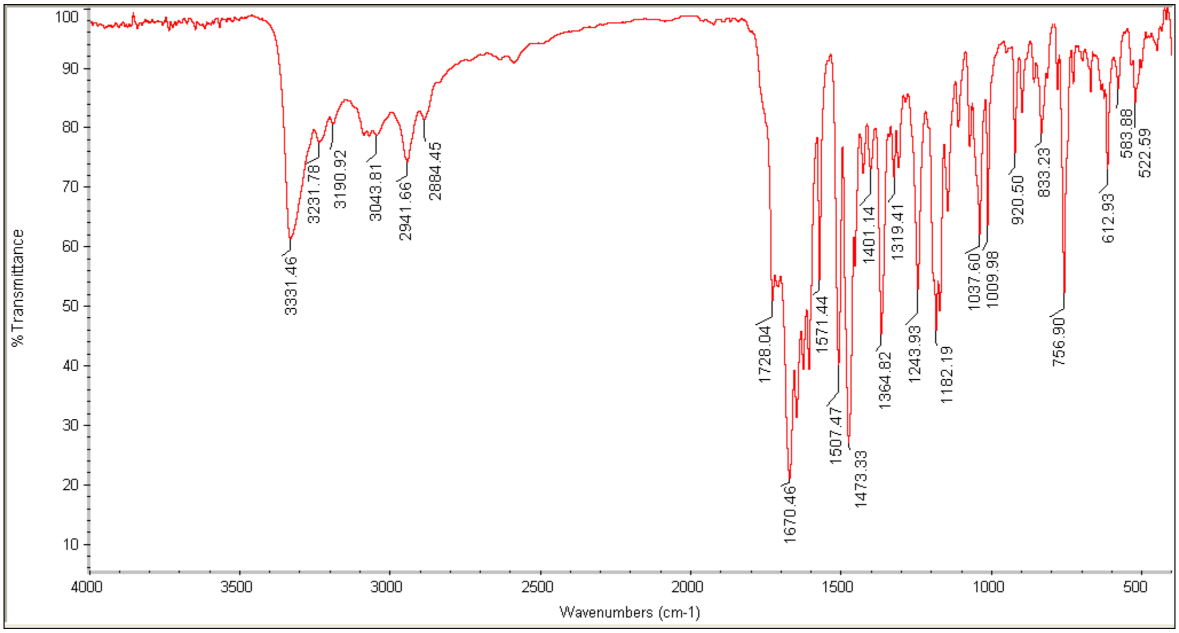

Figure 21. IR spectrum of compound **2e**

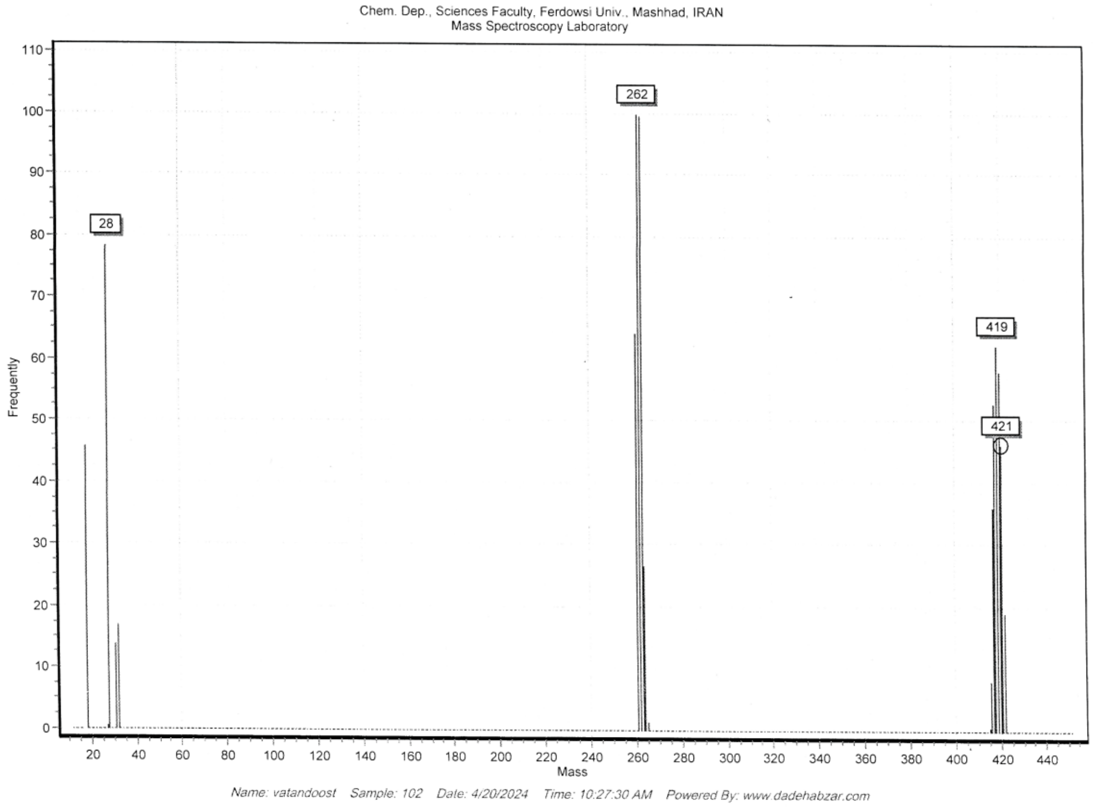

Figure 22. Mass spectrum of compound **2e**

Eager 300 Summarize Results

Date: 24/04/2024 at 13:07:05

Method Name: NCHS

Method Filename: Copy of N C H S-bkp.mth

Filename		As Method				Vial
Vatandoust-184						
# Group Sample Name		Tayp Weig. Prof. F			---	-----
184-1	102	UNK	0.618	6.25	---	-----

Component Name	Element%
Nitrogen%	3.185758247
Carbon%	62.3849939
Hydrogen%	3.670049191
Sulphur%	0

1 Sample (s) in Group No:1

Component Name	Average
Nitrogen%	3.185758247
Carbon%	62.3849939
Hydrogen%	3.670049191
Sulphur%	0

Figure 23. CHNS spectrum of compound **2e**

Anal. Calcd. for $C_{22}H_{16}BrNO_3$(421)		
C: 62.58 %	H: 3.82 %	N: 3.32 %

7-(3-bromophenyl)-7,10,11,12-tetrahydro-6H-chromeno[4,3-b]quinoline-6,8(9H)-dione (2f)[3]

Light yellow solid; (0.33g, 80%) Mp=265-266 °C (Lit. 267-269 °C); IR (KBr) (υ_{max}/cm^{-1}): 3324(NH), 3092(C-H aromatic), 2942, 2892 (C-H aliphatic), 1668 (C=O), 1633,1606 (C=C); ^1H NMR (300 MHz, DMSO-d_6): δ (ppm): 9.85 (s, 1H, NH), 8.35 (d, J = 8.1 Hz, 1H, ArH, H_1), 7.68 (t, J = 7.8 Hz, 1H, ArH, H_3), 7.48 (t, J = 7.2 Hz, 1H, ArH, H_2), 7.43-7.41 (m, 2H, ArH, H_{14}, H_{16}), 7.34 (d, J = 7.0 Hz, 1H, ArH, H_4), 7.25-7.20 (m, 2H, ArH, H_{17},H_{18}), 4.99 (s, 1H, CH, H_7), 2.89-2.84 (m, 1H, CH_2, H_9), 2.77-2.72 (m, 1H,CH_2, H_9), 2.36-2.30 (m, 2H, CH_2, H_{11}), 2.07-1.99 (m, 1H, CH_2, H_{10}), 1.93-1.88 (m, 1H, CH_2, H_{10}); ^{13}C NMR (75 MHz, DMSO-d_6): δ (ppm): 195.49 (C_8), 160.80 (C_6), 152.57 (C_{4a}), 148.95 (C_{11a}), 142.85 (C_{12a}), 132.64 (C_{13}), 130.99 (C_3), 130.94 (C_{14},C_{16}), 129.65 (C_{17}), 127.26 (C_{18}), 124.57 (C_1), 123.58 (C_{15}), 121.76 (C_2), 117.44 (C_4), 113.40 (C_{12b}), 111.80 (C_{7a}), 101.60 (C_{6a}), 37.08 (C_9), 34.88 (C_7), 26.86 (C_{11}), 21.19 (C_{10}); MS: (m/z, %): 421 (M^+, 78), 419 (M^+-2, 85), 266 (68), 28 (100); Anal. Calcd. for $C_{22}H_{16}BrNO_3$(421): C: 62.58, H: 3.82, N: 3.32%. Found: C: 62.45, H: 3.69, N: 3.28%.

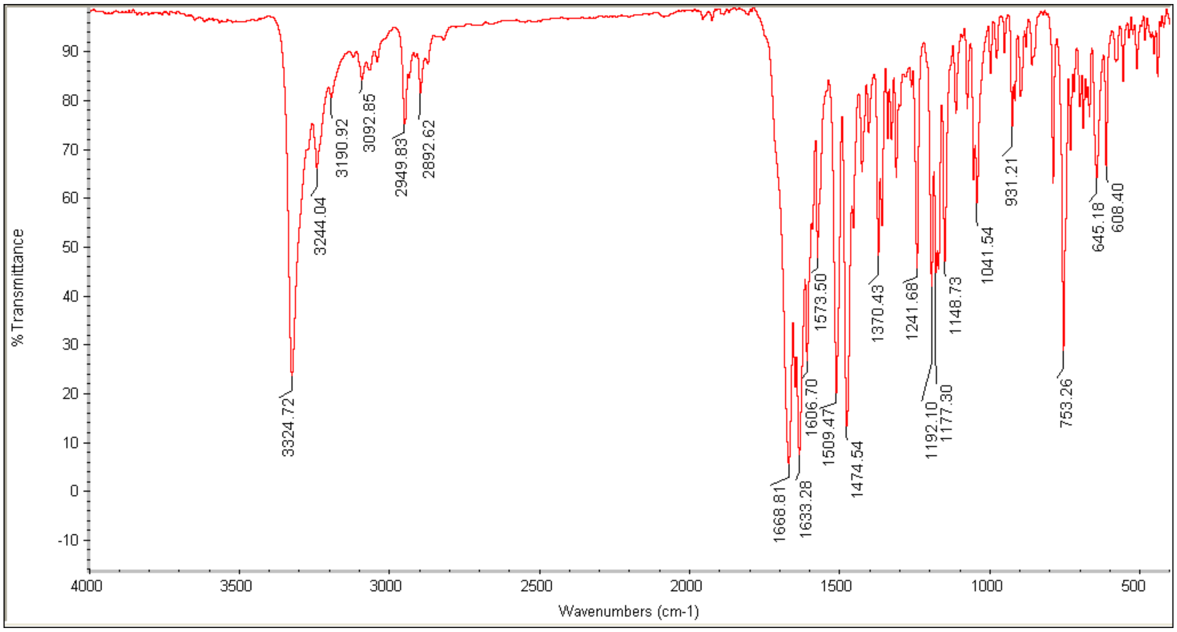

Figure 24. IR spectrum of compound **2f**

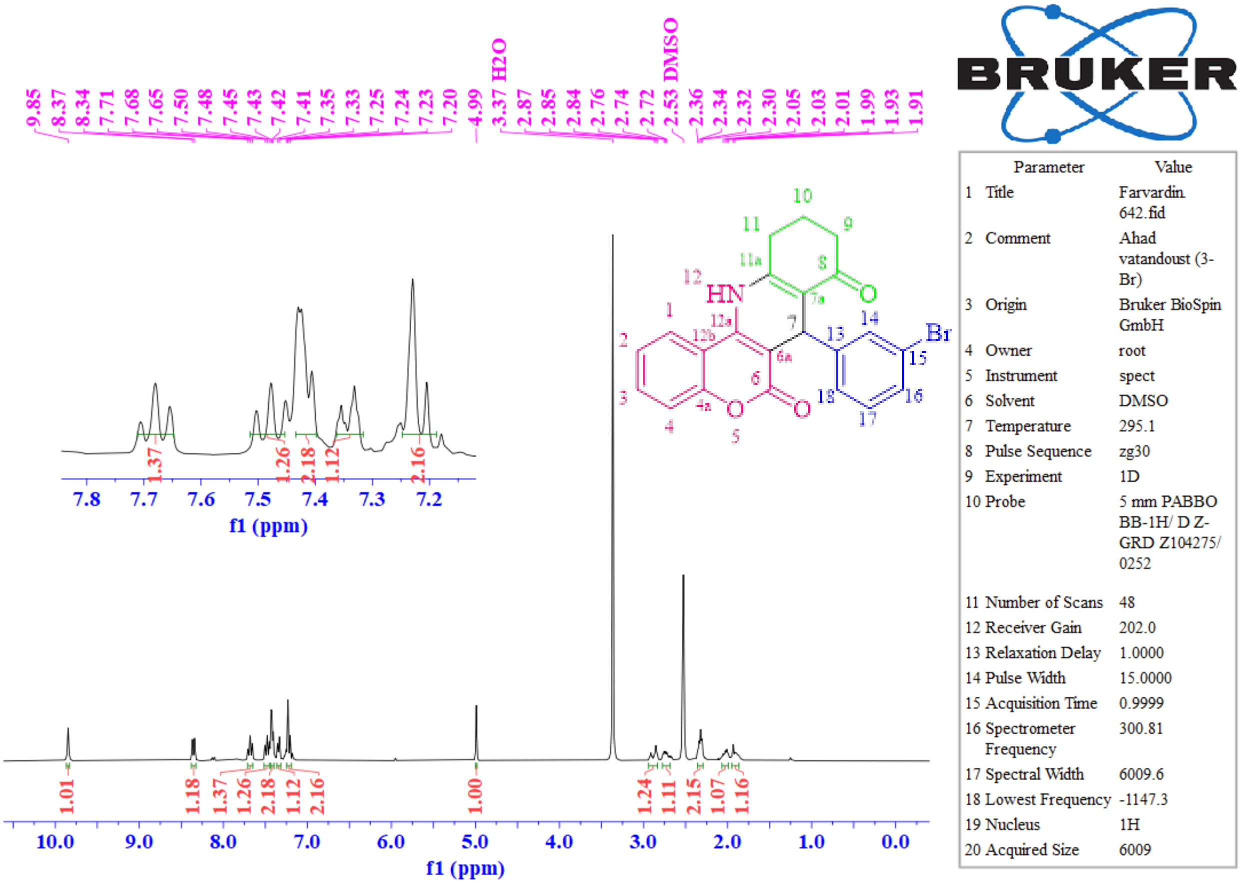

Figure 25. ^{1}H NMR (300 MHz, DMSO-d_6) spectrum of compound **2f**

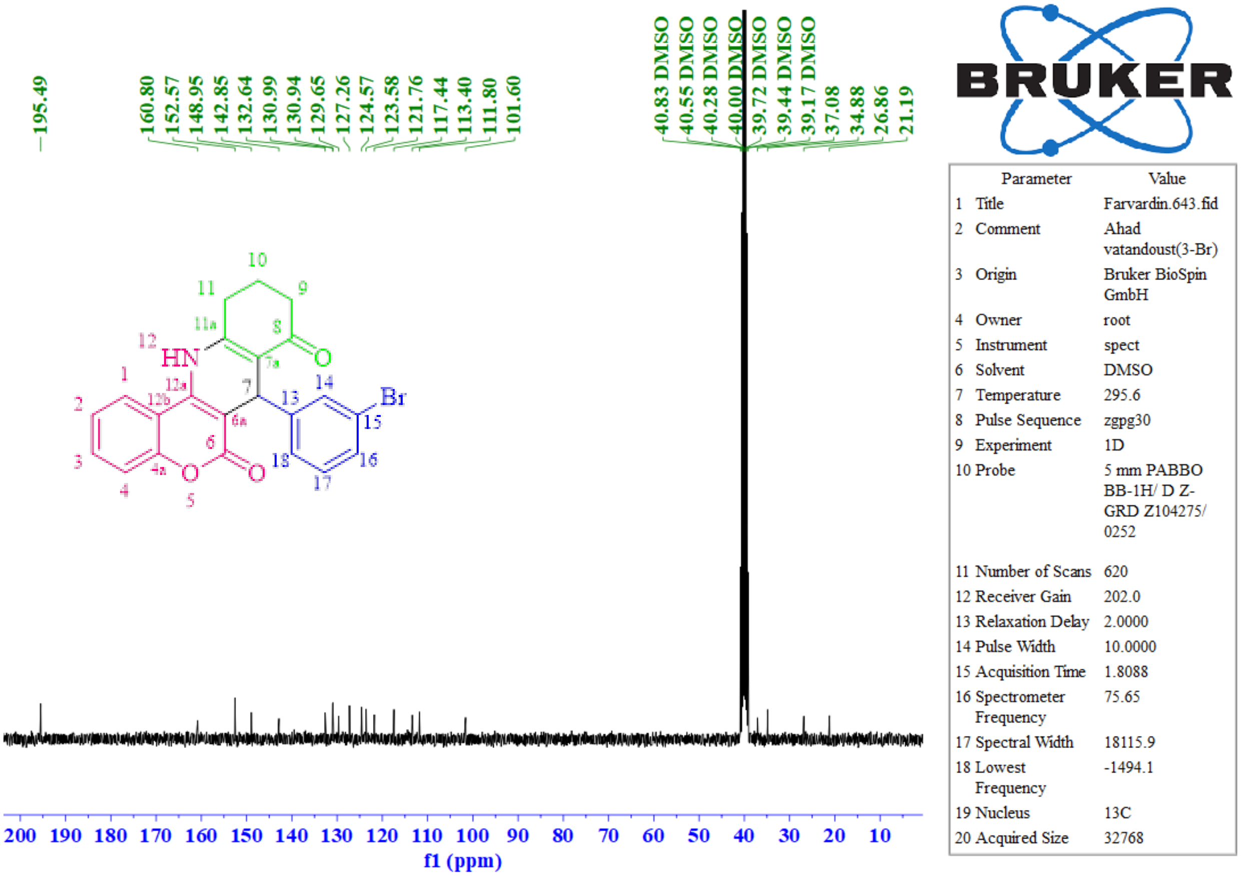

Figure 26. ^{13}C NMR (75 MHz, DMSO-d_6) spectrum of compound **2f**

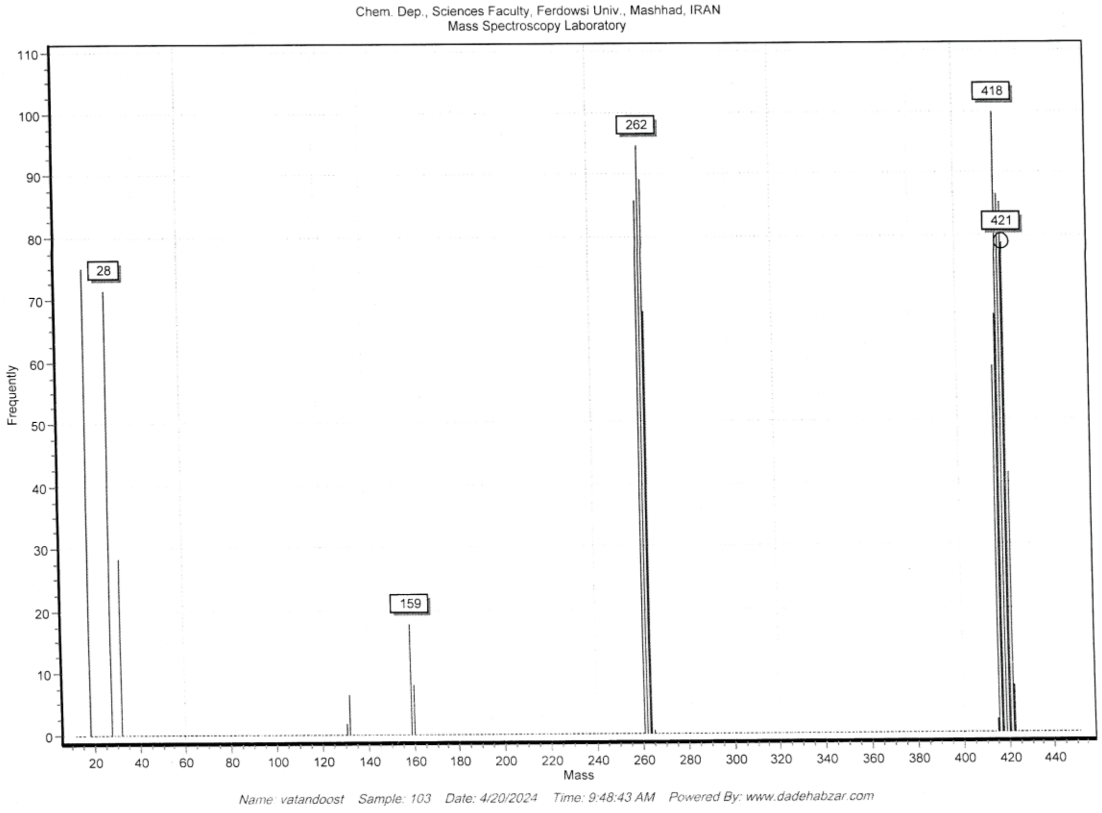

Figure 27. Mass spectrum of compound **2f**

Eager 300 Summarize Results

Date: 24/04/2024 at 13:06:50

Method Name: NCHS

Method Filename: Copy of N C H S-bkp.mth

Filename		As Method				Vial
Vatandoust-183						
# Group Sample Name		Tayp Weig. Prof. F			---	-----
183-1	103	UNK	0.604	6.25	---	-----

Component Name	Element%
Nitrogen%	3.281792812
Carbon%	62.45877167
Hydrogen%	3.691780491
Sulphur%	0

1 Sample (s) in Group No:1

Component Name	Average
Nitrogen%	3.281792812
Carbon%	62.45877167
Hydrogen%	3.691780491
Sulphur%	0

Figure 28. CHNS spectrum of compound **2b**

Anal. Calcd. for $C_{22}H_{16}BrNO_3$(421)		
C: 62.58 %	H: 3.82 %	N: 3.32 %

7-(4-hydroxyphenyl)-7,10,11,12-tetrahydro-6H-chromeno[4,3-b]quinoline-6,8(9H)-dione (2g)[1]

White solid; (0.287g, 80%); Mp=337-339 °C (Lit. 338-340 °C); IR (KBr) (υ_{max}/cm^{-1}): 3344(NH), 3288(OH), 3088 (C-H aromatic), 2925, 2876 (C-H aliphatic), 1672 (C=O), 1634,1607 (C=C); ^{1}H NMR (300 MHz, DMSO-*d6*): δ (ppm): 9.70 (s, 1H, NH), 9.17 (s, 1H, OH), 8.31 (d, *J* = 8.1 Hz, 1H, ArH, H_1), 7.62 (t, *J* = 7.8 Hz, 1H, ArH, H_3), 7.43 (t, *J* = 7.6 Hz, 1H, ArH, H_2), 7.37 (d, *J* = 8.3 Hz, 1H, ArH, H_4), 7.04 (d, *J* = 8.1 Hz, 2H, ArH, H_{14}, H_{18}), 6.61 (d, *J* = 8.0 Hz, 2H, ArH, H_{15}, H_{17}), 4.91 (s, 1H, CH, H_7), 2.88-2.82 (m, 1H, CH_2, H_9), 2.74-2.67 (m, 1H, CH_2, H_9), 2.32-2.28 (m, 2H, CH_2, H_{11}), 2.04-2.00 (m, 1H, CH_2, H_{10}), 1.94-1.89 (m, 1H, CH_2, H_{10}); ^{13}C NMR (75 MHz, DMSO-*d6*): δ (ppm): 195.47 (C_8), 160.87 (C_6), 156.24 (C_{16}), 152.44 (C_{4a}), 151.72 (C_{11a}), 142.10 (C_{12a}), 137.12 (C_{13}), 132.22 (C_3), 129.09 (C_{14},C_{18}), 124.39 (C_1), 123.33 (C_2), 117.27 (C_4), 115.21 (C_{15},C_{17}), 113.60 (C_{12b}), 112.72(C_{7a}), 102.76 (C_{6a}), 37.23 (C_9), 33.55 (C_7), 26.85 (C_{11}), 21.26 (C_{10}); MS: (m/z, %):359 (M^+, 10), 357 (M^+-2, 85), 266 (68), 93 (68), 28 (100); Anal. Calcd. for $C_{22}H_{17}NO_4$ (359): C: 73.53, H: 4.77, N: 3.90%. Found: C: 73.48, H: 4.72, N: 3.87%.

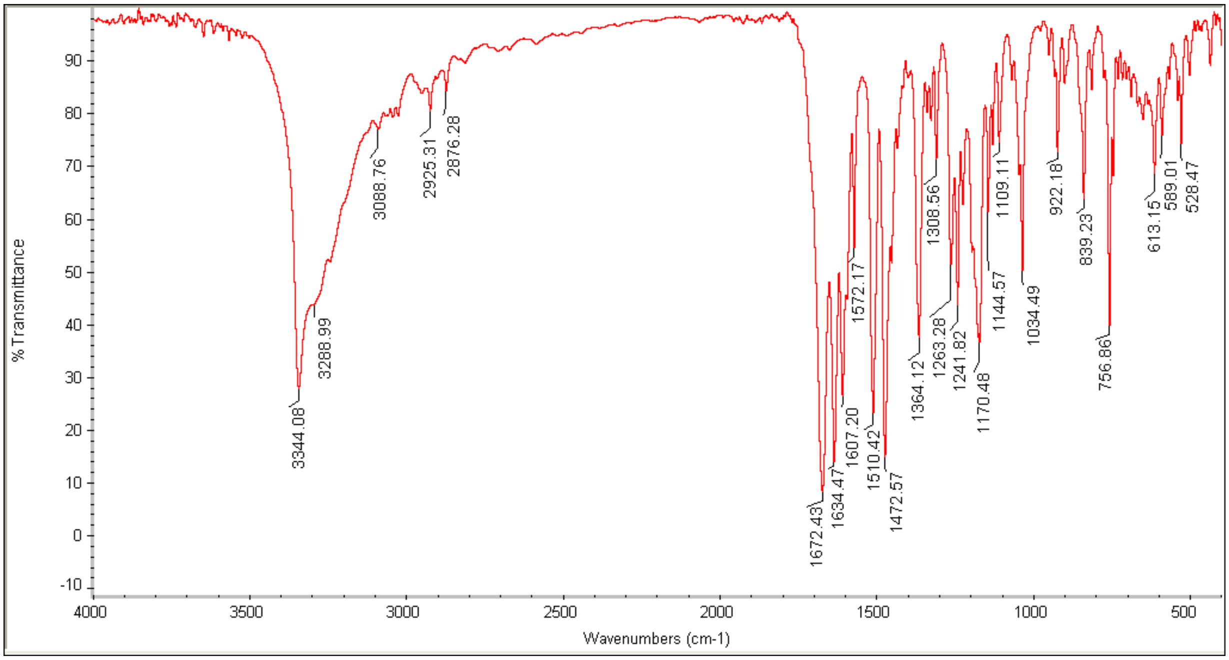

Figure 29. IR spectrum of compound **2g**

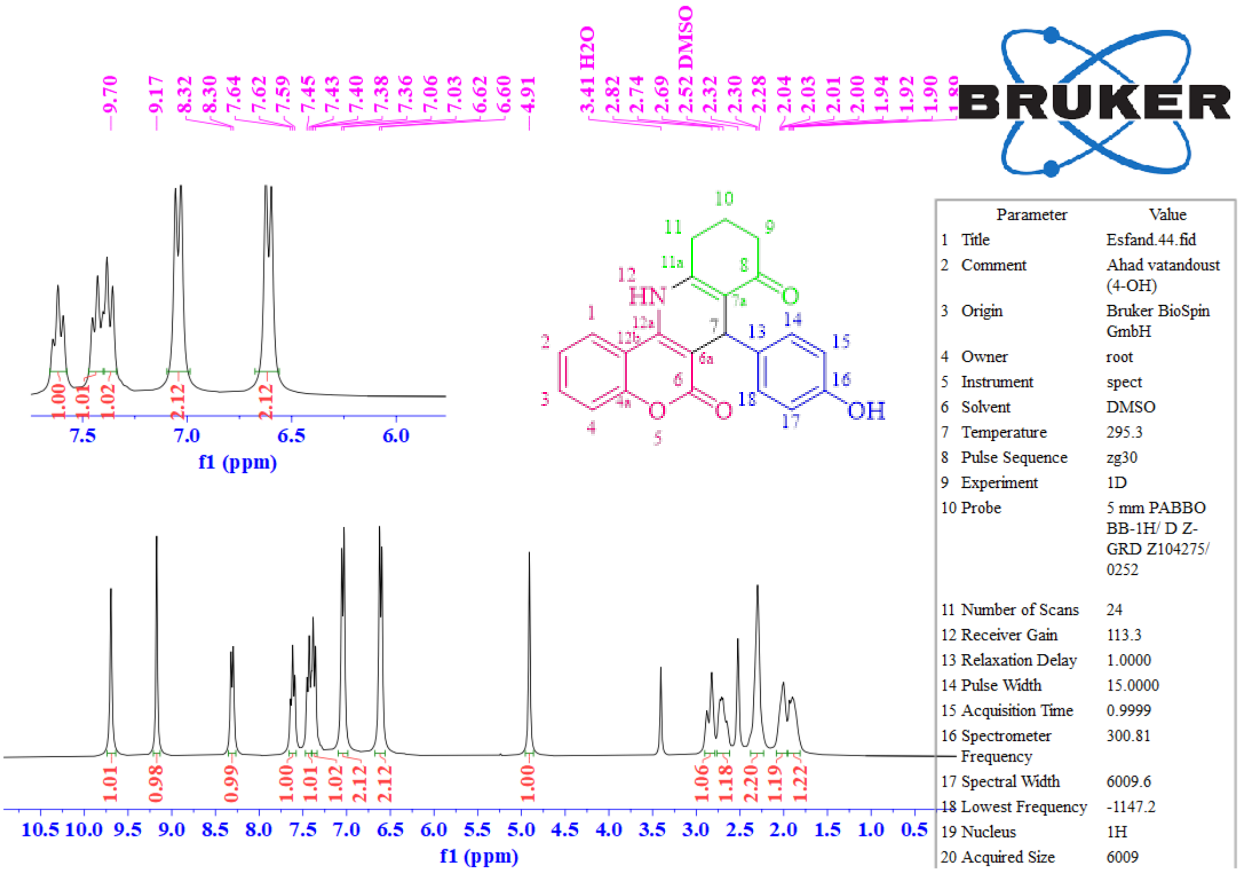

Figure 30. ^{1}H NMR (300 MHz, DMSO-d_6) spectrum of compound **2g**

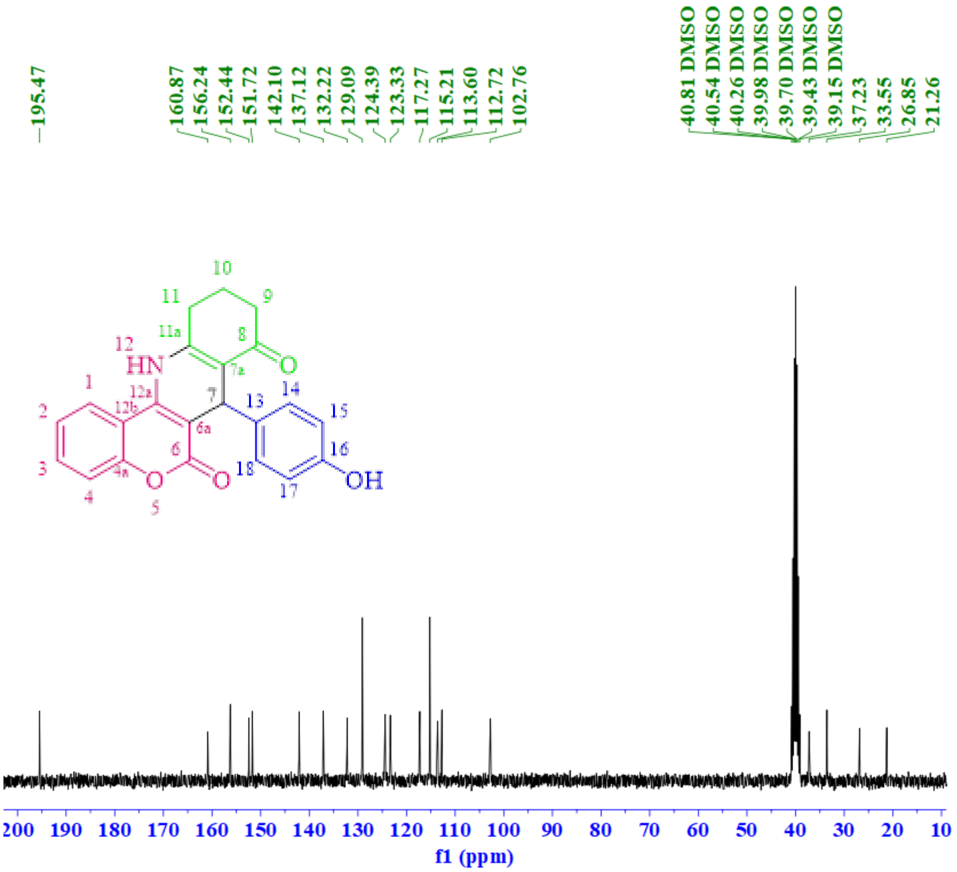

	Parameter	Value
1	Title	Esfand.45.fid
2	Comment	Ahad vatandoust (4-OH)
3	Origin	Bruker BioSpin GmbH
4	Owner	root
5	Instrument	spect
6	Solvent	DMSO
7	Temperature	295.8
8	Pulse Sequence	zgpg30
9	Experiment	1D
10	Probe	5 mm PABBO BB-1H/ D Z-GRD Z104275/ 0252
11	Number of Scans	200
12	Receiver Gain	202.0
13	Relaxation Delay	2.0000
14	Pulse Width	10.0000
15	Acquisition Time	1.8088
16	Spectrometer Frequency	75.65
17	Spectral Width	18115.9
18	Lowest Frequency	-1494.8
19	Nucleus	13C
20	Acquired Size	32768

Figure 31. ^{13}C NMR (75 MHz, DMSO-d_6) spectrum of compound **2g**

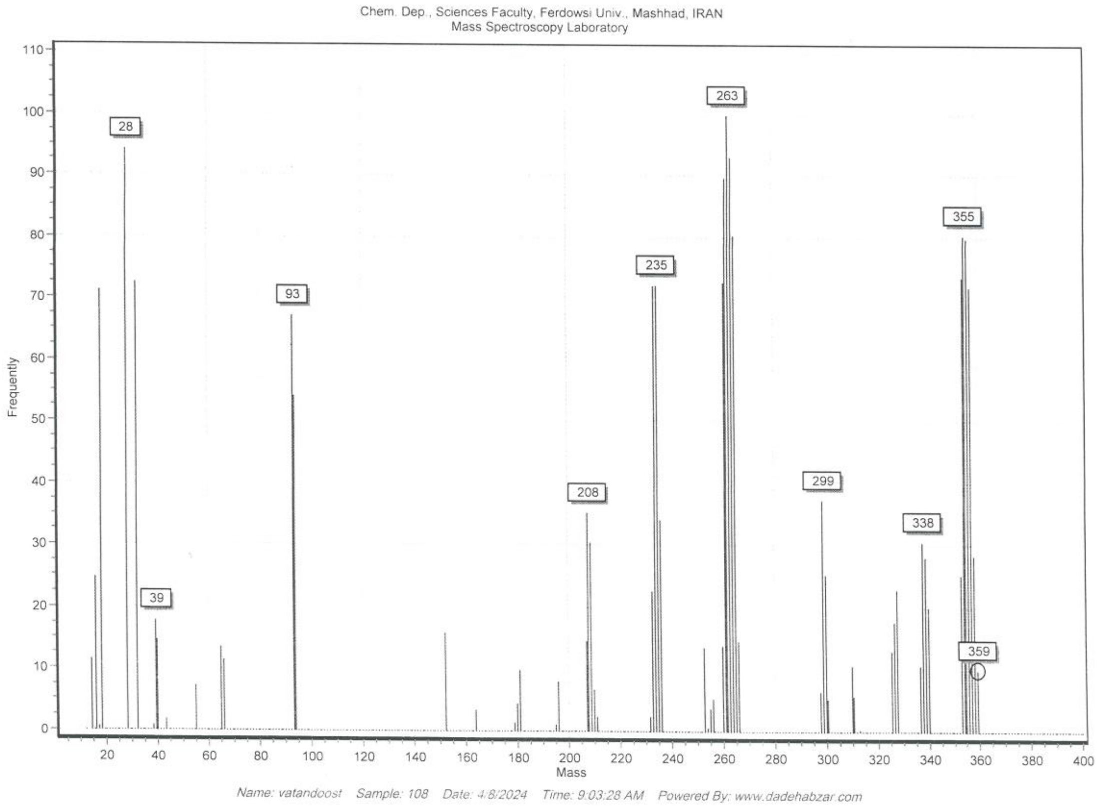

Figure 32. Mass spectrum of compound **2g**

Eager 300 Summarize Results

Date: 24/04/2024 at 13:10:23

Method Name: NCHS

Method Filename: Copy of N C H S-bkp.mth

Filename		As Method				Vial
Vatandoust-171						
# Group Sample Name		Tayp Weig. Prof. F			---	-----
171-1	108	UNK	0.643	6.25	---	-----
Component Name	Element%					
Nitrogen%	3.727560558					
Carbon%	73.43045807					
Hydrogen%	4.725502014					
Sulphur%	0					

1 Sample (s) in Group No:1

Component Name	Average
Nitrogen%	3.727560558
Carbon%	73.43045807
Hydrogen%	4.725502014
Sulphur%	0

Figure 33. CHNS spectrum of compound **2g**

Anal. Calcd. for $C_{22}H_{17}NO_4$ (359)		
C: 73.53 %	H: 4.77 %	N: 3.90 %

7-(2-hydroxyphenyl)-7,10,11,12-tetrahydro-6H-chromeno[4,3-b]quinoline-6,8(9H)-dione (2h)[4]

White solid; (0.28g, 80%); Mp=337-338 °C (Lit. 338-340 °C); IR (KBr) (υ_{max}/cm^{-1}): 3350(NH), 3199(OH), 3092, 3047(C-H aromatic), 2937, 2896 (C-H aliphatic), 1674 (C=O), 1648,1596 (C=C); ^1H NMR (300 MHz, DMSO-d_6): δ (ppm): 9.91 (s, 1H, NH), 9.33 (s, 1H, OH), 8.37 (d, J = 8.2 Hz, 1H, ArH, H_1), 7.66 (t, J = 7.9 Hz, 1H, ArH, H_3), 7.48 (t, J = 7.7 Hz, 1H, ArH, H_2), 7.42 (d, J = 7.6 Hz, 1H, ArH, H_4), 7.02-6.95 (m, 2H, ArH, H_{16}, H_{18}), 6.70 (d, J = 7.7 Hz, 2H ArH, H_{15}, H_{17}), 5.05 (s, 1H, CH, H_7), 2.86-2.80 (m, 1H, CH_2, H_9), 2.73-2.67 (m, 1H, CH_2, H_9), 2.37-2.27 (m, 2H, CH_2, H_{11}), 2.03-1.93 (m, 1H, CH_2, H_{10}), 1.88-1.79 (s, 1H, CH_2, H_{10}); ^{13}C NMR (75 MHz, DMSO-d_6): δ (ppm): 195.62 (C_8), 160.82 (C_6), 154.92 (C_{14}), 153.60 (C_{4a}), 152.47 (C_{11a}), 140.21 (C_{12a}), 132.59 (C_3), 132.55 (C_{13}), 129.99 (C_{18}), 127.89 (C_{16}), 124.53 (C_1), 123.02 (C_2), 119.83 (C_{17}), 117.57 (C_4), 117.17(C_{15}), 113.31 (C_{12b}), 111.75(C_{7a}), 101.58 (C_{6a}), 36.58 (C_9), 31.09 (C_7), 27.14 (C_{11}), 21.19 (C_{10}); MS: (m/z, %): 359 (M^+, 78), 357 (M^+-2, 38), 266 (42), 93 (56), 28 (100); Anal. Calcd. for $C_{22}H_{17}NO_4$ (359): C: 73.53, H: 4.77, N: 3.90%. Found: C: 73.51, H: 4.74, N: 3.88%.

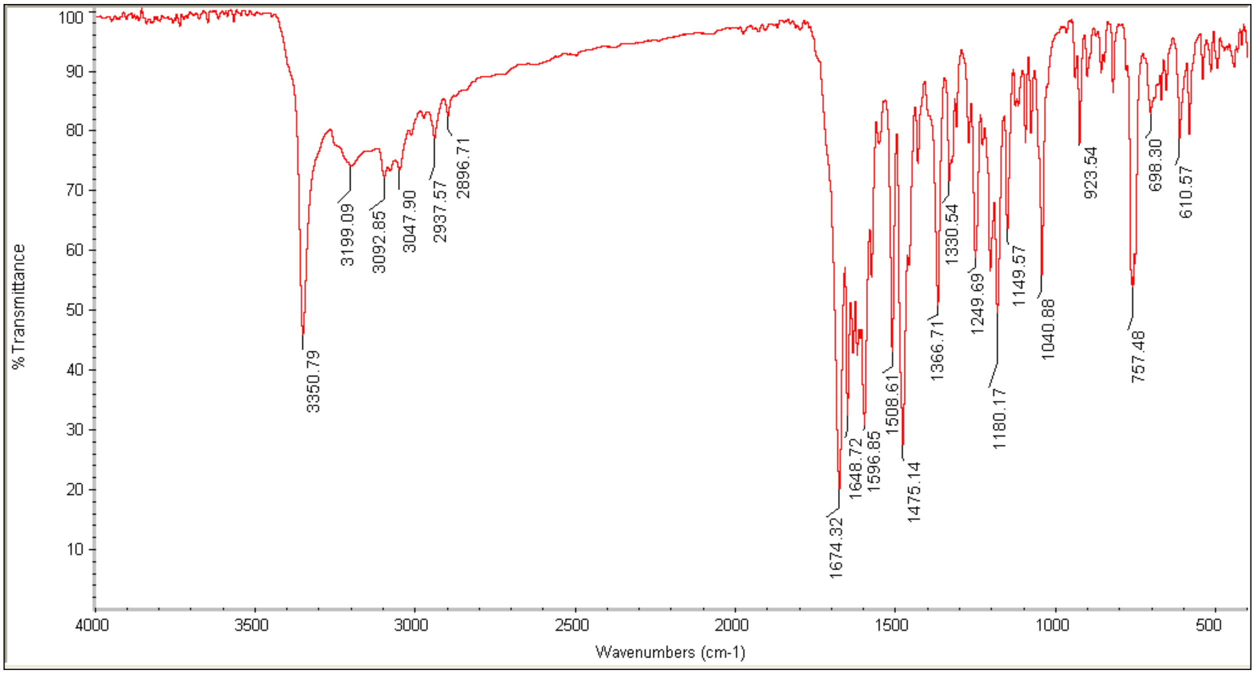

Figure 34. IR spectrum of compound **2h**

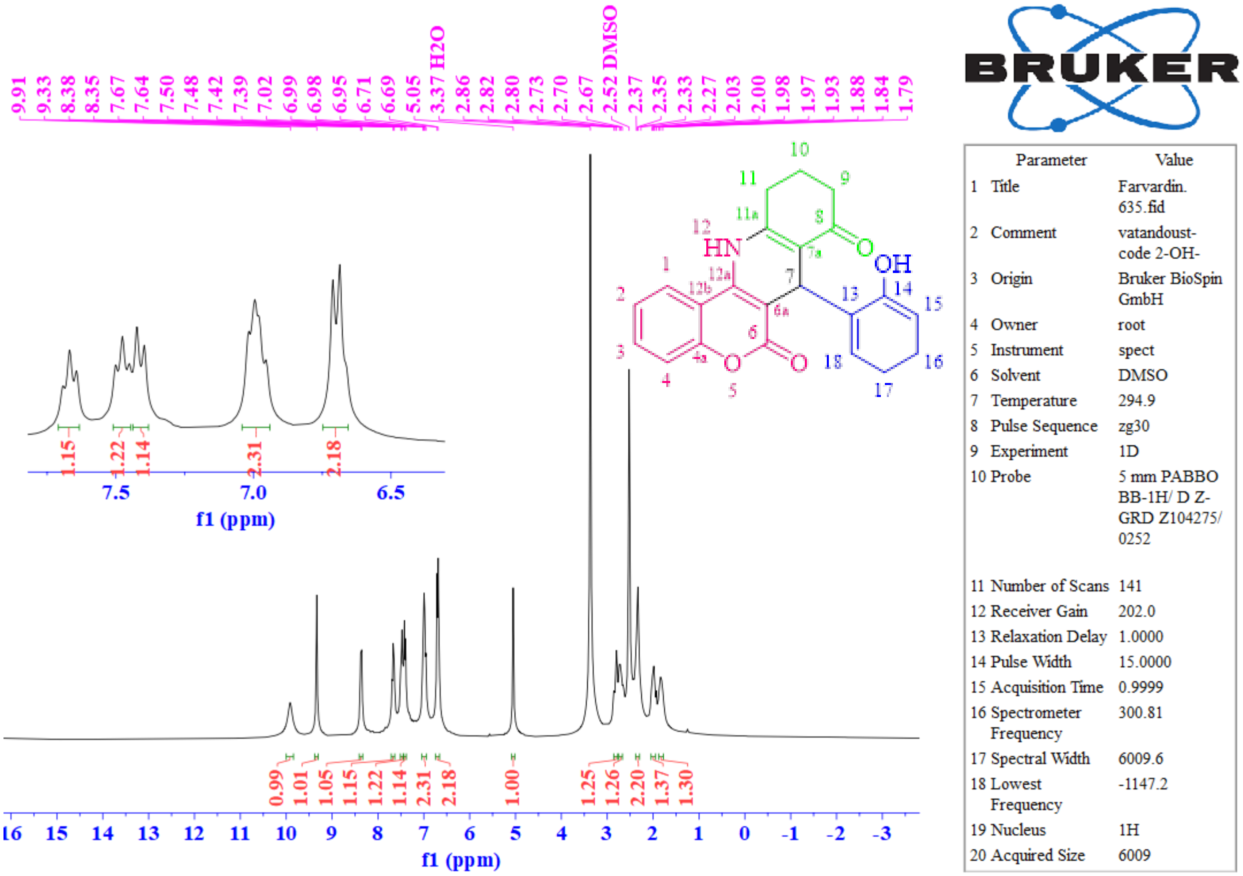

Figure 35. ^{1}H NMR (300 MHz, DMSO-d_6) spectrum of compound **2h**

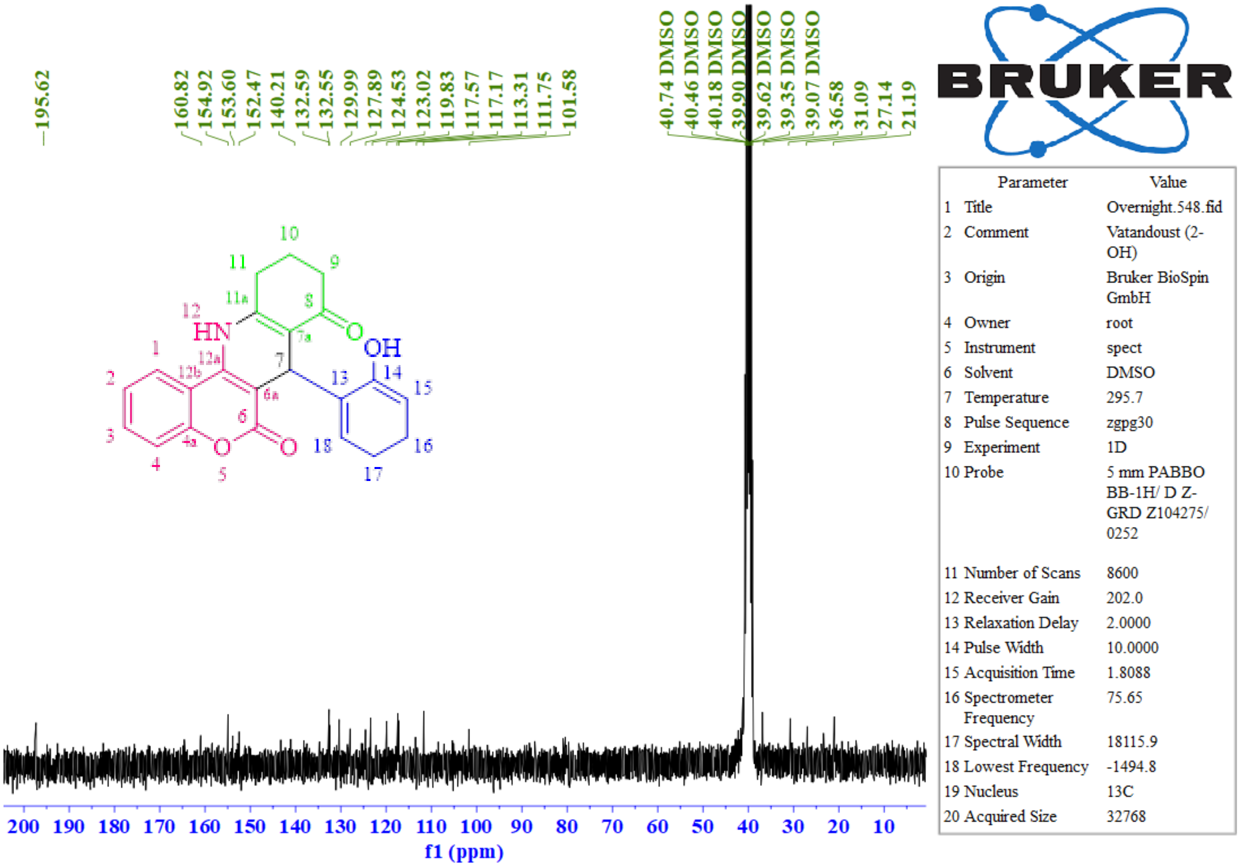

Figure 36. ^{13}C NMR (75 MHz, DMSO-d_6) spectrum of compound **2h**

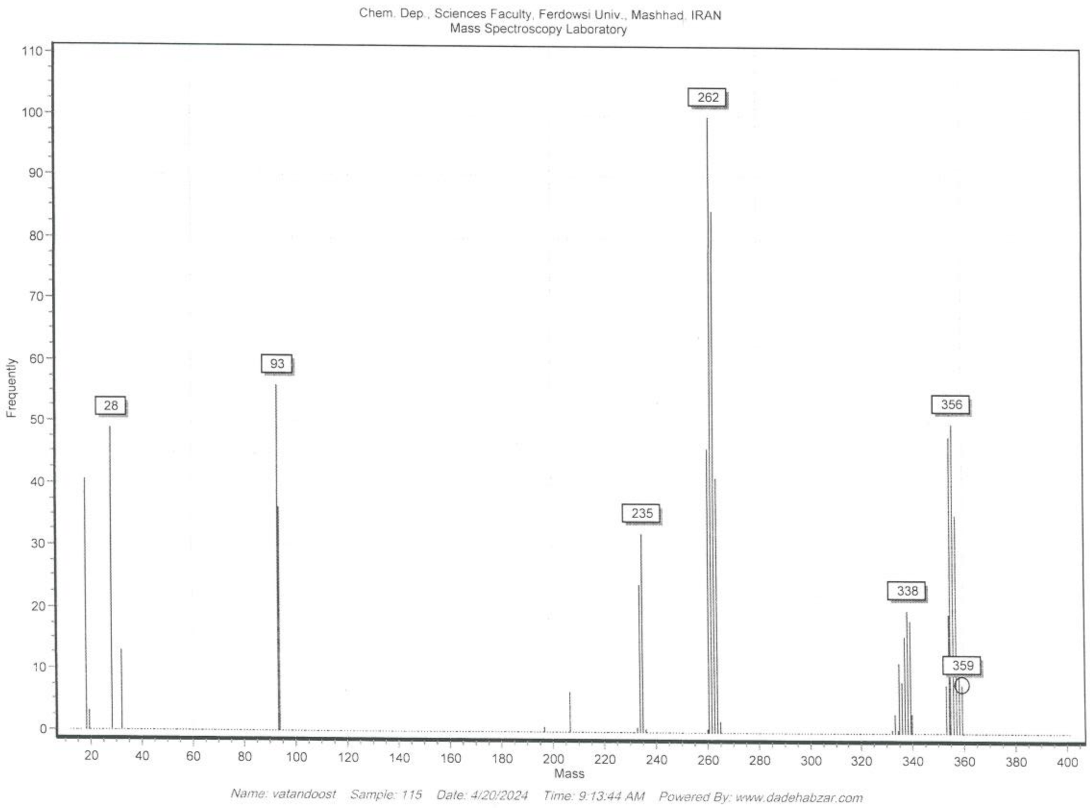

Figure 37. Mass spectrum of compound **2h**

Eager 300 Summarize Results

Date: 24/04/2024 at 13:02:47

Method Name: NCHS

Method Filename: Copy of N C H S-bkp.mth

Filename		As Method				Vial
Vatandoust-163						
# Group Sample Name		Tayp Weig. Prof. F			---	-----
163-1	115	UNK	0.591	6.25	---	-----
Component Name	Element%					

Nitrogen%	3.889078617
Carbon%	73.51745331
Hydrogen%	4.744987651
Sulphur%	0

1 Sample (s) in Group No:1

Component Name	Average
Nitrogen%	3.889078617
Carbon%	73.51745331
Hydrogen%	4.744987651
Sulphur%	0

Figure 38. CHNS spectrum of compound **2h**

Anal. Calcd. for $C_{22}H_{17}NO_4$ (359)		
C: 73.53 %	H: 4.77 %	N: 3.90 %

7-(4-methoxyphenyl)-7,10,11,12-tetrahydro-6H-chromeno[4,3-b]quinoline-6,8(9H)-dione (2i)[2]

Light yellow solid; (0.298g, 80%); Mp=274-275 °C (Lit 276-278 °C); IR (KBr) (υ_{max}/cm^{-1}): 3325(NH), 3087, 3039(C-H aromatic), 2947, 2839 (C-H aliphatic),1669 (C=O), 1605 (C=C); MS: (m/z, %): 373 (M$^+$, 22), 371 (M$^+$-2, 100), 343 (5) 266 (56), 28 (100).

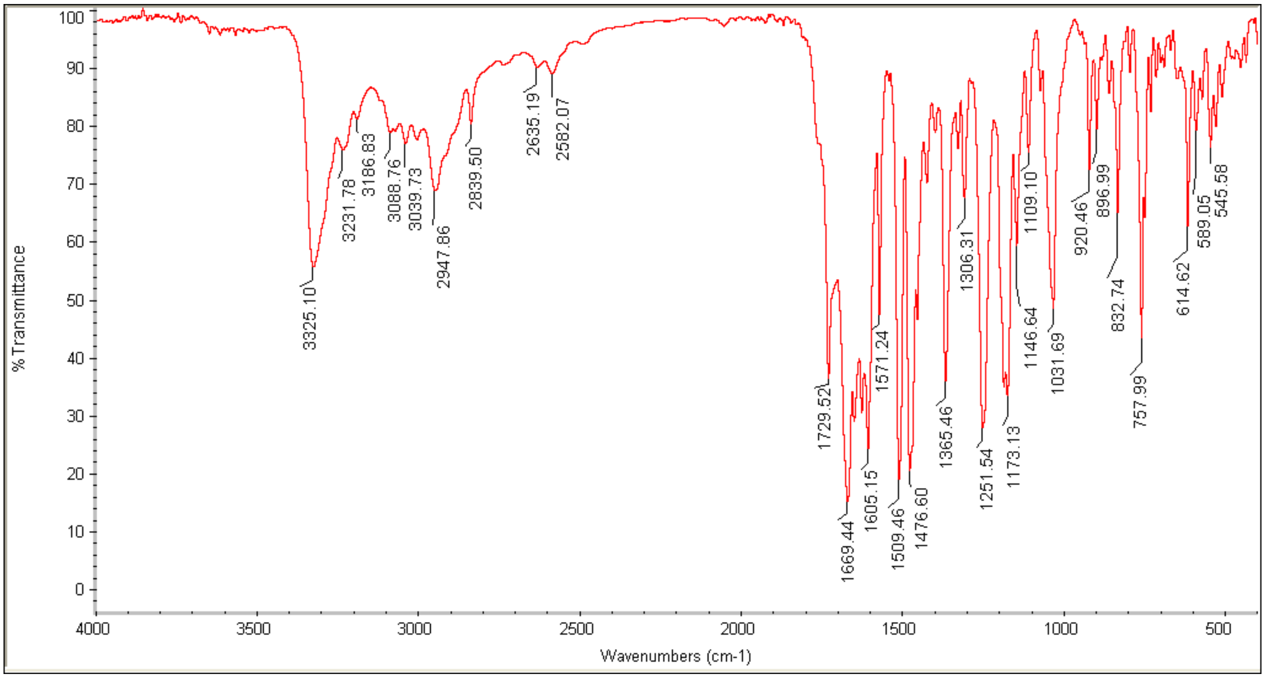

Figure 39. IR spectrum of compound **2i**

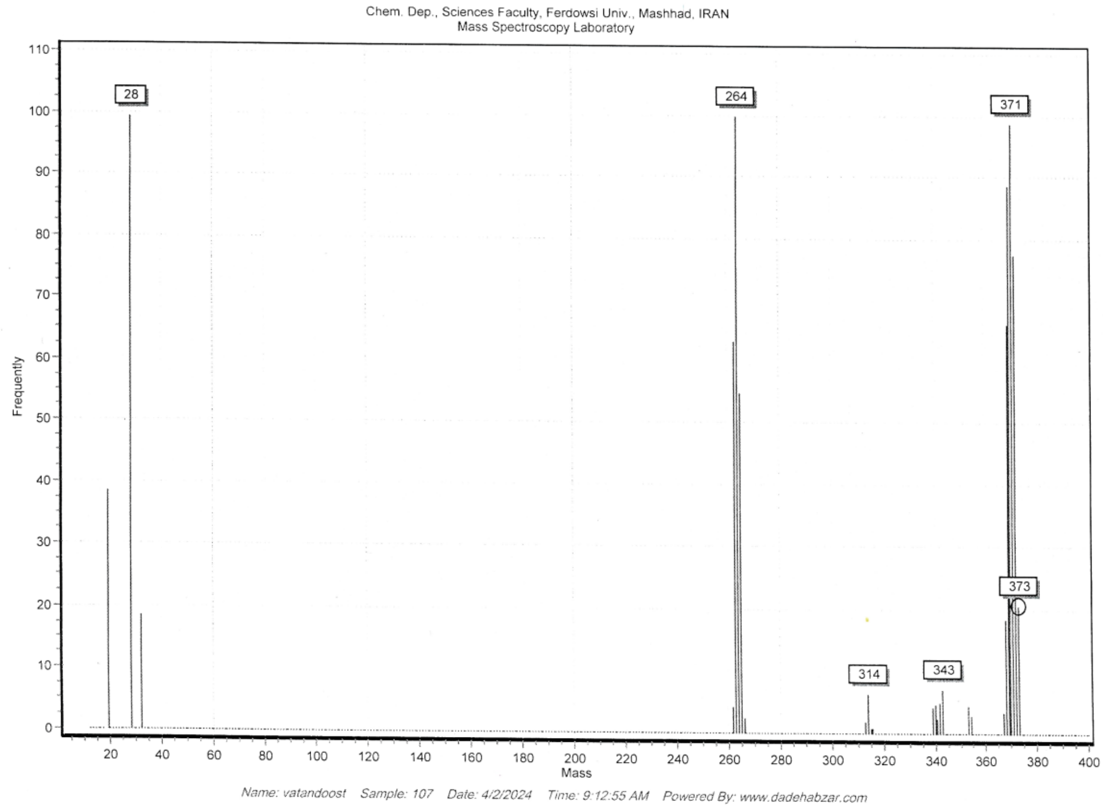

Figure 40. Mass spectrum of compound **2i**

7-(p-tolyl)-7,10,11,12-tetrahydro-6H-chromeno[4,3-b]quinoline-6,8(9H)-dione (2j)[2]

Light yellow solid; (0.285g, 80%); Mp=302-303 °C (Lit. 304-305 °C); IR (KBr) (υ_{max}/cm^{-1}): 3308(NH), 3088 (C-H aromatic), 2953, 2896 (C-H aliphatic), 1665 (C=O), 1634,1605 (C=C); ^{1}H NMR (300 MHz, DMSO-d_6): δ (ppm): 9.71 (s,1H, NH), 8.31 (d, J = 7.9 Hz, 1H, ArH, H_1), 7.65 (t, J = 7.7 Hz, 1H, ArH, H_3), 7.43 (m, 2H, ArH, H_2, H_4), 7.13 (d, J = 8.2 Hz, 2H, ArH, H_{14}, H_{18}), 7.02 (d, J = 8.2 Hz, 2H, ArH, H_{15}, H_{17}), 4.97 (s, 1H, CH, H_7), 2.87-2.78 (m, 1H, CH_2, H_9) 2.75-2.65 (m, 1H, CH_2, H_9), 2.35-2.24 (m, 2H, CH_2, H_{11}), 2.20 (s, 3H, CH_3), 2.06-1.92 (m, 1H, CH_2, H_{10}), 1.89-1.83 (m, 1H, CH_2, H_{10}); ^{13}C NMR (75 MHz, DMSO-d_6): δ (ppm): 195.41 (C_8), 160.81 (C_6), 152.49 (C_{4a}), 151.94 (C_{11a}), 143.55 (C_{12a}), 142.38 (C_{13}), 135.68 (C_{16}), 132.35(C_3), 129.05 (C_{15},C_{17}), 128.03 (C_{14},C_{18}), 124.44 (C_1), 123.39 (C_2), 117.31(C_4), 113.76 (C_{12b}), 112.50 (C_{7a}), 102.42 (C_{6a}), 37.16(C_9), 34.17 (C_7), 26.83 (C_{11}), 21.22 (C_{10}), 21.05 (CH_3); MS: (m/z, %): 357 (M^+, 10), 355 (M^+-2, 40), 266 (60), 152 (88), 91 (28), 28 (100); Anal. Calcd. for $C_{23}H_{19}NO_3$ (357): C: 77.29, H: 5.36, N: 3.92%. Found: C: 77.12, H: 5.22, N: 3.78%.

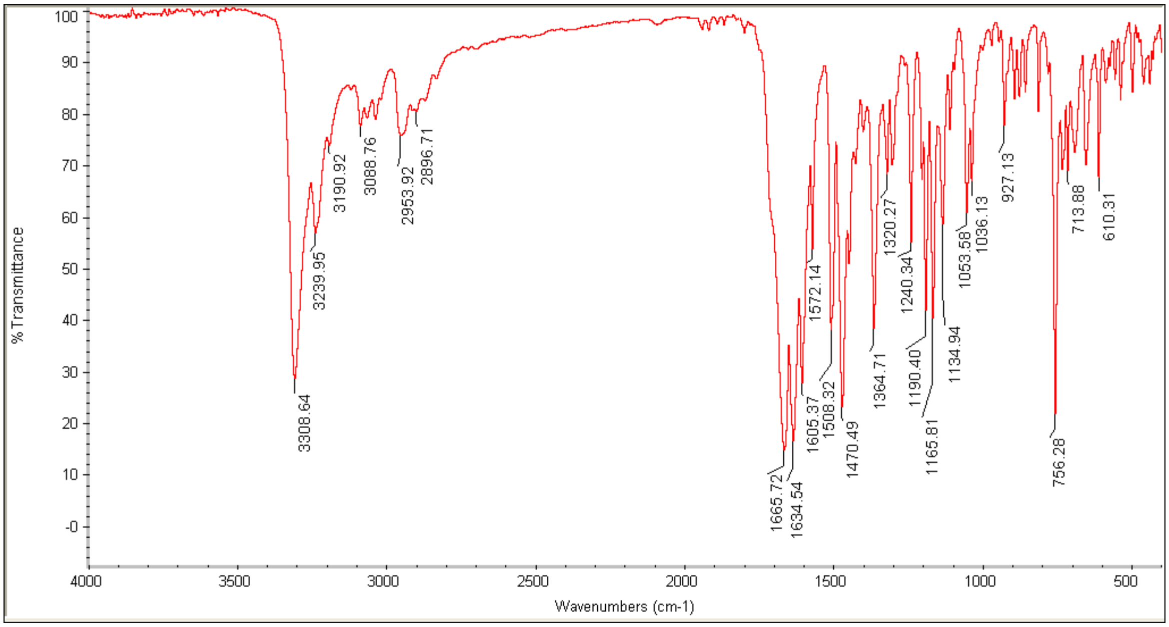

Figure 41. IR spectrum of compound **2j**

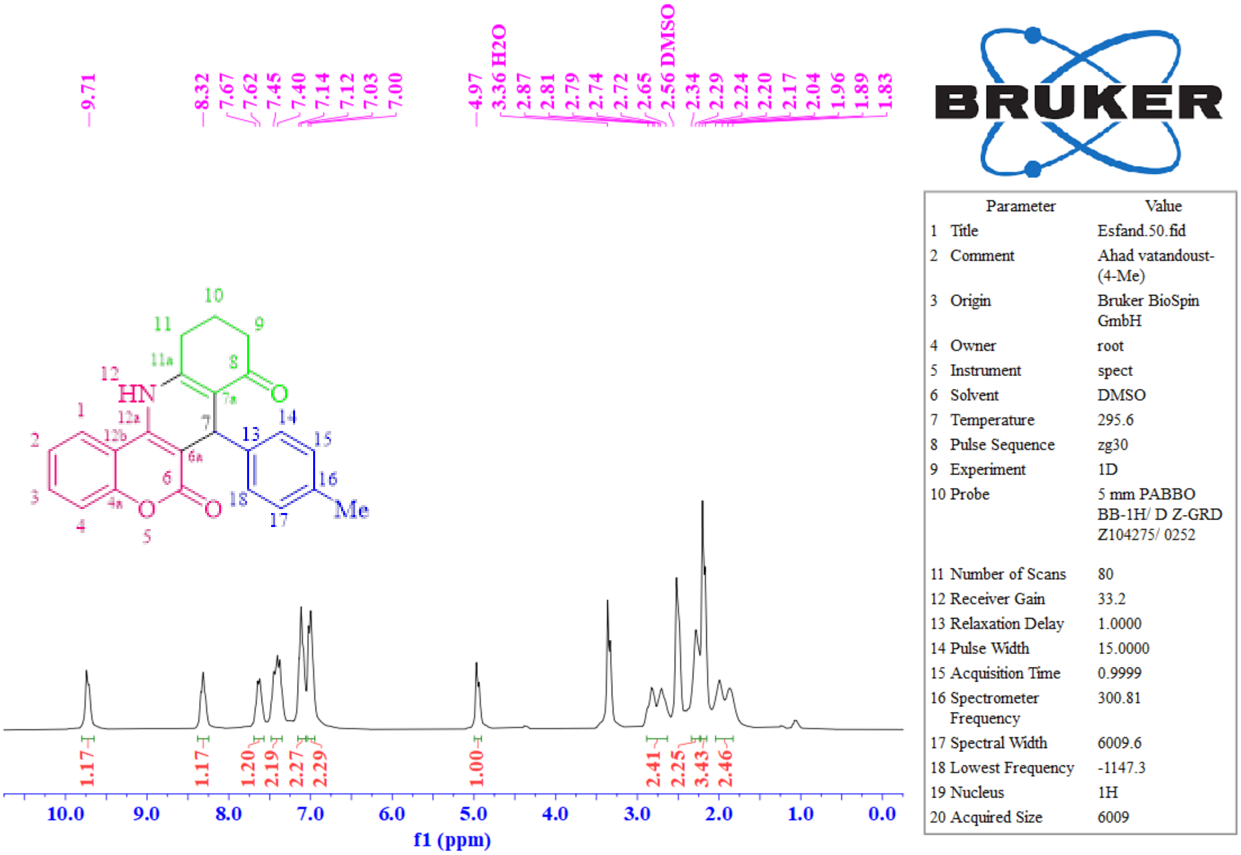

Figure 42. ^{1}H NMR (300 MHz, DMSO-d_6) spectrum of compound **2j**

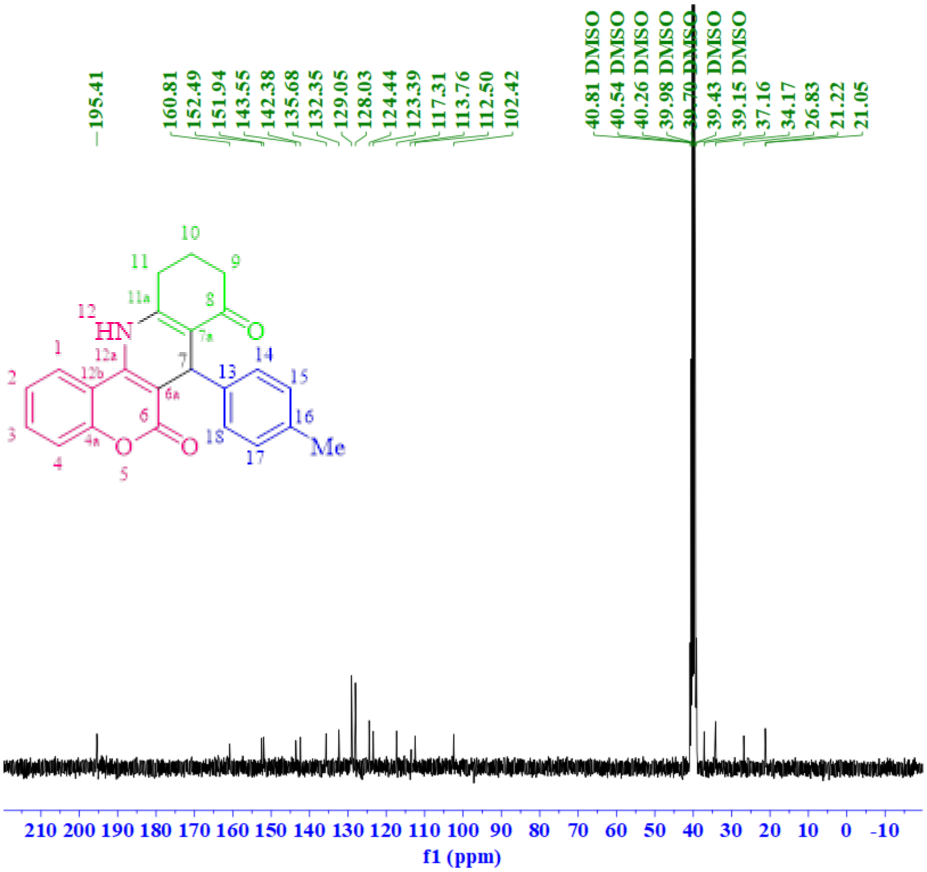

	Parameter	Value
1	Title	Esfand.60.fid
2	Comment	Ahad vatandoust (4-Me)
3	Origin	Bruker BioSpin GmbH
4	Owner	root
5	Instrument	spect
6	Solvent	DMSO
7	Temperature	296.3
8	Pulse Sequence	zgpg30
9	Experiment	1D
10	Probe	5 mm PABBO BB-1H/ D Z-GRD Z104275/ 0252
11	Number of Scans	1024
12	Receiver Gain	202.0
13	Relaxation Delay	2.0000
14	Pulse Width	10.0000
15	Acquisition Time	1.8088
16	Spectrometer Frequency	75.65
17	Spectral Width	18115.9
18	Lowest Frequency	-1494.1
19	Nucleus	13C
20	Acquired Size	32768

Figure 43. ^{13}C NMR (75 MHz, DMSO-d_6) spectrum of compound **2j**

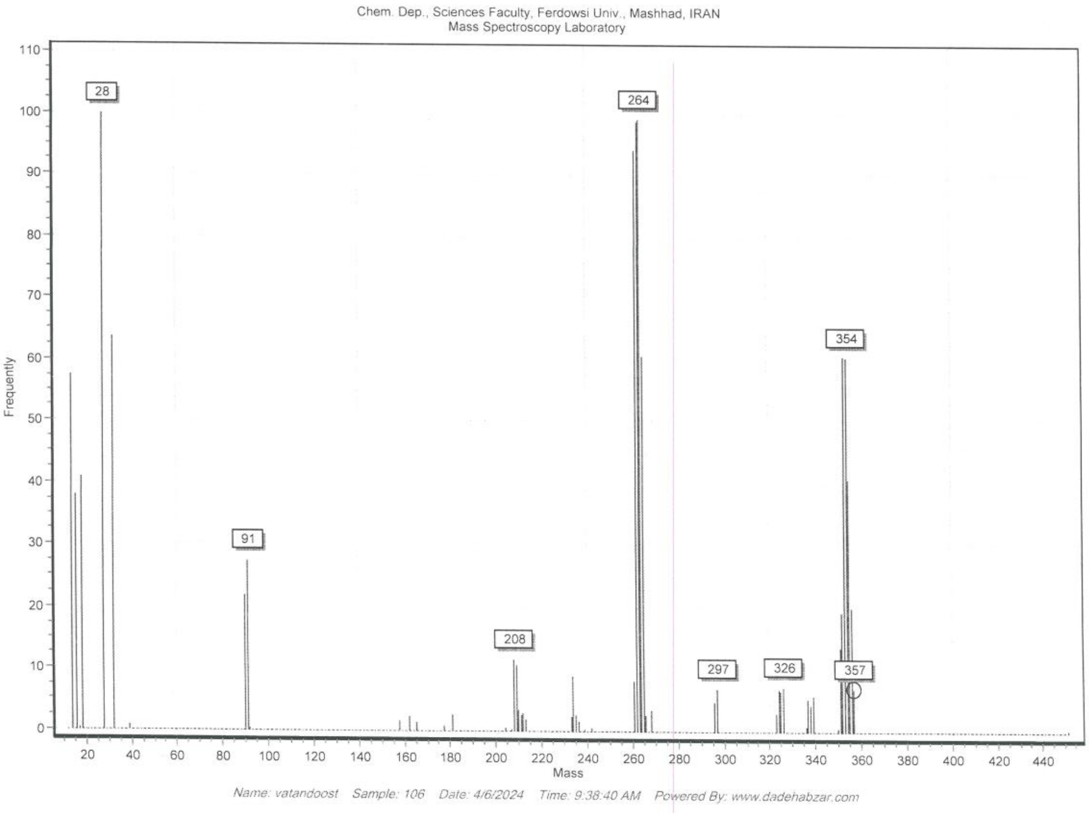

Figure 44. Mass spectrum of compound **2j**

Eager 300 Summarize Results

Date: 24/04/2024 at 13:04:57

Method Name: NCHS

Method Filename: Copy of N C H S-bkp.mth

Filename		As Method				Vial
Vatandoust-173						
# Group Sample Name		Tayp	Weig.	Prof. F	---	-----
173-1	106	UNK	0.651	6.25	---	-----
Component Name	Element%					
Nitrogen%	3.782251453					
Carbon%	77.1215451					
Hydrogen%	5.224343908					
Sulphur%	0					

1 Sample (s) in Group No:1

Component Name	Average
Nitrogen%	3.782251453
Carbon%	77.1215451
Hydrogen%	5.224343908
Sulphur%	0

Figure 45. CHNS spectrum of compound **2j**

Anal. Calcd. for $C_{23}H_{19}NO_3$ (357)		
C: 77.29 %	H: 5.36 %	N: 3.92 %

7-(m-tolyl)-7,10,11,12-tetrahydro-6H-chromeno[4,3-b]quinoline-6,8(9H)-dione (2k)[5]

Light yellow solid; (0.267g, 75%); Mp=309-311 °C (Lit. 310 °C); IR (KBr) (υ_{max}/cm^{-1}): 3308(NH), 3084,3035 (C-H aromatic), 2945 (C-H aliphatic), 1665 (C=O), 1634,1605 (C=C); ^{1}H NMR (300 MHz, DMSO-d_6): δ (ppm): 9.76 (s, 1H, D_2O exchangeable, NH), 8.33 (d, J = 7.9 Hz, 1H, ArH, H_1), 7.65 (t, J = 8.0 Hz, 1H, ArH, H_3), 7.45 (t, J = 7.7 Hz, 1H, ArH, H_2), 7.39 (d, J = 8.3 Hz, 1H, ArH, H_4), 7.12-7.01 (m, 3H, ArH, H_{14}, H_{17}, H_{18}), 6.93 (s, 1H, ArH, H_{16}), 4.98 (s, 1H, CH, H_7), 2.90-2.82 (m, 1H, CH_2, H_9), 2.74-2.70 (m, 1H, CH_2, H_9), 2.32-2.28 (m, 2H, CH_2, H_{11}), 2.23 (s, 3H, CH_3), 2.06-2.00 (m, 1H, CH_2, H_{10}), 1.93-1.90 (m, 1H, CH_2, H_{10}); ^{13}C NMR (75 MHz, DMSO-d_6): δ (ppm): 195.43 (C_8), 160.82 (C_6), 152.49 (C_{4a}), 152.08(C_{11a}), 146.39(C_{12a}), 142.48(C_{13}), 137.34(C_{15}), 132.39 (C_3), 128.80 (C_{17}), 128.49(C_{16}), 127.40 (C_{14}), 125.29 (C_1), 124.47 (C_{18}), 123.43 (C_2), 117.34 (C_4), 113.53 (C_{12b}), 112.40 (C_{7a}), 102.33 (C_{6a}), 37.18 (C_9), 34.53 (C_7), 26.88 (C_{11}), 21.62 (C_{10}), 21.22 (CH_3). MS: (m/z, %): 357 (M^+, 10), 355 (M^+-2, 70), 266(50), 28 (100); Anal. Calcd. for $C_{23}H_{19}NO_3$(357): C: 77.29, H: 5.36, N: 3.92%. Found: C: 77.14, H: 5.28, N: 3.80%.

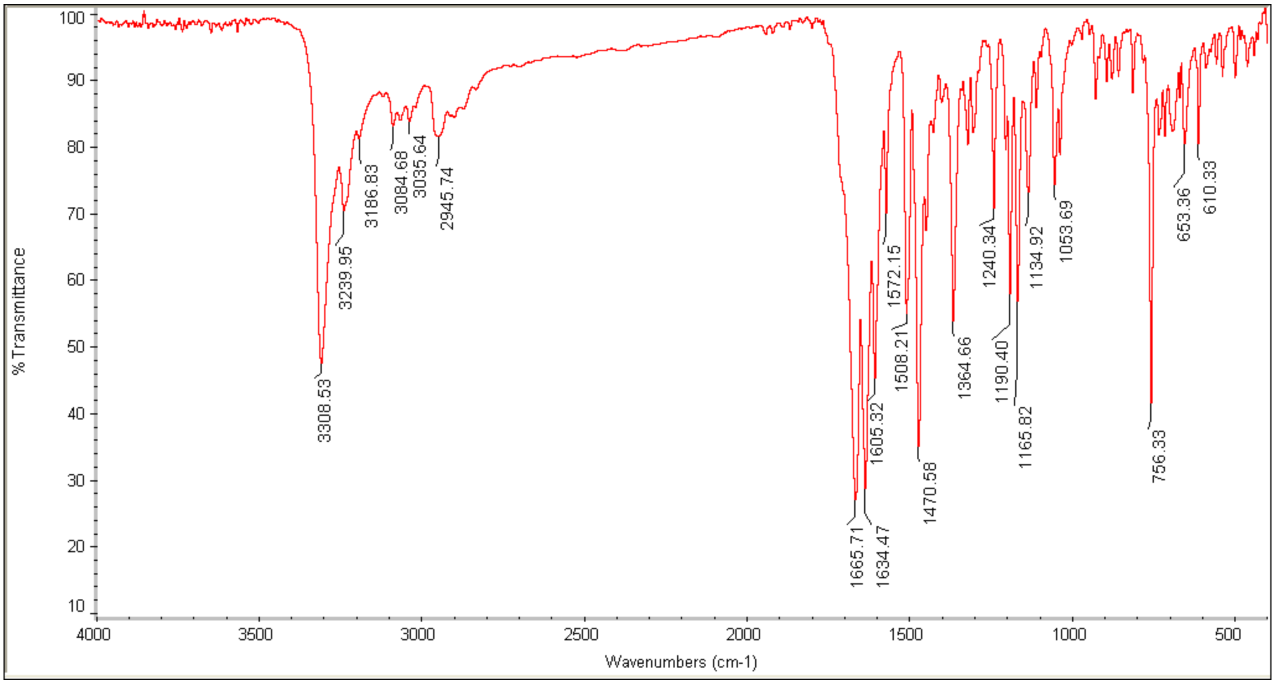

Figure 46. IR spectrum of compound **2k**

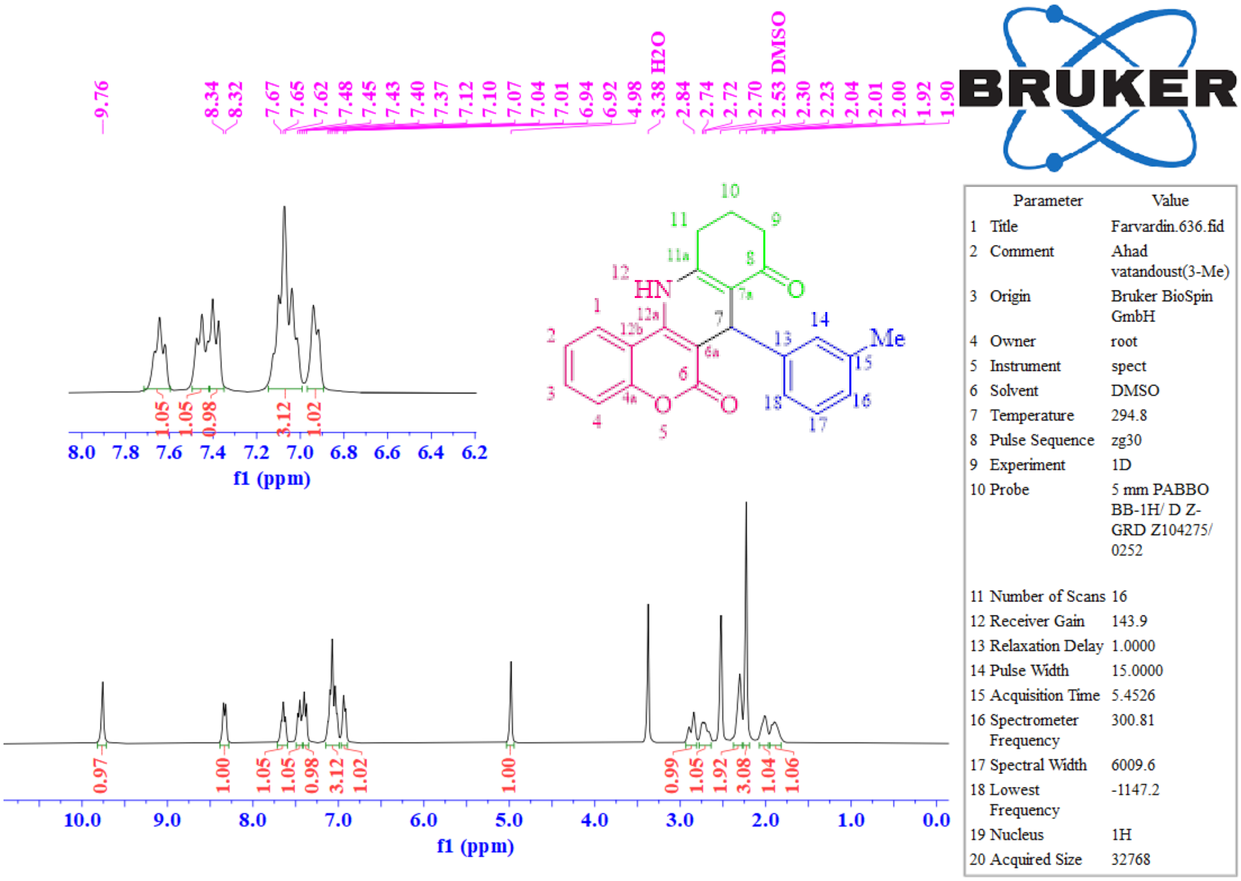

Figure 47. ^{1}H NMR (300 MHz, DMSO-d_6) spectrum of compound **2k**

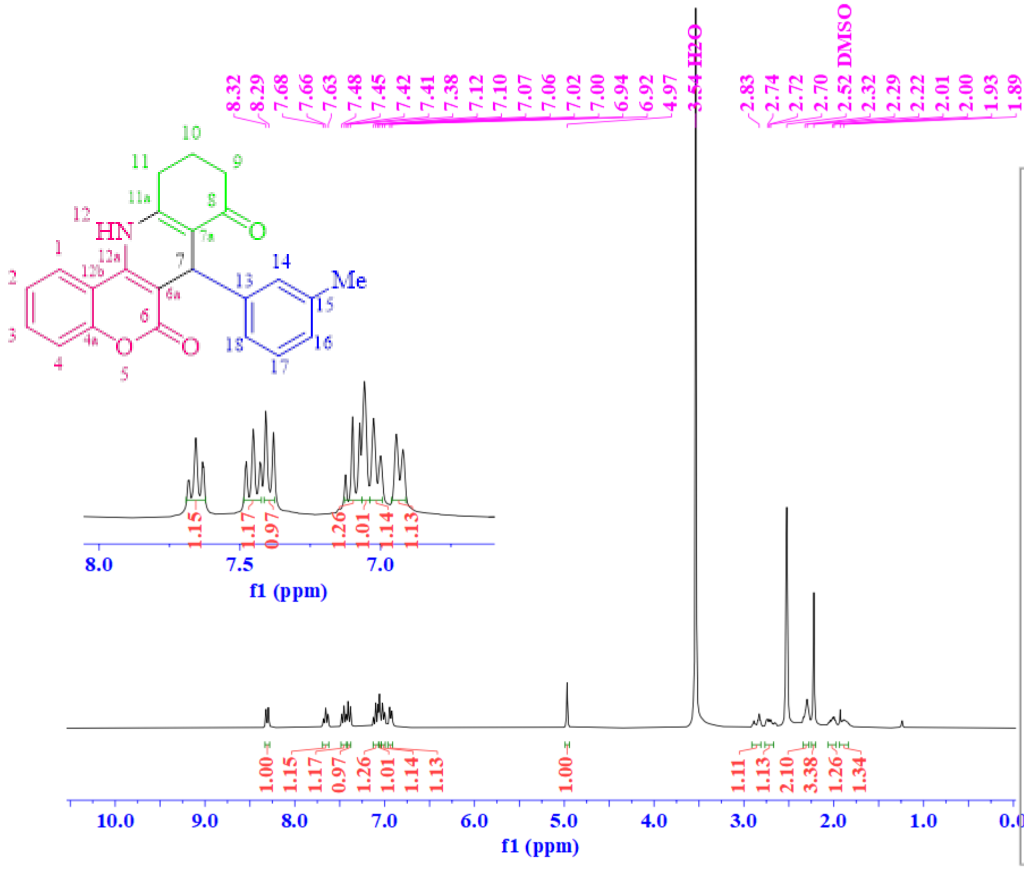

Parameter	Value
1 Title	Farvardin. 684.fid
2 Comment	Ahad vatandoust (3-Me)D2O
3 Origin	Bruker BioSpin GmbH
4 Owner	root
5 Instrument	spect
6 Solvent	DMSO
7 Temperature	296.1
8 Pulse Sequence	zg30
9 Experiment	1D
10 Probe	5 mm PABBO BB-1H/ D Z-GRD Z104275/ 0252
11 Number of Scans	512
12 Receiver Gain	202.0
13 Relaxation Delay	1.0000
14 Pulse Width	15.0000
15 Acquisition Time	0.9999
16 Spectrometer Frequency	300.81
17 Spectral Width	6009.6
18 Lowest Frequency	-1147.3
19 Nucleus	1H
20 Acquired Size	6009

Figure 48. ^{1}H NMR (300 MHz, DMSO-d_6) (D_2O) spectrum of compound **2k**

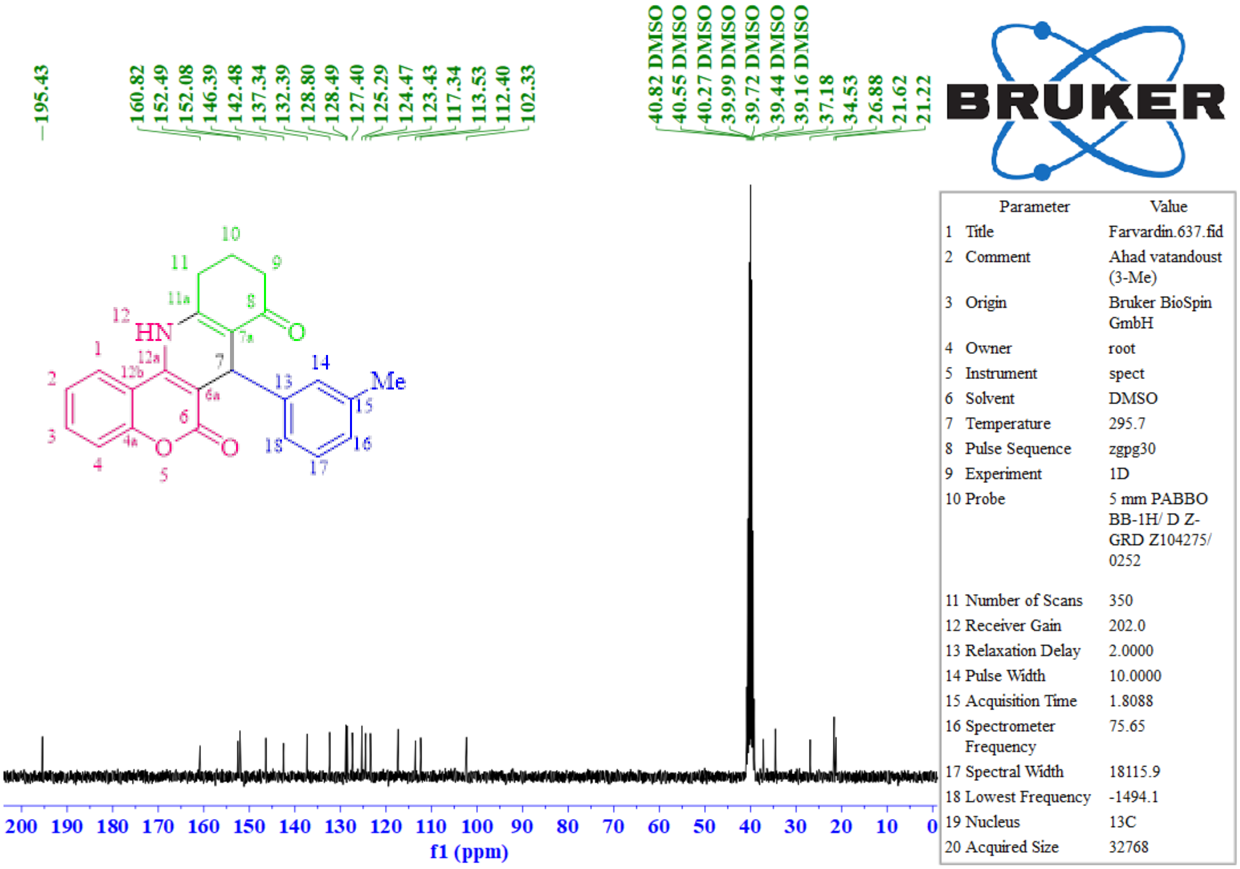

Figure 49. ^{13}C NMR (75 MHz, DMSO-d_6) spectrum of compound **2k**

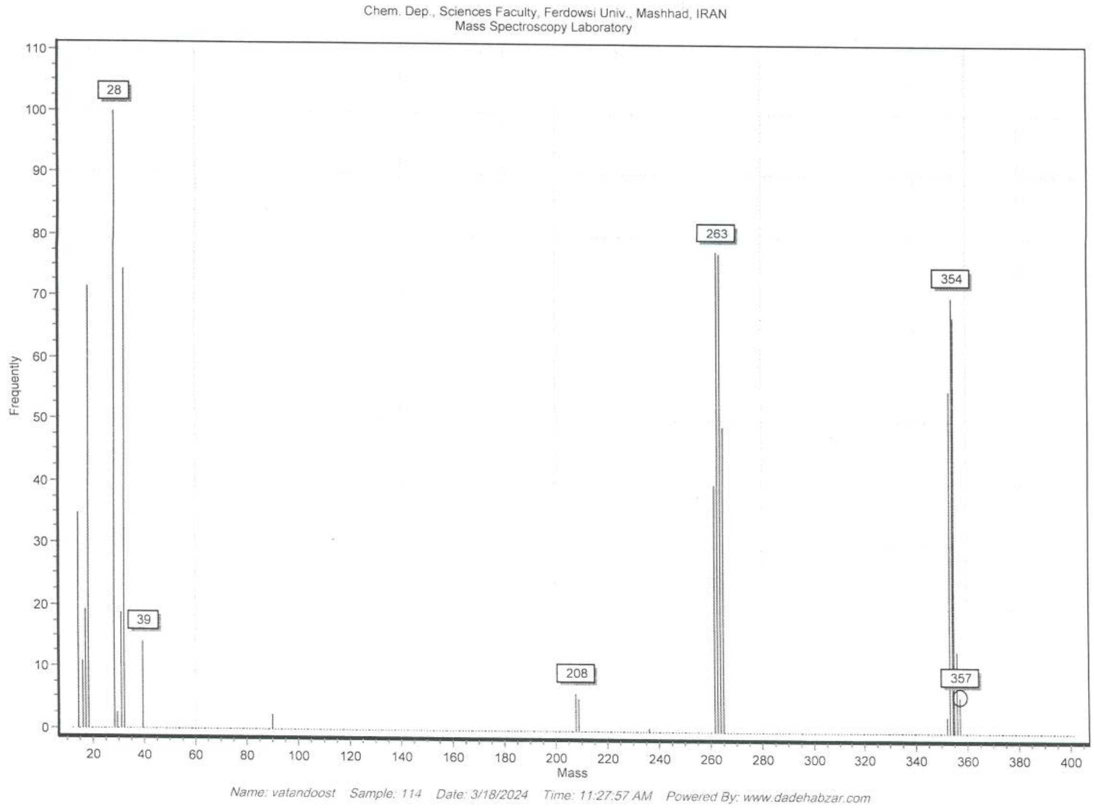

Figure 50. Mass spectrum of compound **2k**

Eager 300 Summarize Results

Date: 24/04/2024 at 13:03:23

Method Name: NCHS

Method Filename: Copy of N C H S-bkp.mth

Filename		As Method				Vial
Vatandoust-166						
# Group Sample Name		Tayp Weig. Prof. F			---	-----
166-1	114	UNK	0.607	6.25	---	-----
Component Name	Element%					
Nitrogen%	3.80542572					
Carbon%	77.14088562					
Hydrogen%	5.288564281					
Sulphur%	0					

1 Sample (s) in Group No:1

Component Name	Average
Nitrogen%	3.80542572
Carbon%	77.14088562
Hydrogen%	5.288564281
Sulphur%	0

Figure 51. CHNS spectrum of compound **2k**

Anal. Calcd. for $C_{23}H_{19}NO_3$(357)		
C: 77.29 %	H: 5.36 %	N: 3.92 %

7-(2-hydroxy-5-nitrophenyl)-7,10,11,12-tetrahydro-6H-chromeno[4,3-b]quinoline-6,8(9H)-dione (2l)

Light yellow solid; (0.343g, 85%); Mp=323-324 °C; IR (KBr) (υ_{max}/cm^{-1}): 3348 (NH), 3252 (OH) 3050 (C-H aromatic), 2941 (C-H aliphatic), 1676 (C=O), 1649, 1599 (C=C); ^{1}H NMR (300 MHz, DMSO-d_6): δ (ppm): 10.90 (s, 1H, OH), 9.82 (s, 1H, NH), 8.33 (d, J = 8.1 Hz, 1H, ArH, H_1), 8.05 (s, 1H, ArH, H_{18}), 7.92 (d, J = 9.4 Hz, 1H, ArH, H_{16}), 7.64 (t, J = 7.9 Hz, 1H, ArH, H_3), 7.45 (t, J = 7.7 Hz, 1H, ArH, H_2), 7.37 (d, J = 8.3 Hz, 1H, ArH, H_4), 6.83 (d, J = 9.0 Hz, 1H, ArH, H_{15}), 5.11 (s, 1H, CH, H_7), 2.83-2.64(m, 1H, CH_2, H_9), 2.74-2.70(m, 1H, CH_2, H_9), 2.37-2.25 (m, 2H, CH_2, H_{11}), 2.00-1.93 (m, 1H, CH_2, H_{10}), 1.88-1.78 (m, 1H, CH_2, H_{10}); ^{13}C NMR (75 MHz, DMSO-d_6): δ (ppm): 195.89 (C_8), 162.70 (C_6), 160.64 (C_{14}), 153.35 (C_{4a}), 152.59 (C_{11a}), 143.50 (C_{12a}), 139.55 (C_{17}), 132.45 (C_3), 131.95 (C_{13}), 127.56 (C_1), 124.45 (C_{18}), 124.30 (C_{16}), 123.36 (C_2), 117.35 (C_{15}), 116.87 (C_4), 113.29 (C_{12b}), 110.28 (C_{7a}), 100.16 (C_{6a}), 37.02 (C_9), 33.49 (C_7), 26.88 (C_{11}), 21.18 (C_{10}); MS: (m/z, %):404 (M^+, 8), 402 (M^+-2, 10), 387(10), 266(10),138(35) 28(68); Anal. Calcd. for $C_{22}H_{16}N_2O_4$ (404): C: 65.35, H: 3.99, N: 6.92%. Found: C: 65.30, H: 3.92, N: 6.88%.

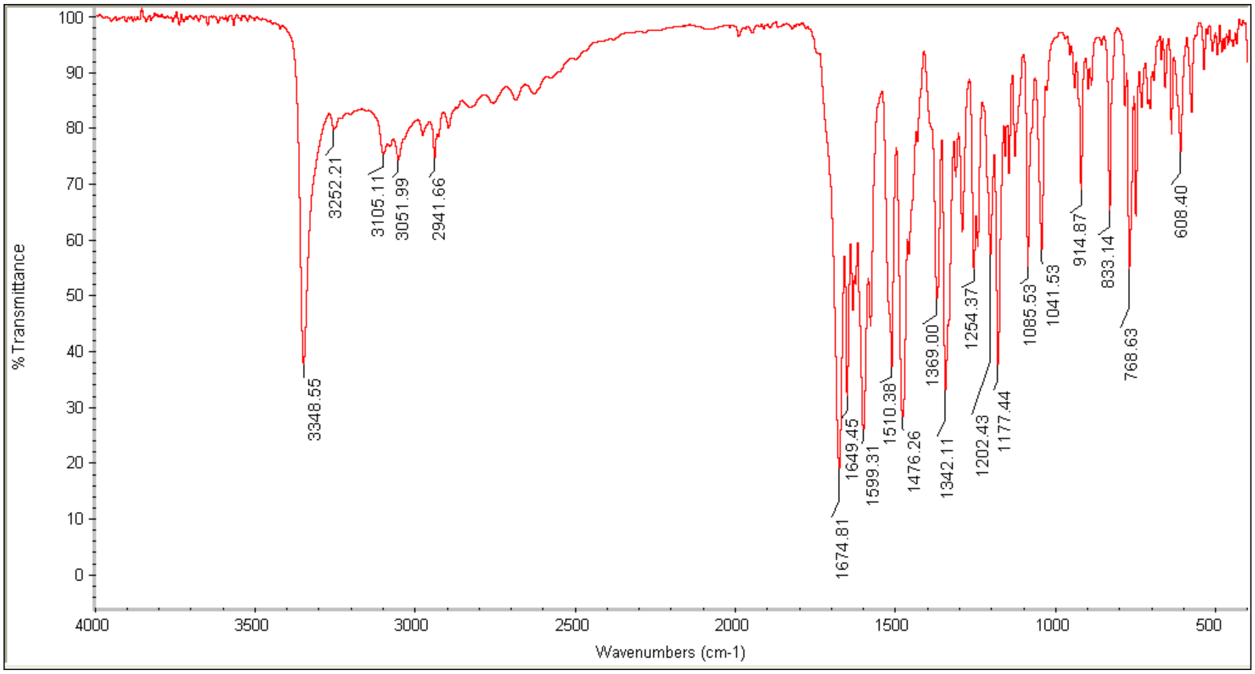

Figure 52. IR spectrum of compound **2l**

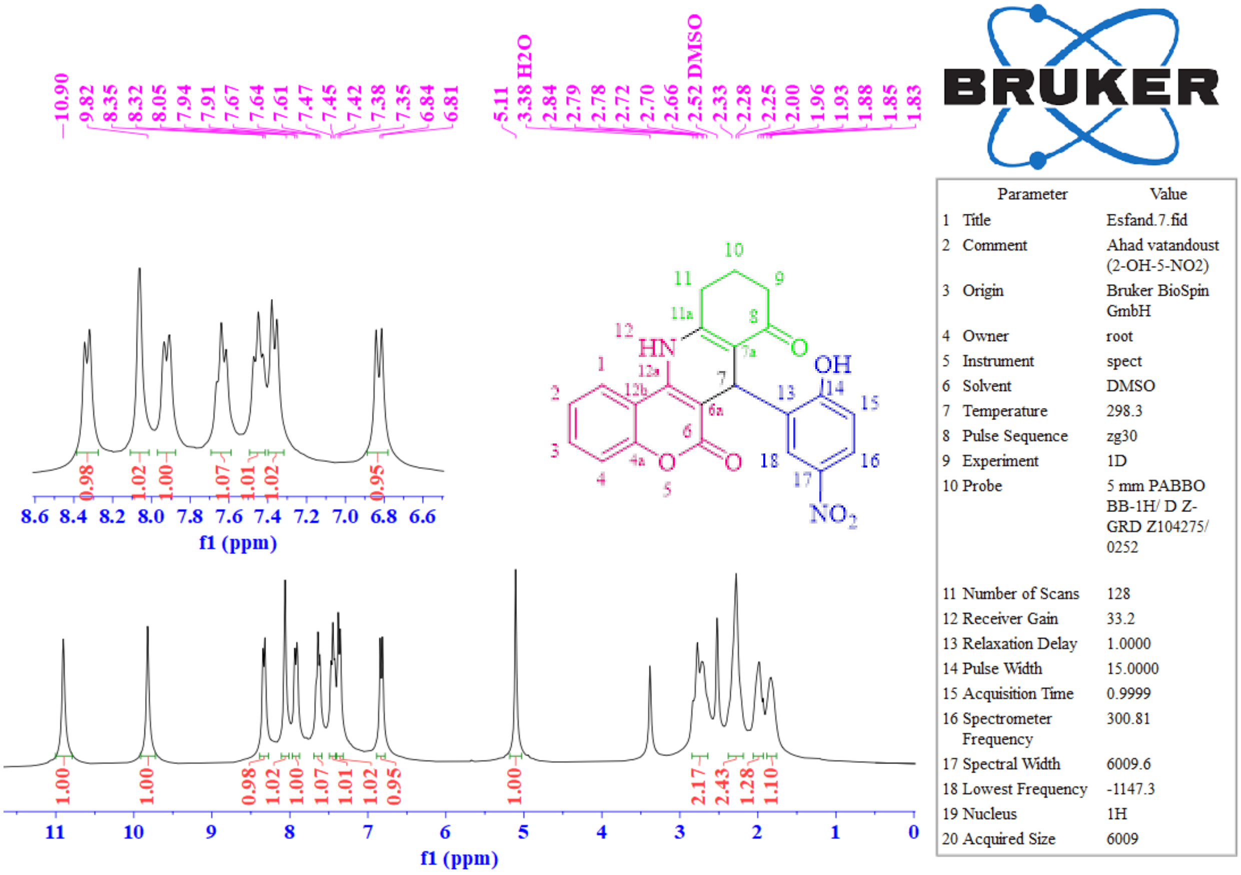

Figure 53. ^{1}H NMR (300 MHz, DMSO-d_6) spectra of compound **2l**

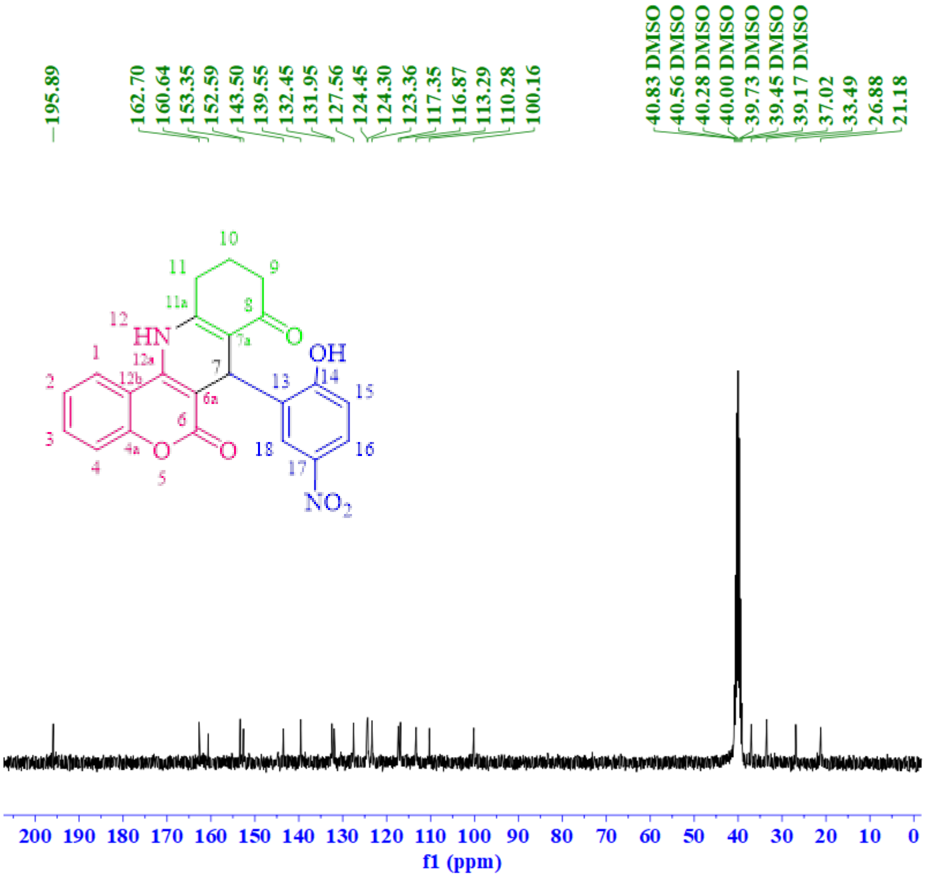

	Parameter	Value
1	Title	Esfand.8.fid
2	Comment	Ahad vatandoust (2-OH-5-NO2)
3	Origin	Bruker BioSpin GmbH
4	Owner	root
5	Instrument	spect
6	Solvent	DMSO
7	Temperature	299.7
8	Pulse Sequence	zgpg30
9	Experiment	1D
10	Probe	5 mm PABBO BB-1H/ D Z-GRD Z104275/ 0252
11	Number of Scans	430
12	Receiver Gain	202.0
13	Relaxation Delay	2.0000
14	Pulse Width	10.0000
15	Acquisition Time	1.8088
16	Spectrometer Frequency	75.65
17	Spectral Width	18115.9
18	Lowest Frequency	-1494.1
19	Nucleus	13C
20	Acquired Size	32768

Figure 54. ^{13}C NMR (75 MHz, DMSO-d_6) spectrum of compound **2l**

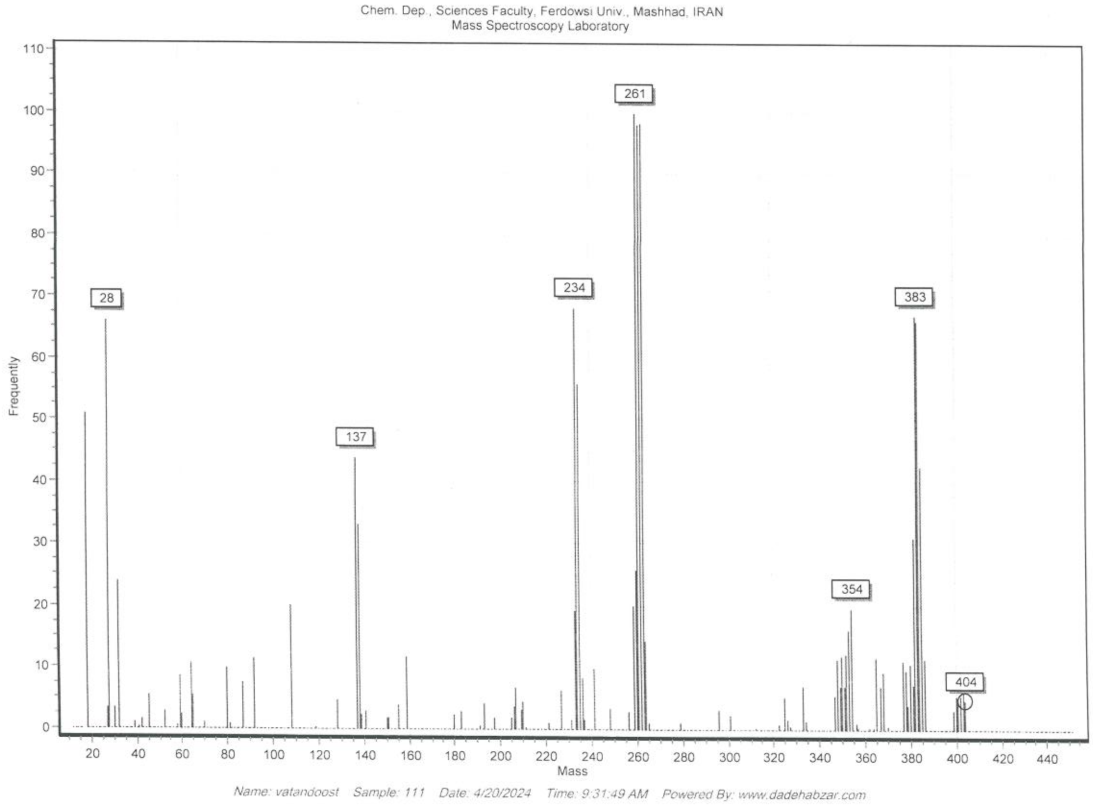

Figure 55. Mass spectrum of compound **2l**

Eager 300 Summarize Results

Date: 24/04/2024 at 13:03:43

Method Name: NCHS

Method Filename: Copy of N C H S-bkp.mth

Filename		As Method				Vial
Vatandoust-167						
# Group Sample Name		Tayp	Weig.	Prof. F	---	-----
167-1	111	UNK	0.699	6.25	---	-----
Component Name	Element%					

Nitrogen%	6.884370518
Carbon%	65.30261581
Hydrogen%	3.920376778
Sulphur%	0

1 Sample (s) in Group No:1

Component Name	Average
Nitrogen%	6.884370518
Carbon%	65.30261581
Hydrogen%	3.920376778
Sulphur%	0

Figure 56. CHNS spectrum of compound **2l**

Anal. Calcd. for $C_{22}H_{16}N_2O_4$ (404)		
C: 65.35 %	H: 3.99 %	N: 6.92 %

7-(5-bromo-2-hydroxyphenyl)-7,10,11,12-tetrahydro-6H-chromeno[4,3-b]quinoline-6,8(9H)-dione (2m)

Light yellow solid; (0.35g, 80%); Mp=308-310 °C; IR (KBr) (υ_{max}/cm^{-1}): 3348 (NH), 3101 (OH) 3050 (C-H aromatic), 2941 (C-H aliphatic), 1675 (C=O), 1608 (C=C); ^1H NMR (300 MHz, DMSO-d_6): δ (ppm): 9.83 (s, 1H, NH), 9.59 (s, 1H, OH), 8.35 (s, 1H, ArH, H_1), 7.65 (s, 1H, ArH, H_{18}), 7.43 (m, 2H, ArH, H_3,H_2), 7.19 (m, 2H, ArH, H_4,H_{16}), 6.71 (s, 1H, ArH, H_{15}), 4.97 (s, 1H, CH, H_7), 2.84-2.65 (m, 2H, CH_2, H_9), 2.31 (s, 2H, CH_2, H_{11}), 2.03-1.83 (m, 2H, CH_2, H_{10}); ^{13}C NMR (75 MHz, DMSO-d_6): δ (ppm): 196.46 (C_8), 160.75 (C_6), 155.24 (C_{14}), 153.47(C_{4a}), 152.55(C_{11a}), 143.34 (C_{12a}), 134.20 (C_3), 133.41(C_{16}), 132.41(C_{13}), 130.44 (C_{18}), 124.46 (C_1), 123.39 (C_2), 119.08 (C_{15}), 117.35 (C_4), 113.38 (C_{17}), 110.68 (C_{12b}), 110.52 (C_{7a}), 100.62 (C_{6a}), 36.98 (C_9), 32.52 (C_7), 26.93 (C_{11}), 21.09 (C_{10}); MS: (m/z, %):438 (M^+, 48), 436 (M^+-2, 38),420 (68),358 (5),266 (45),170 (35) 28 (100); Anal. Calcd. for $C_{22}H_{16}BrNO_4$ (438): C: 60.29, H: 3.68, N: 3.20%. Found: C: 60.18, H: 3.62, N: 3.11%.

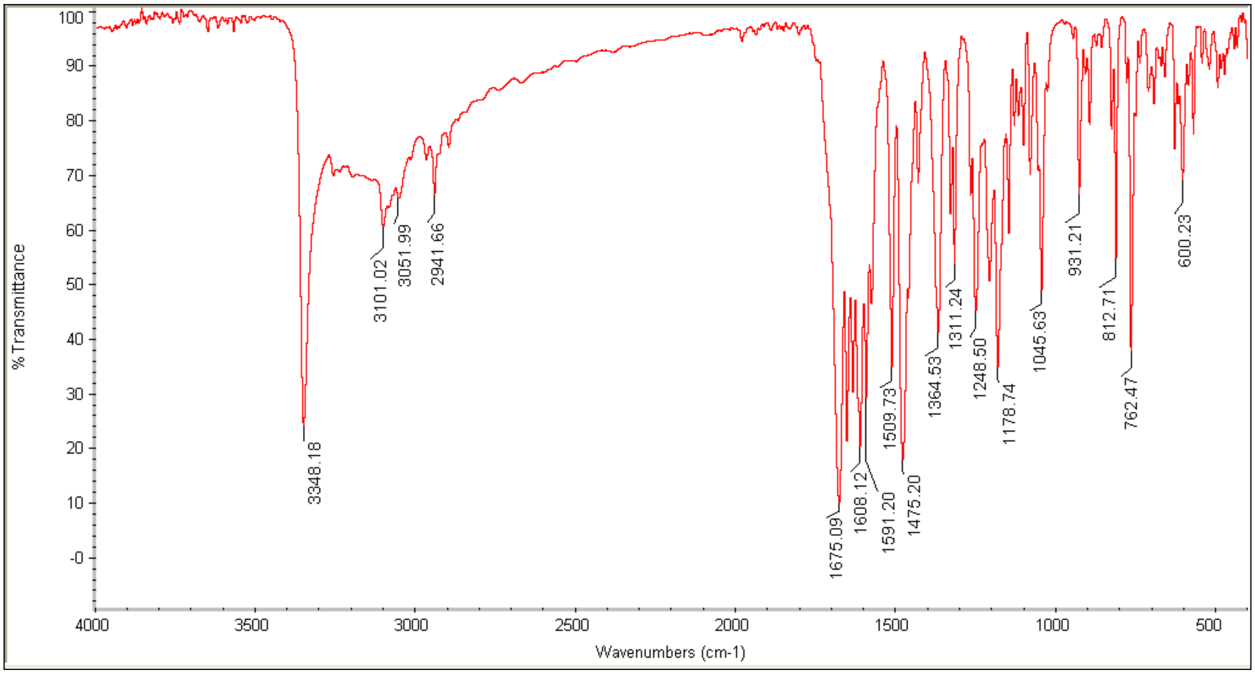

Figure 57. IR spectrum of compound **2m**

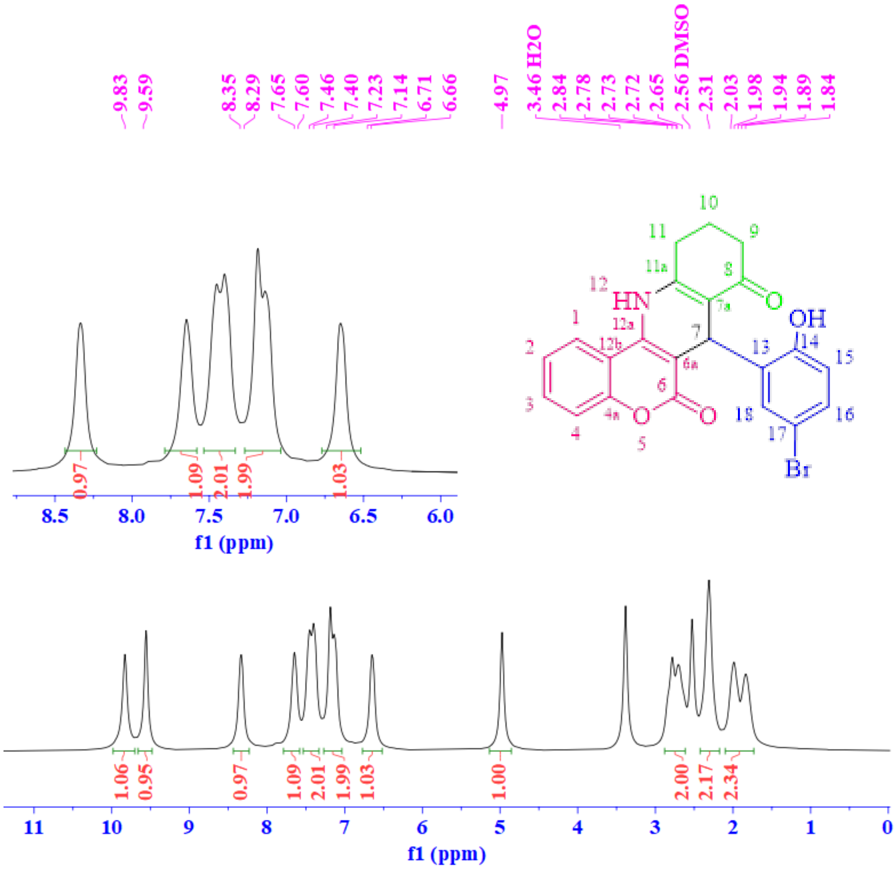

Parameter	Value
1 Title	Esfand.9.fid
2 Comment	Ahad vatandoust (2-OH-5-Br)
3 Origin	Bruker BioSpin GmbH
4 Owner	root
5 Instrument	spect
6 Solvent	DMSO
7 Temperature	297.0
8 Pulse Sequence	zg30
9 Experiment	1D
10 Probe	5 mm PABBO BB-1H/ D Z-GRD Z104275/ 0252
11 Number of Scans	136
12 Receiver Gain	33.2
13 Relaxation Delay	1.0000
14 Pulse Width	15.0000
15 Acquisition Time	0.9999
16 Spectrometer Frequency	300.81
17 Spectral Width	6009.6
18 Lowest Frequency	-1147.3
19 Nucleus	1H
20 Acquired Size	6009

Figure 58. ^{1}H NMR (300 MHz, DMSO-d_6) spectrum of compound **2m**

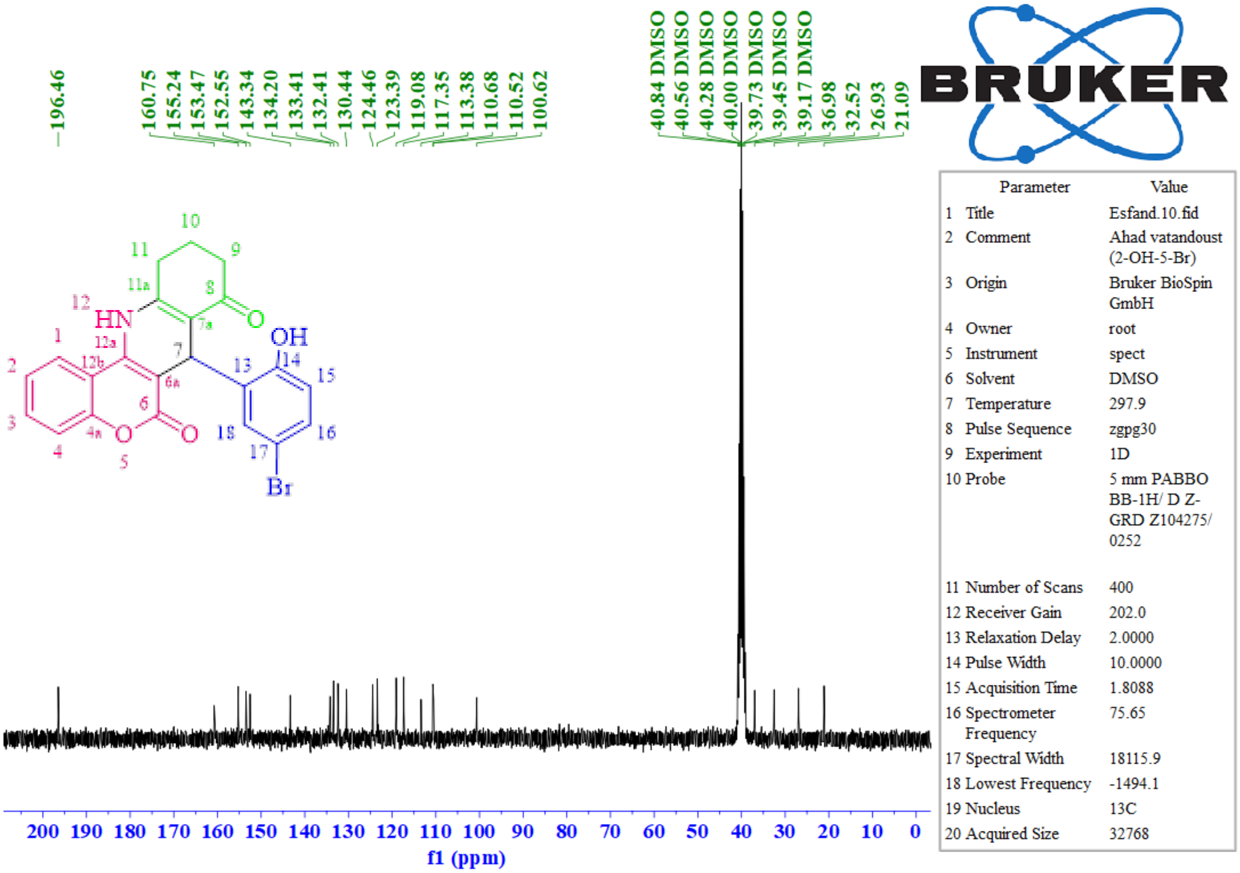

Figure 59. ^{13}C NMR (75 MHz, DMSO-d_6) spectrum of compound **2m**

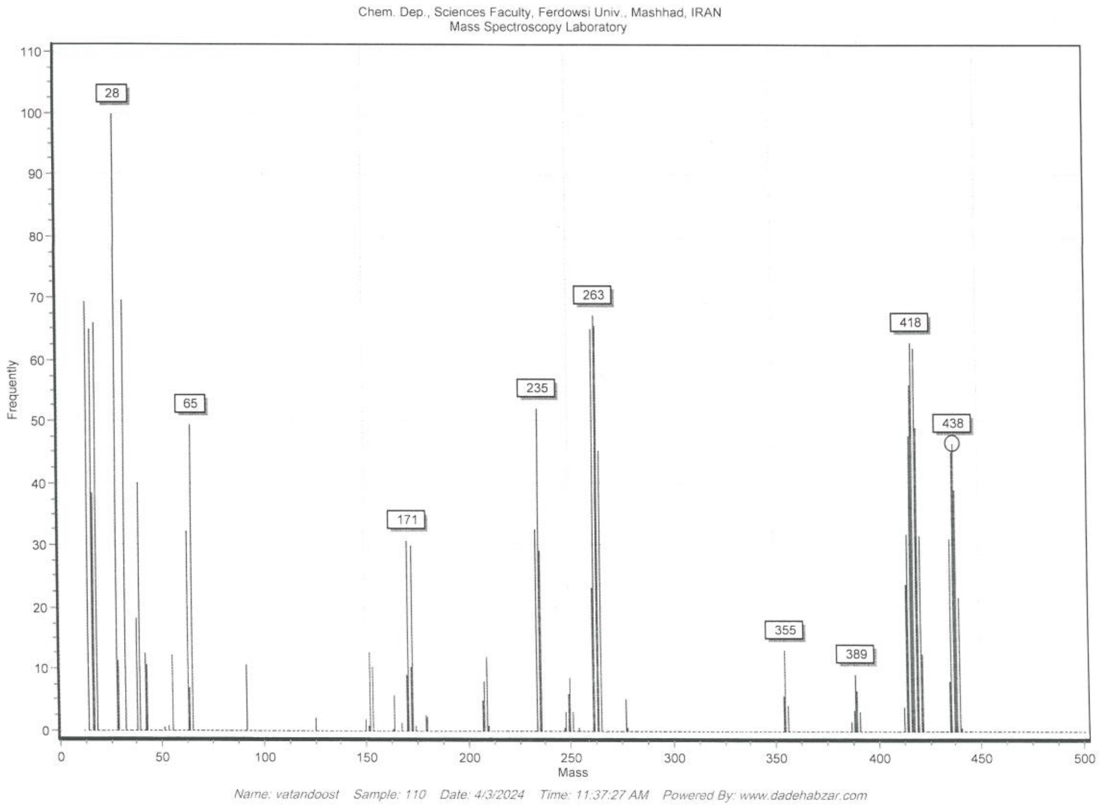

Figure 60. IR spectrum of compound **2m**

Eager 300 Summarize Results

Date: 24/04/2024 at 13:03:58

Method Name: NCHS

Method Filename: Copy of N C H S-bkp.mth

Filename		As Method				Vial
Vatandoust-168						
# Group Sample Name		Tayp	Weig.	Prof. F	---	-----
168-1	110	UNK	0.781	6.25	---	-----
Component Name	Element%					
Nitrogen%	3.117592270					
Carbon%	60.18610611					
Hydrogen%	3.628347397					
Sulphur%	0					

1 Sample (s) in Group No:1

Component Name	Average
Nitrogen%	3.117592270
Carbon%	60.18610611
Hydrogen%	3.628347397
Sulphur%	0

Figure 61. CHNS spectrum of compound **2m**

Anal. Calcd. for $C_{22}H_{16}BrNO_4$ (438)		
C: 60.29 %	H: 3.68 %	N: 3.20 %

7-(3,4-dihydroxyphenyl)-7,10,11,12-tetrahydro-6H-chromeno[4,3-b]quinoline-6,8(9H)-dione (2n)

White solid; (0.281g, 75%); Mp=310-311 °C; IR (KBr) (υ_{max}/cm^{-1}): 3460 (OH), 3320 (NH) 3084 (C-H aromatic), 2966, 2872 (C-H aliphatic), 1672 (C=O), 1633, 1604 (C=C); ^{1}H NMR (300 MHz, DMSO-d_6): δ (ppm): 9.69 (s, 1H, NH), 8.74 (s, 1H, OH), 8.59 (s, 1H, OH), 8.31 (d, J = 8.1 Hz, 1H, ArH, H_1), 7.63 (t, J = 7.8 Hz, 1H, ArH, H_3), 7.46-7.37 (m, 2H, ArH, H_2, H_4), 6.68 (s, 1H, ArH, H_{14}), 6.56 (d, J = 8.2 Hz, 1H, ArH, H_{17}), 6.49 (d, J = 8.3 Hz, 1H, ArH, H_{18}), 4.87 (s, 1H, CH, H_7), 2.87-2.80 (m, 1H, CH_2, H_9), 2.74-2.71 (m, 1H, CH_2, H_9), 2.38-2.25 (m, 2H, CH_2, H_{11}), 2.06-1.99 (m, 1H, CH_2, H_{10}), 1.94-1.87 (m, 1H, CH_2, H_{10}); ^{13}C NMR (75 MHz, DMSO-d_6): δ (ppm): 195.46 (C_8), 160.90 (C_6), 152.43 (C_{4a}), 151.55 (C_{11a}), 145.07(C_{12a}), 144.20 (C_{16}), 142.08 (C_{15}), 137.62 (C_{13}), 132.21 (C_3), 124.40 (C_1), 123.26 (C_2), 118.81 (C_{18}), 117.29 (C_4), 115.74 (C_{17}), 115.61 (C_{14}), 113.64 (C_{12b}), 112.78 (C_{7a}), 102.71(C_{6a}), 37.27 (C_9), 33.44 (C_7), 26.86 (C_{11}), 21.24 (C_{10}); MS: (m/z, %):(m/z, %):375 (M^+, 8), 373 (M^+-2, 35),266(64), 109(72) 28(100); Anal. Calcd. for $C_{22}H_{17}NO_5$ (375): C: 70.39, H: 4.56, N: 3.73%. Found: C: 70.22, H: 4.46, N: 3.60%.

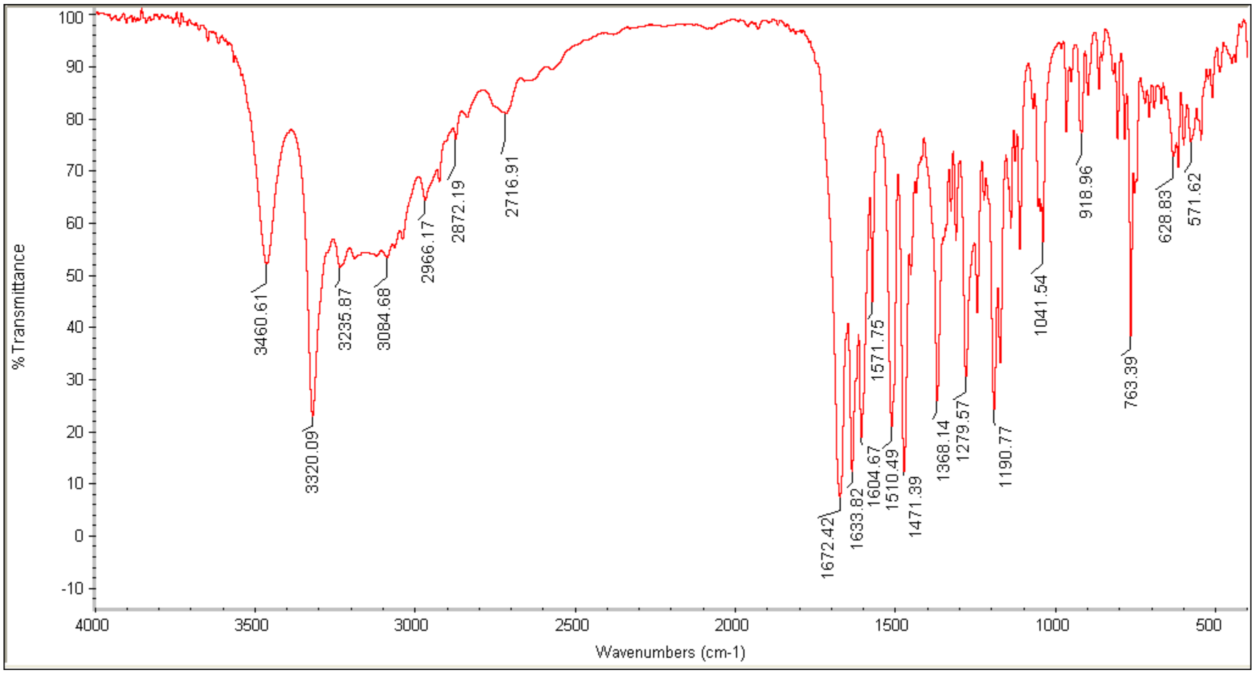

Figure 62. IR spectrum of compound **2n**

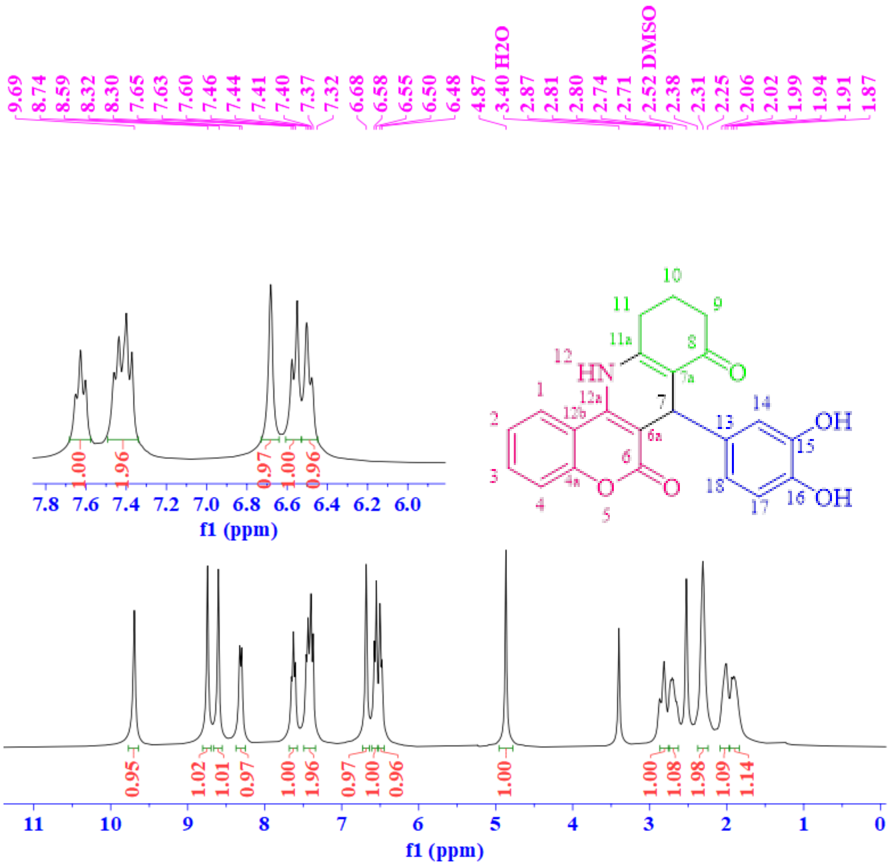

Parameter		Value
1	Title	Esfand.1.fid
2	Comment	Ahad vatandoust (3.4-di OH)
3	Origin	Bruker BioSpin GmbH
4	Owner	root
5	Instrument	spect
6	Solvent	DMSO
7	Temperature	296.5
8	Pulse Sequence	zg30
9	Experiment	1D
10	Probe	5 mm PABBO BB-1H/ D Z-GRD Z104275/ 0252
11	Number of Scans	146
12	Receiver Gain	127.3
13	Relaxation Delay	1.0000
14	Pulse Width	15.0000
15	Acquisition Time	0.9999
16	Spectrometer Frequency	300.81
17	Spectral Width	6009.6
18	Lowest Frequency	-1147.2
19	Nucleus	1H
20	Acquired Size	6009

Figure 63. ^{1}H NMR (300 MHz, DMSO-d_6) spectrum of compound **2n**

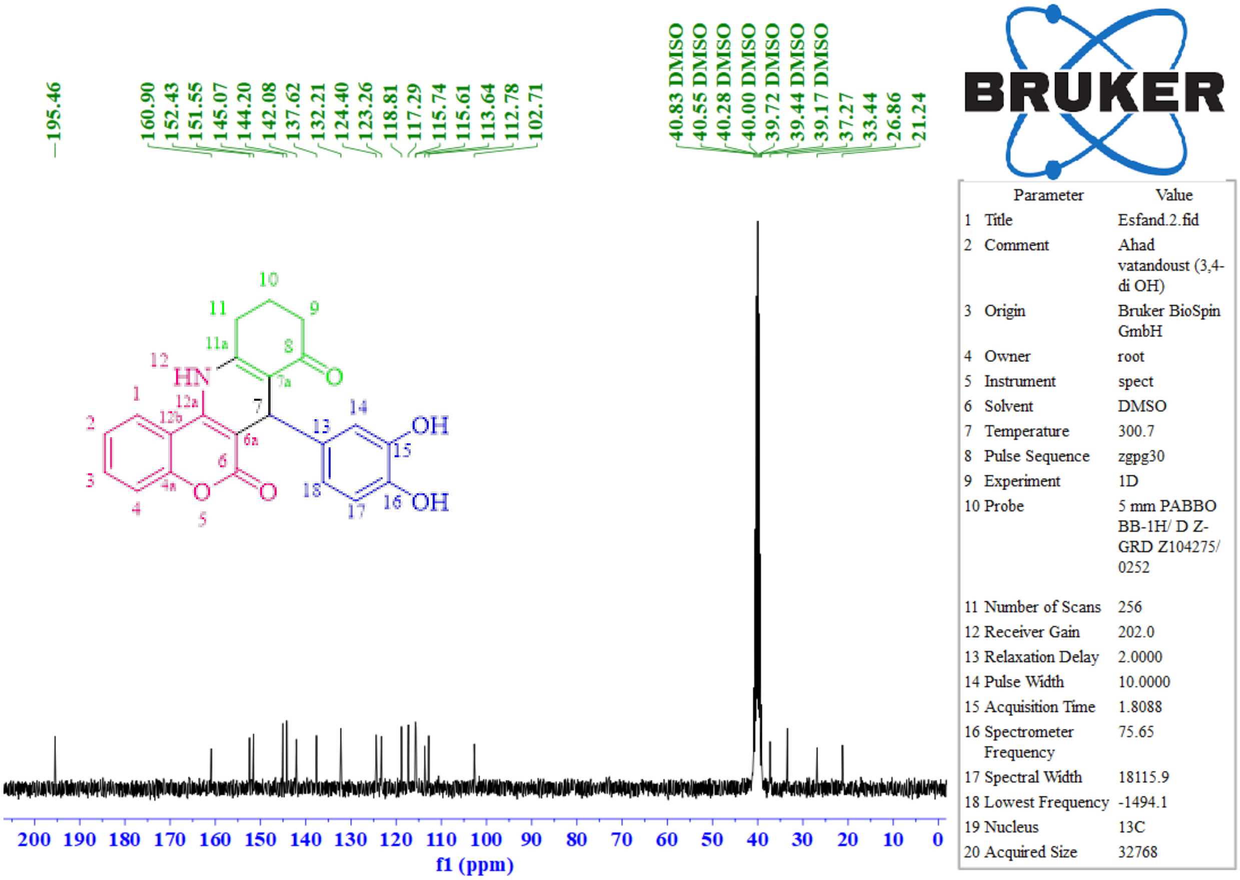

	Parameter	Value
1	Title	Esfand.2.fid
2	Comment	Ahad vatandoust (3,4-di OH)
3	Origin	Bruker BioSpin GmbH
4	Owner	root
5	Instrument	spect
6	Solvent	DMSO
7	Temperature	300.7
8	Pulse Sequence	zgpg30
9	Experiment	1D
10	Probe	5 mm PABBO BB-1H/ D Z-GRD Z104275/ 0252
11	Number of Scans	256
12	Receiver Gain	202.0
13	Relaxation Delay	2.0000
14	Pulse Width	10.0000
15	Acquisition Time	1.8088
16	Spectrometer Frequency	75.65
17	Spectral Width	18115.9
18	Lowest Frequency	-1494.1
19	Nucleus	13C
20	Acquired Size	32768

Figure 64. ^{13}C NMR (75 MHz, DMSO-d_6) spectrum of compound **2n**

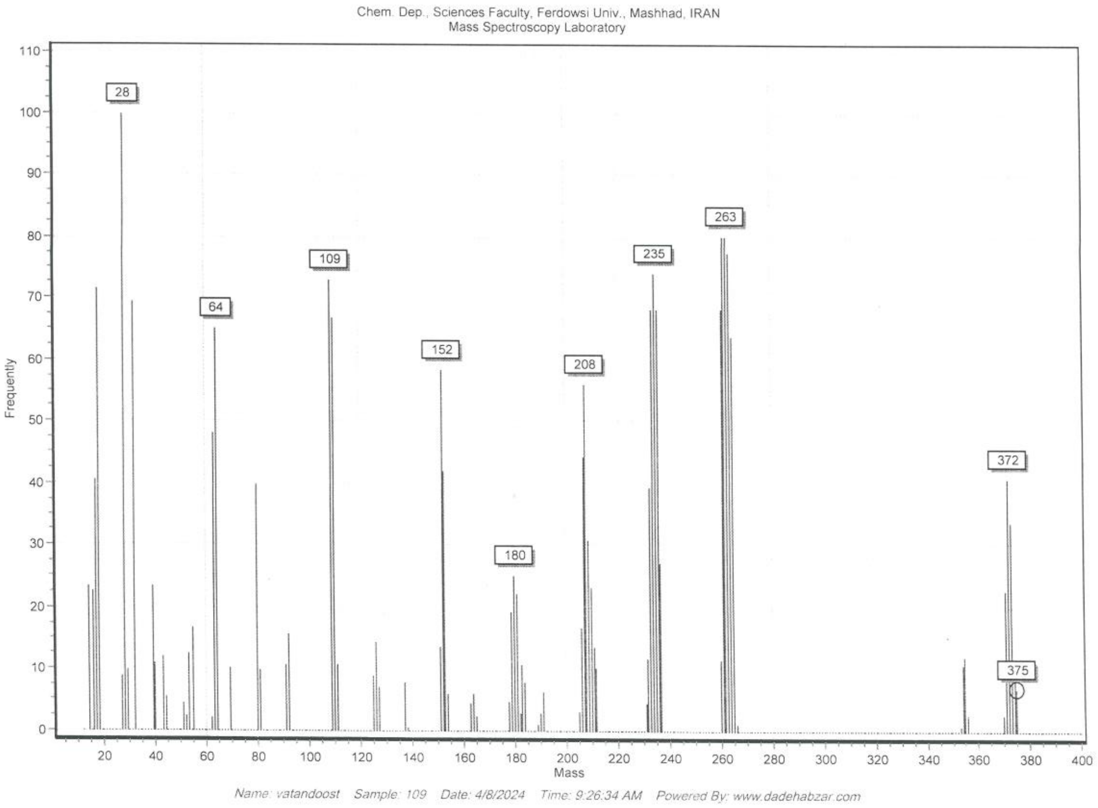

Figure 65. Mass spectrum of compound **2n**

Eager 300 Summarize Results

Date: 24/04/2024 at 13:04:08

Method Name: NCHS

Method Filename: Copy of N C H S-bkp.mth

Filename		As Method				Vial
Vatandoust-169						
# Group Sample Name		Tayp Weig. Prof. F			---	-----
169-1	109	UNK	0.653	6.25	---	-----
Component Name	Element%					
Nitrogen%	3.607834187					
Carbon%	70.22381805					
Hydrogen%	4.465614891					
Sulphur%	0					

1 Sample (s) in Group No:1

Component Name	Average
Nitrogen%	3.607834187
Carbon%	70.22381805
Hydrogen%	4.465614891
Sulphur%	0

Figure 66. CHNS spectrum of compound **2n**

Anal. Calcd. for $C_{22}H_{17}NO_5$ (375)		
C: 70.39 %	H: 4.56 %	N: 3.73 %

7-(thiophen-2-yl)-7,10,11,12-tetrahydro-6H-chromeno[4,3-b]quinoline-6,8(9H)-dione (2o)[1]

Dark brown solid; (0.279g, 80%); Mp=335-337 °C (Lit. 336-338 °C); IR (KBr) (υ_{max}/cm^{-1}): 3307 (NH) 3096 (C-H aromatic), 2958, 2880 (C-H aliphatic), 1701 (C=O), 1647, 1602 (C=C); ^{1}H NMR (300 MHz, DMSO-d_6): δ (ppm):9.91 (s, 1H, NH), 8.31 (d, J = 8.1 Hz, 1H, ArH, H_1), 7.67 (t, J = 7.8 Hz, 1H, ArH, H_3), 7.47-7.37 (m, 2H, ArH, H_2, H_4), 7.22 (s, 1H, ArH, H_{15}), 6.88-6.76 (m, 2H, ArH, H_{16}, H_{17}), 5.32 (s, 1H, CH, H_7), 2.90-2.82 (m, 1H, CH_2, H_9), 2.76-2.68 (m, 1H, CH_2, H_9), 2.40-2.34 (m, 2H, CH_2, H_{11}), 2.10-2.02 (m, 1H, CH_2, H_{10}), 1.98-1.93(m, 1H, CH_2, H_{10}); ^{13}C NMR (75 MHz, DMSO-d_6): δ (ppm): 195.33 (C_8), 160.87 (C_6), 152.48 (C_{4a}), 152.36 (C_{11a}), 149.93 (C_{12a}), 142.47 (C_{13}), 132.56 (C_3), 127.18 (C_{17}), 124.55 (C_1,C_{15}), 124.05 (C_{16}), 123.42 (C_2), 117.43 (C_4), 113.45 (C_{12b}), 111.85 (C_{7a}), 101.63 (C_{6a}), 37.12 (C_9), 29.32 (C_7), 26.83 (C_{11}), 21.24 (C_{10}); MS: (m/z, %): 349 (M^+, 38), 347 (M^+-2, 100), 266(98), 28(88); Anal. Calcd. for $C_{20}H_{15}NO_3S$ (349): C: 68.75, H: 4.33, N: 4.01, S: 9.18%. Found: C: 68.70, H: 4.29, N: 3.97, S: 9.12%.

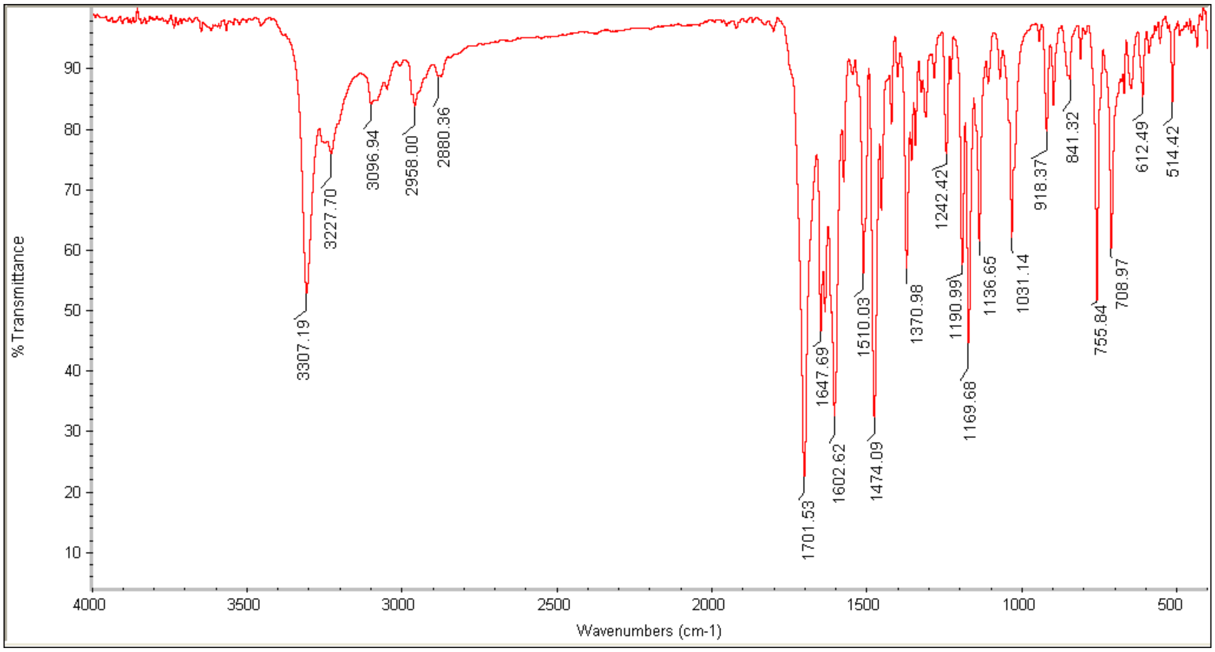

Figure 67. IR spectrum of compound **2o**

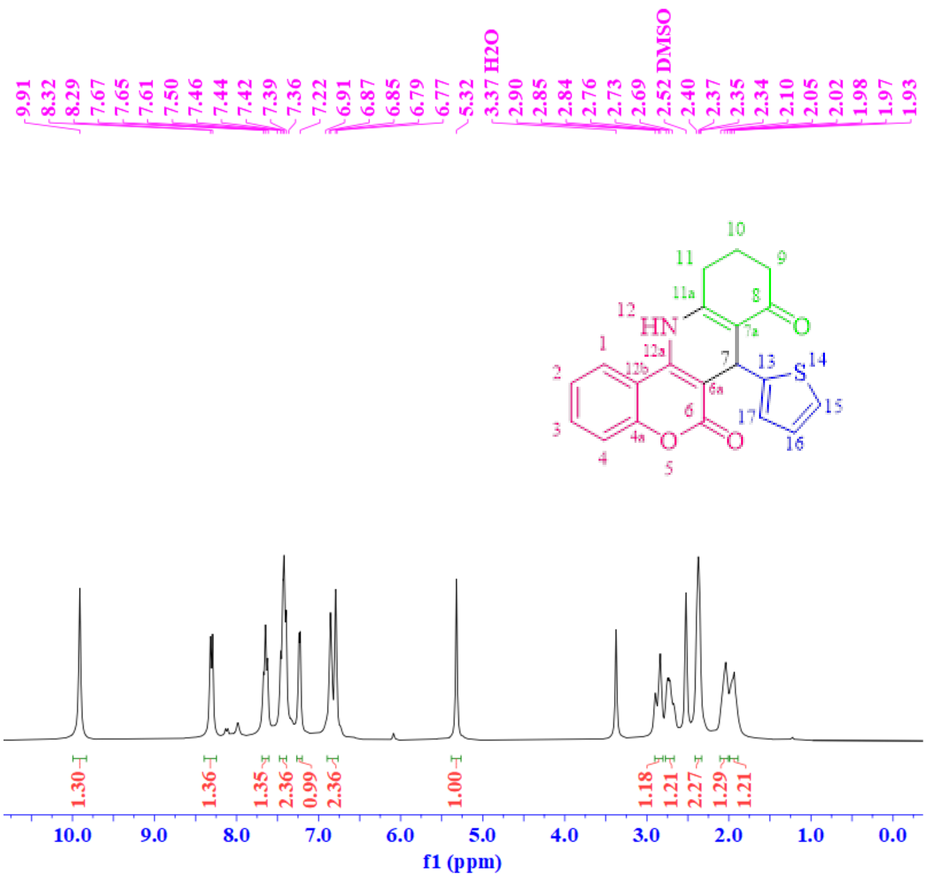

	Parameter	Value
1	Title	Esfand.51.fid
2	Comment	Ahad vatandoust (thiophene)
3	Origin	Bruker BioSpin GmbH
4	Owner	root
5	Instrument	spect
6	Solvent	DMSO
7	Temperature	297.6
8	Pulse Sequence	zg30
9	Experiment	1D
10	Probe	5 mm PABBO BB-1H/ D Z-GRD Z104275/ 0252
11	Number of Scans	24
12	Receiver Gain	33.2
13	Relaxation Delay	1.0000
14	Pulse Width	15.0000
15	Acquisition Time	0.9999
16	Spectrometer Frequency	300.81
17	Spectral Width	6009.6
18	Lowest Frequency	-1147.3
19	Nucleus	1H
20	Acquired Size	6009

Figure 68. ^{1}H NMR (300 MHz, DMSO-d_6) spectrum of compound **2o**

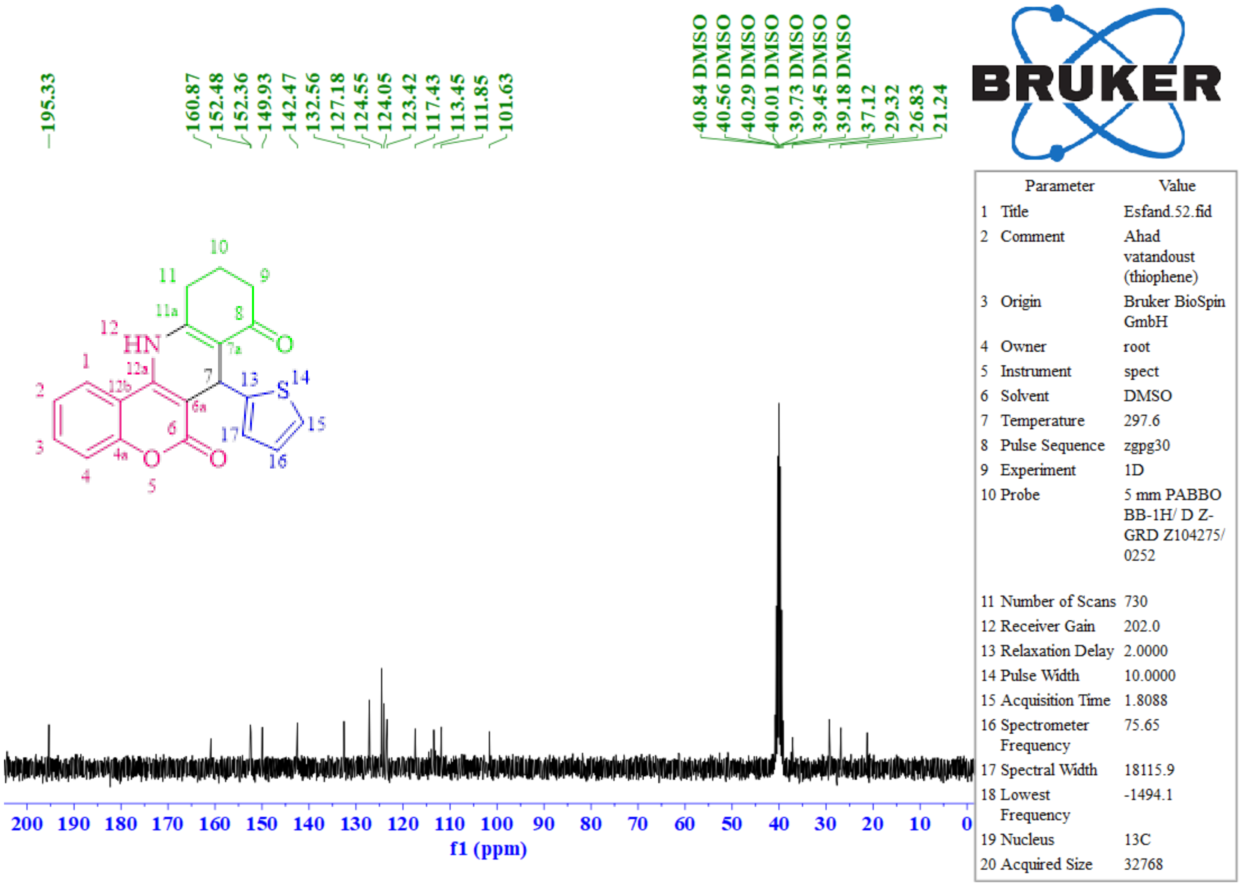

	Parameter	Value
1	Title	Esfand.52.fid
2	Comment	Ahad vatandoust (thiophene)
3	Origin	Bruker BioSpin GmbH
4	Owner	root
5	Instrument	spect
6	Solvent	DMSO
7	Temperature	297.6
8	Pulse Sequence	zgpg30
9	Experiment	1D
10	Probe	5 mm PABBO BB-1H/ D Z-GRD Z104275/ 0252
11	Number of Scans	730
12	Receiver Gain	202.0
13	Relaxation Delay	2.0000
14	Pulse Width	10.0000
15	Acquisition Time	1.8088
16	Spectrometer Frequency	75.65
17	Spectral Width	18115.9
18	Lowest Frequency	-1494.1
19	Nucleus	13C
20	Acquired Size	32768

Figure 69. ^{13}C NMR (75 MHz, DMSO-d_6) spectrum of compound **2o**

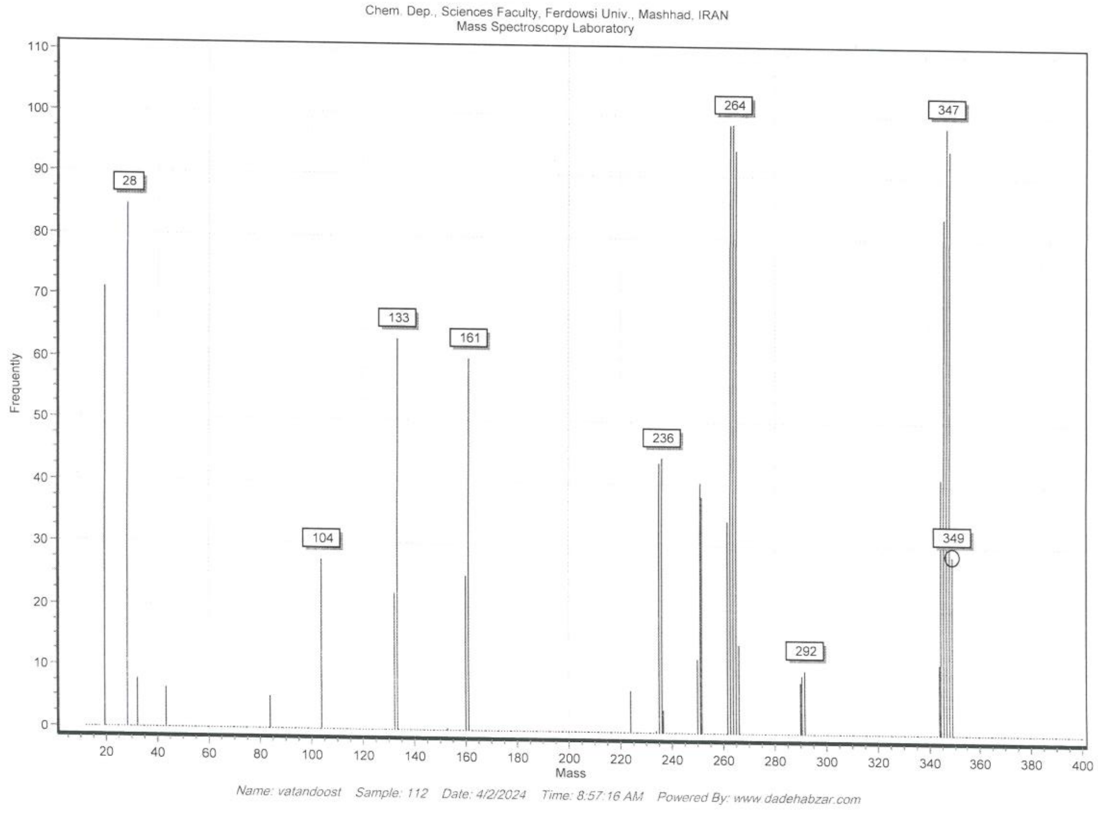

Figure 70. Mass spectrum of compound **2o**

Eager 300 Summarize Results

Date: 24/04/2024 at 13:02:16

Method Name: NCHS

Method Filename: Copy of N C H S-bkp.mth

Filename		As Method				Vial
Vatandoust-162						
# Group Sample Name		Tayp Weig. Prof. F			---	-----
162-1	112	UNK	0.651	6.25	---	-----

Component Name	Element%
Nitrogen%	3.979906257
Carbon%	68.70428131
Hydrogen%	4.291711521
Sulphur%	9.12617751

1 Sample (s) in Group No:1

Component Name	Average
Nitrogen%	3.979906257
Carbon%	68.70428131
Hydrogen%	4.291711521
Sulphur%	9.12617751

Figure 71. CHNS spectrum of compound **2o**

Anal. Calcd. for $C_{20}H_{15}NO_3S$ (349)			
C: 68.75%	H: 4.33 %	N: 4.01 %	S: 9.18 %

7-(furan-2-yl)-7,10,11,12-tetrahydro-6H-chromeno[4,3-b]quinoline-6,8(9H)-dione (2p)[1]

Gray solid; (0.266g, 80%); Mp=302-304 ºC (Lit. 303-305 ºC); IR (KBr) (υ_{max}/cm^{-1}): 3324 (NH) 3096 (C-H aromatic), 2944, 2872 (C-H aliphatic), 1676 (C=O), 1643, 1607 (C=C); ^{1}H NMR (300 MHz, DMSO-d_6): δ (ppm): 9.86 (s, 1H, NH), 8.31 (d, J = 8.0 Hz, 1H, ArH, H_1), 7.67 (t, J = 7.8 Hz, 1H, ArH, H_3), 7.48-7.41 (m, 3H, H_2, H_4, H_{15}), 6.29 (s, 1H, ArH, H_{17}), 6.02 (s, 1H, ArH, H_{16}), 5.18 (s, 1H, CH, H_7), 2.89-2.80 (m, 1H, CH_2, H_9), 2.75-2.65 (m, 1H, CH_2, H_9), 2.38-2.36 (m, 2H, CH_2, H_{11}), 2.08-2.00 (m, 1H, CH_2, H_{10}), 1.99-1.90 (m, 1H, CH_2, H_{10}); ^{13}C NMR (75 MHz, DMSO-d_6): δ (ppm): 195.36 (C_8), 160.74 (C_6), 156.97 (C_{13}), 152.93 (C_{4a}), 152.49 (C_{11a}), 143.13 (C_{12a}), 141.90 (C_{15}), 132.62 (C_3), 124.57 (C_1), 123.32 (C_2), 117.44 (C_4), 113.46 (C_{12b}), 110.89 (C_{7a}), 109.42 (C_{16}), 105.78 (C_{17}), 99.14 (C_{6a}), 37.04 (C_9), 28.41 (C_7), 26.85 (C_{11}), 21.18 (C_{10}); MS: (m/z, %): 333 (M^+, 38), 331 (M^+-2, 86), 225(22), 28(10); Anal. Calcd. for $C_{20}H_{15}NO_4$ (333): C: 72.06, H: 4.54, N: 4.20 %. Found: C: 71.96, H: 4.46, N: 4.16%.

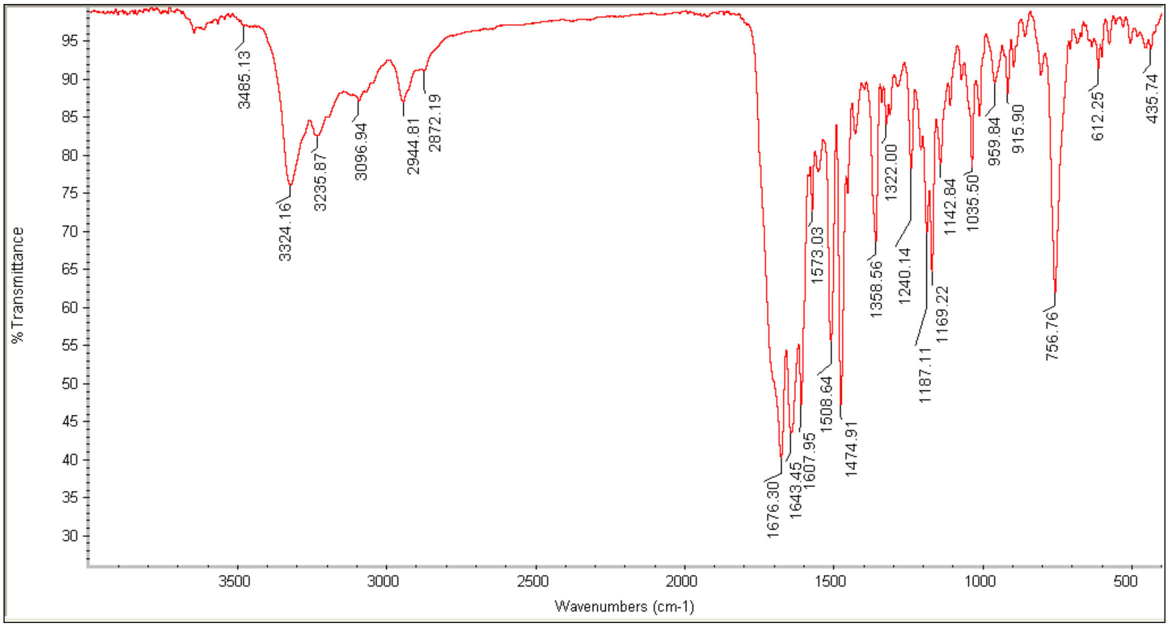

Figure 72. IR spectrum of compound **2p**

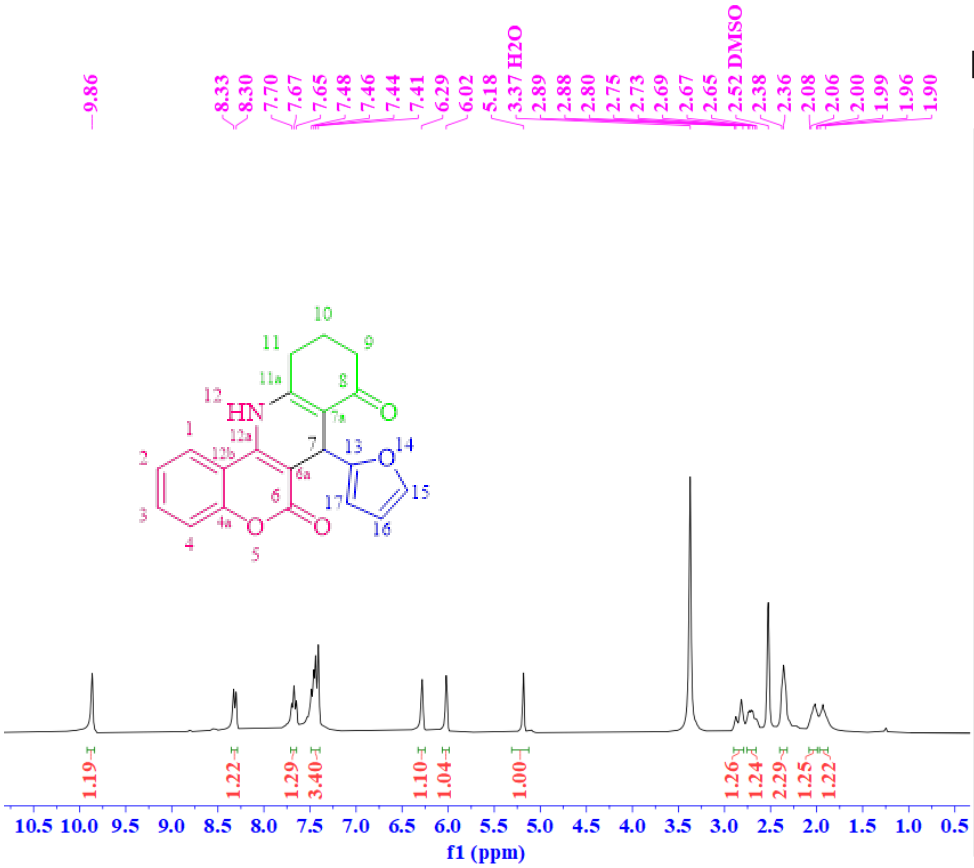

	Parameter	Value
1	Title	Farvardin. 639.fid
2	Comment	Ahad vatandoust (Furfural)
3	Origin	Bruker BioSpin GmbH
4	Owner	root
5	Instrument	spect
6	Solvent	DMSO
7	Temperature	295.2
8	Pulse Sequence	zg30
9	Experiment	1D
10	Probe	5 mm PABBO BB-1H/ D Z-GRD Z104275/ 0252
11	Number of Scans	64
12	Receiver Gain	202.0
13	Relaxation Delay	1.0000
14	Pulse Width	15.0000
15	Acquisition Time	0.9999
16	Spectrometer Frequency	300.81
17	Spectral Width	6009.6
18	Lowest Frequency	-1147.3
19	Nucleus	1H
20	Acquired Size	6009

Figure 73. ^{1}H NMR (300 MHz, DMSO-d_6) spectrum of compound **2p**

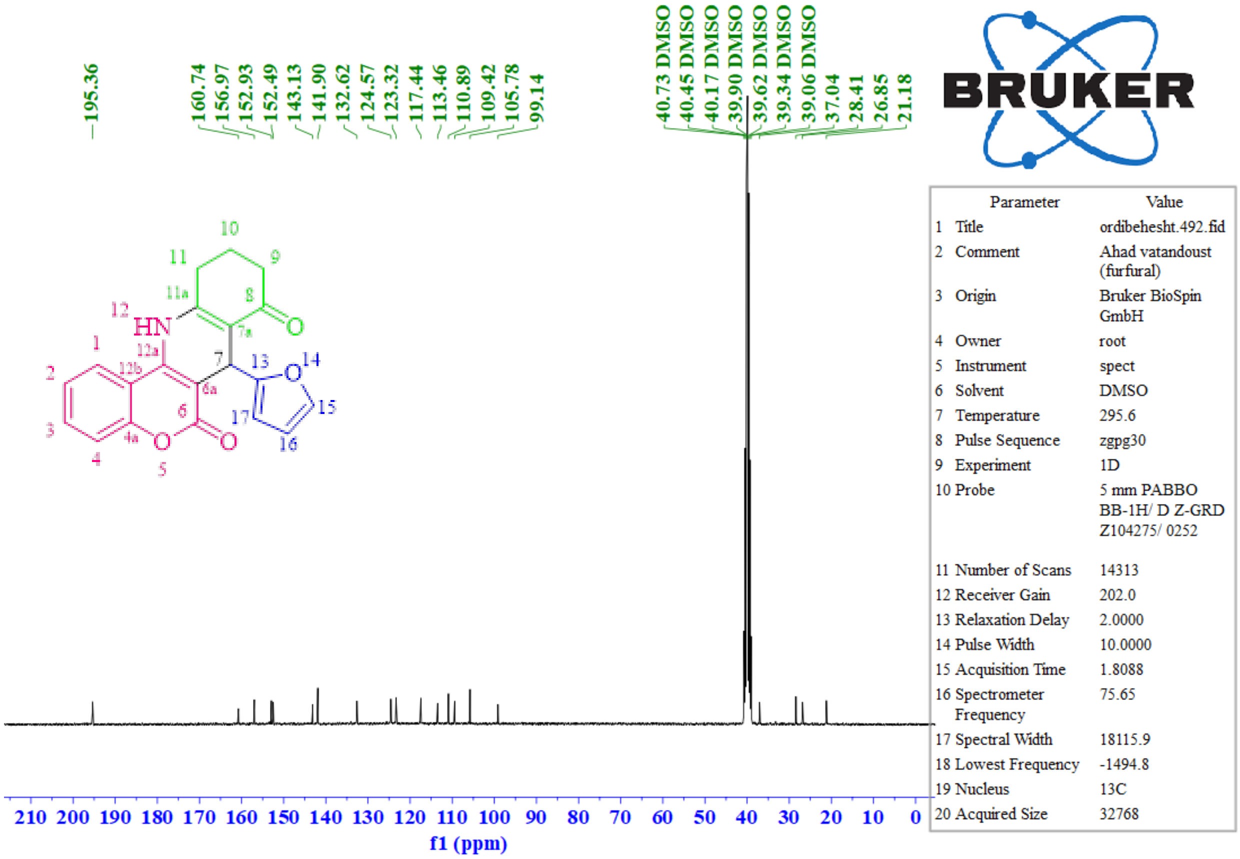

	Parameter	Value
1	Title	ordibehesht.492.fid
2	Comment	Ahad vatandoust (furfural)
3	Origin	Bruker BioSpin GmbH
4	Owner	root
5	Instrument	spect
6	Solvent	DMSO
7	Temperature	295.6
8	Pulse Sequence	zgpg30
9	Experiment	1D
10	Probe	5 mm PABBO BB-1H/ D Z-GRD Z104275/ 0252
11	Number of Scans	14313
12	Receiver Gain	202.0
13	Relaxation Delay	2.0000
14	Pulse Width	10.0000
15	Acquisition Time	1.8088
16	Spectrometer Frequency	75.65
17	Spectral Width	18115.9
18	Lowest Frequency	-1494.8
19	Nucleus	13C
20	Acquired Size	32768

Figure 74. ^{13}C NMR (75 MHz, DMSO-d_6) spectrum of compound **2p**

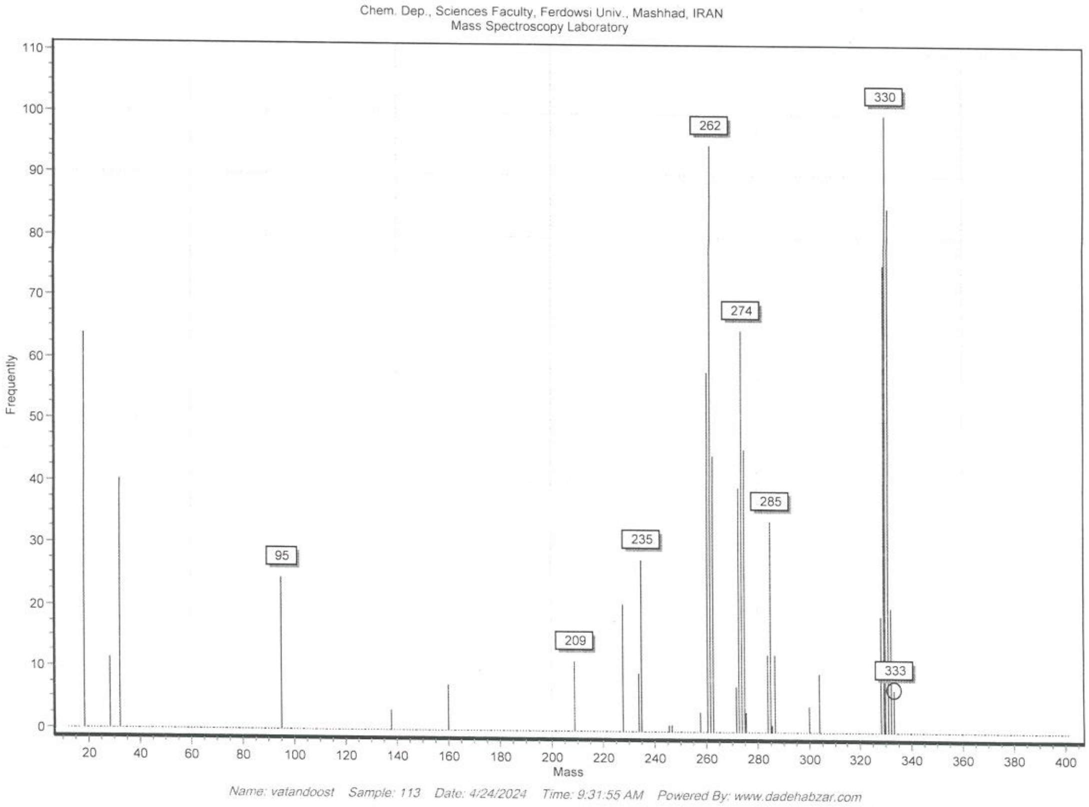

Figure 75. Mass spectrum of compound **2p**

Eager 300 Summarize Results

Date: 24/04/2024 at 13:04:39

Method Name: NCHS

Method Filename: Copy of N C H S-bkp.mth

Filename		As Method				Vial
Vatandoust-172						
# Group Sample Name		Tayp	Weig.	Prof. F	---	-----
172-1	113	UNK	0.602	6.25	---	-----

Component Name	Element%
Nitrogen%	4.160822124
Carbon%	71.96075546
Hydrogen%	4.46627367
Sulphur%	0

1 Sample (s) in Group No:1

Component Name	Average
Nitrogen%	4.160822124
Carbon%	71.96075546
Hydrogen%	4.46627367
Sulphur%	0

Figure 76. CHNS spectrum of compound **2p**

Anal. Calcd. for $C_{20}H_{15}NO_4$ (333)		
C: 72.06 %	H: 4.54 %	N: 4.20 %

Refrences:

[1] N. Ahmed, B. V. Babu, S. Singh and P. M. Mitrasinovic, *Heterocycles* 2012, **85**, 1629-1653.

[2] C. J. Hua, K. Zhang, M. Xin, T. Ying, J. R. Gao, J. h. Jia and Y. j. Li, *RSC adv.,* 2016, ***6***, 49221-49227.

[3] R. Motamedi, G. R. Bardajee and S. Shakeri, *Heterocycl. Commun.*, 2014, **20**, 181-184.

[4] C. J. Hua, H. Zheng, K. Zhang, M. Xin, J. R. Gao and Y. J. Li, *Tetrahedron*, 2016, **72**, 8365-8372.

[5] R. Miri, R. Motamedi, M. R. Rezaei, O. Firuzi, A. Javidnia and A. Shafiee, *Arch. Pharm.*, 2011, **344**, 111-118.
